# Supplementary material for: Prevalence and social determinants of anxiety and depression among adults in Ghana: a systematic review and meta-analysis protocol
Source: BMJ Open. 2024 Apr 23;14(4):e081927. doi: 10.1136/bmjopen-2023-081927 (PMC11043746; doi:10.1136/bmjopen-2023-081927)
Supplement: Supplementary data [file bmjopen-2023-081927supp002.pdf]

Supplementary Appendix 2

PRESS Guideline — Search Submission & Peer Review Assessment

SEARCH SUBMISSION: THIS SECTION TO BE FILLED IN BY THE SEARCHER

Searcher: Victoria Awortwe	Email: victoria.awortwe@uu.se
Date submitted: 31/8/2023	Date requested by:11/9/2023

Systematic Review Title:

Prevalence and social determinants of anxiety and depression among adults in Ghana: a systematic review and meta-analysis protocol

This search strategy is …

X	My PRIMARY (core) database strategy — First time submitting a strategy for search question and database
	My PRIMARY (core) strategy — Follow-up review NOT the first time submitting a strategy for search question and database. If this is a response to peer review, itemize the changes made to the review suggestions
	SECONDARY search strategy— First time submitting a strategy for search question and database
	SECONDARY search strategy — NOT the first time submitting a strategy for search question and database. If this is a response to peer review, itemize the changes made to the review suggestions

Database

(i.e., MEDLINE, CINAHL…): *[mandatory]*

MEDLINE

Interface

(i.e., Ovid, EBSCO…): *[mandatory]*

PubMed

Research Question

(Describe the purpose of the search) *[mandatory]*

Awortwe V, *et al. BMJ Open* 2024; 14:e081927. doi: 10.1136/bmjopen-2023-081927

This review aims (1) examine the prevalence of anxiety, depression, and psychological distress among adults in Ghana and (2) explore the social determinants potentially associated with anxiety, depression and psychological distress.

CoCoPop Format

(Outline the CoCoPop for your question — i.e., Condition, Context, Population, and Study Design — as applicable)

**Population**	Adults (individuals aged ≥ 18 years) living in Ghana
**Condition**	Anxiety (i.e. anxiety disorders, anxiety symptoms), depression (i.e. depression, depressive disorders), and psychological distress.
**Context**	Ghana, with adults sampled from the community or clinical settings (e.g., primary health care facilities, hospitals).
**Study design**	Observational study designs

Inclusion Criteria

(List criteria such as age groups, study designs, etc., to be included) *[optional]*

Population: Adults (aged ≥ 18 years) living in Ghana will be eligible for inclusion.

Condition: Anxiety and/or depression assessed using structured diagnostic clinical interview in accordance with the International Classification of Diseases and Related Health Problems (ICD-10 or ICD-11), or the Diagnostic and Statistical Manual of Mental disorders (DSM) third, fourth and fifth edition (DSM-III, DSM-IV or DSM-V); OR clinician or proxy administered screening tool for anxiety, depression and psychological distress. For the meta-analyses, studies reporting on the prevalence of anxiety, depression and psychological distress among adults in Ghana, measured using validated instruments will be included.

Context: Studies from Ghana, with adults sampled from the community or clinical settings (e.g., primary health care facilities, hospitals). Studies conducted in regions that encompass Ghana (e.g., West Africa and Sub-Saharan Africa) will be considered eligible if data on participants living in Ghana is available. No date restriction will be imposed and only studies published in English and Ghanaian languages (e.g., Fante, Ga, Twi) will be considered for inclusion.

Study design: Primary quantitative studies, with observational study designs including longitudinal cohort studies, case-control, and cross-sectional studies. Mixed method studies will be eligible for inclusion only if data from the quantitative component can be clearly extracted.

Exclusion Criteria

(List criteria such as study designs, date limits, etc., to be excluded) *[optional]*

Population: Studies solely focused on children/adolescents will be excluded. Studies focusing on both adults and adolescents will be excluded if they do not present data separately for adults and adolescents or data cannot be obtained via correspondence with study authors. Studies conducted with specific sub-populations including individuals with known psychiatric conditions, prisoners, individuals accused of witchcraft, and women with fertility or gynaecological disorders, will be excluded due to their unique circumstances, which predispose them to a higher risk or potentially elevate the likelihood of experiencing mental health problems compared to the general population.

Condition: Studies solely focused on other mental disorders (e.g., psychotic disorders, bipolar affective disorders), substance use and neurological disorders (e.g., alcohol dependence, multiple sclerosis) will be excluded. Studies will be excluded if the prevalence of anxiety, depression and psychological distress cannot be calculated, e.g., when reported solely as mean score or due to insufficient data.

Context: Studies that were conducted immediately after conflict, humanitarian crises, or natural disaster will be excluded.

Study design: Studies such as case reports, commentaries, conference proceedings, editorials, letters, opinion papers, qualitative studies, reviews, theses/dissertations; and studies published in languages other than English and Ghanaian will be excluded.

Was a search filter applied?

Yes ☐ No ■

If YES, which one(s) (e.g., Cochrane RCT filter, PubMed Clinical Queries filter)? Provide the source if this is a published filter. *[mandatory if YES to previous question — textbox]*

Other notes or comments you feel would be useful for the peer reviewer? ***[optional]***

We did not include search terms regarding (1) prevalence, as that is not the only area of interest; and (2) adults, as the term can be referred to in a variety of ways. Furthermore, we searched Ghana in All fields to accommodate studies conducted in regions that encompass Ghana (e.g., West Africa and Sub-Saharan Africa) providing data on participants living in Ghana.

Please copy and paste your search strategy here, exactly as run, including the number of hits per line. ***[mandatory]***

Database: PubMed. Final number of results from the full search 1297	
**1: Mental disorders**	**2: Ghana**
**Title & Abstract**<br>Mental health 228145<br>Mental disorders 51217<br>Mental disorder 11824<br>Mental illness 37257<br>Mental illnesses 6045<br>Anxiety 265049<br>Anxious 20603<br>Depress* 569151<br>Dysthymia 2320<br>Melancholy 426<br>Mood 92155<br>Affective disorder 7725<br>Affective symptoms 2417<br>Negative affect 13573<br>distress 154761<br>Emotional stress 5232<br><br>**MeSH terms**<br>Mental health 61933<br>Mental disorders 1436875<br>Anxiety 112111<br>Anxiety disorders 90630<br>Depression 257186<br>Depressive disorder 122190<br>Mood disorders 170032<br>Psychological Distress 6832	**All fields**<br>Ghana 24845<br><br>**MeSH term**<br>Ghana 11236
**Total for concept 1:**	**Total for concept 2:**
**2183845**	**25013**

Total for complete search: 1297

Raw PubMed Search

("mental health"[Title/Abstract] OR "mental disorders"[Title/Abstract] OR "mental disorder"[Title/Abstract] OR "mental illness"[Title/Abstract] OR "mental illnesses"[Title/Abstract] OR "anxiety"[Title/Abstract] OR "anxious"[Title/Abstract] OR "depress*"[Title/Abstract] OR "dysthymia"[Title/Abstract] OR "melancholy"[Title/Abstract] OR "mood"[Title/Abstract] OR "affective disorder"[Title/Abstract] OR "affective symptom"[Title/Abstract] OR "negative affect"[Title/Abstract] OR "distress"[Title/Abstract] OR "emotional stress"[Title/Abstract] OR ("mental health"[MeSH Terms] OR "mental disorders"[MeSH Terms] OR "anxiety"[MeSH Terms] OR "anxiety disorders"[MeSH Terms] OR ("depressive disorder"[MeSH Terms] OR "depression"[MeSH Terms]) OR "depressive disorder"[MeSH Terms] OR "mood disorders"[MeSH Terms] OR "psychological distress"[MeSH Terms])) AND ("Ghana"[MeSH Terms] OR "Ghana"[All Fields] OR "Ghana s"[All Fields] OR "Ghana"[MeSH Terms]).

PEER REVIEW ASSESSMENT: THIS SECTION TO BE FILLED IN BY THE REVIEWER

Reviewer: Professor Alkistis Skalkidou	Email: Alkistis.skalkidou@kbh.uu.se	Date completed: 14/9/2023

1. TRANSLATION

A ---No revisions	■
B --- Revision(s) suggested	☐
C --- Revision(s) required	☐

If “B” or “C,” please provide an explanation or example:

Does the search strategy match the research question/PICO?
Are the search concepts clear?
Are there too many or too few PICO elements included?
Are the search concepts too narrow or too broad?
Does the search retrieve too many or too few records? (Please show number of hits per line.)
Are unconventional or complex strategies explained

2. BOOLEAN AND PROXIMITY OPERATORS

A ---No revisions	■
B --- Revision(s) suggested	☐
C --- Revision(s) required	☐

If “B” or “C,” please provide an explanation or example:

Are Boolean or proximity operators used correctly?
Is the use of nesting with brackets appropriate and effective for the search?
If NOT is used, is this likely to result in any unintended exclusions?
Could precision be improved by using proximity operators (eg, adjacent, near, within) or phrase searching instead of AND?
Is the width of proximity operators suitable (eg, might adj5 pick up more variants than adj2)?
Not used

3. SUBJECT HEADINGS

A ---No revisions	☐
B --- Revision(s) suggested	■
C --- Revision(s) required	☐

If “B” or “C,” please provide an explanation or example:

6

Are the subject headings relevant?
Are any subject headings too broad or too narrow?
Are subject headings exploded where necessary and vice versa?
Are major headings ("starring" or restrict to focus) used? If so, is there adequate justification?
Are subheadings missing?
Are subheadings attached to subject headings? (Floating subheadings may be preferred.)
Are floating subheadings relevant and used appropriately?
Are both subject headings and terms in free text (see the following) used for each concept?

4. TEXT WORD SEARCHING

A ---No revisions	■
B --- Revision(s)suggested	☐
C --- Revision(s) required	☐

If "B" or "C," please provide an explanation or example:

Does the search include all spelling variants in free text (eg, UK vs. US spelling)?
Does the search include all synonyms or antonyms (eg, opposites)?
Does the search capture relevant truncation (i.e., is truncation at the correct place)?
Is the truncation too broad or too narrow?
Are acronyms or abbreviations used appropriately? Do they capture irrelevant material? Are the full terms also included?
Have the appropriate fields been searched; for example, is the choice of the text word fields (.tw.) or all fields (.af.) appropriate? Are there any other fields to be included or excluded (database specific)?
Should any long strings be broken into several shorter search statements?

5. SPELLING, SYNTAX, AND LINE NUMBERS

A ---No revisions	■
B --- Revision(s)suggested	☐
C --- Revision(s) required	☐

If "B" or "C," please provide an explanation or example:

Are there any spelling errors?
Are there errors in system syntax (e.g. the use of a truncation symbol from a different search interface)?
Are there incorrect line combinations or orphan lines (e.g. lines that are not referred to in the final summation that?
could indicate an error in an AND or OR statement)?

6. LIMITS AND FILTERS

A ---No revisions	☐

Awortwe V, *et al. BMJ Open* 2024; 14:e081927. doi: 10.1136/bmjopen-2023-081927

B --- Revision(s) suggested	■
C --- Revision(s) required	☐

If "B" or "C," please provide an explanation or example:

Are all limits and filters used appropriately and are they relevant given the research question?

Are all limits and filters used appropriately and are they relevant for the database?

Are any potentially helpful limits or filters missing? Are the limits or filters too broad or too narrow? Can any limits

or filters be added or removed? Language? Time period? Only humans?

Are sources cited for the filters used?

Author response:

Thanks for the suggestion. Databases will be searched from inception, and the time period for data collection (e.g., <2020 vs. ≥2020) will be investigated as a methodological moderator of prevalence utilising subgroup analyses and meta-regression. Only human studies and language search filters were not used. However, non-human studies and studies published in languages other than English and Ghanaian would be excluded during the screening phase.

OVERALL EVALUATION (Note: If one or more "revision required" is noted above, the response below must be "revisions required".)

A ---No revisions	☐
B --- Revision(s) suggested	■
C --- Revision(s) required	☐

Additional comments:

I am not familiar with Medline, but many within psychology also search the PSYCHINFO database

Author response:

Thank you for your feedback. MEDLINE (PubMed) is one of the electronic databases in which the searches will be conducted. However, electronic database searches will be also carried out in African Index Medicus (AIM), African Journals Online (AJOL), Cumulative Index to Nursing and Allied Health Literature (CINAHL), Excerpta Medica Database (Embase), Ghana Medical Journal (GMJ), Health Sciences Investigation (HIS), **PsycINFO** and SCOPUS.

PEER REVIEW ASSESSMENT #2: THIS SECTION TO BE FILLED IN BY THE REVIEWER

Reviewer: Associate Professor Lene Lindberg	Email: lene.lindberg@ki.se	Date completed:11/9/2023

1. TRANSLATION

A ---No revisions	☐
B --- Revision(s) suggested	■
C --- Revision(s) required	☐

If "B" or "C," please provide an explanation or example: See my questions above

Does the search strategy match the research question/PICO?
Are the search concepts clear?
Are there too many or too few PICO elements included?
Are the search concepts too narrow or too broad? Mental disorders is too broad?
Does the search retrieve too many or too few records? (Please show number of hits per line.)
Are unconventional or complex strategies explained

2. BOOLEAN AND PROXIMITY OPERATORS

A ---No revisions	■
B --- Revision(s) suggested	☐
C --- Revision(s) required	☐

If "B" or "C," please provide an explanation or example:

Are Boolean or proximity operators used correctly?
Is the use of nesting with brackets appropriate and effective for the search?
If NOT is used, is this likely to result in any unintended exclusions?
Could precision be improved by using proximity operators (eg, adjacent, near, within) or phrase searching instead of AND?
Is the width of proximity operators suitable (eg, might adj5 pick up more variants than adj2)?

3. SUBJECT HEADINGS

A ---No revisions	■
B --- Revision(s) suggested	☐
C --- Revision(s) required	☐

If "B" or "C," please provide an explanation or example: See my comment above

Are the subject headings relevant?
Are any subject headings too broad or too narrow?

Awortwe V, *et al. BMJ Open* 2024; 14:e081927. doi: 10.1136/bmjopen-2023-081927

Are subject headings exploded where necessary and vice versa?<br>Are major headings ("starring" or restrict to focus) used? If so, is there adequate justification?<br>Are subheadings missing?<br>Are subheadings attached to subject headings? (Floating subheadings may be preferred.)<br>Are floating subheadings relevant and used appropriately?<br>Are both subject headings and terms in free text (see the following) used for each concept?

4. TEXT WORD SEARCHING

A ---No revisions	■
B --- Revision(s)suggested	☐
C --- Revision(s) required	☐

If "B" or "C," please provide an explanation or example:

Does the search include all spelling variants in free text (eg, UK vs. US spelling)?<br>Does the search include all synonyms or antonyms (eg, opposites)?<br>Does the search capture relevant truncation (i.e., is truncation at the correct place)?<br>Is the truncation too broad or too narrow?<br>Are acronyms or abbreviations used appropriately? Do they capture irrelevant material? Are the full terms also included?<br>Have the appropriate fields been searched; for example, is the choice of the text word fields (.tw.) or all fields (.af.) appropriate? Are there any other fields to be included or excluded (database specific)?<br>Should any long strings be broken into several shorter search statements?

5. SPELLING, SYNTAX, AND LINE NUMBERS

A ---No revisions	■
B --- Revision(s)suggested	☐
C --- Revision(s) required	☐

If "B" or "C," please provide an explanation or example:

Are there any spelling errors?<br>Are there errors in system syntax (e.g. the use of a truncation symbol from a different search interface)?<br>Are there incorrect line combinations or orphan lines (e.g. lines that are not referred to in the final summation that?<br>could indicate an error in an AND or OR statement)?

6. LIMITS AND FILTERS

A ---No revisions	■
B --- Revision(s) suggested	☐
C --- Revision(s) required	☐

Awortwe V, *et al. BMJ Open* 2024; 14:e081927. doi: 10.1136/bmjopen-2023-081927

If "B" or "C," please provide an explanation or example:

Are all limits and filters used appropriately and are they relevant given the research question?<br>Are all limits and filters used appropriately and are they relevant for the database?<br>Are any potentially helpful limits or filters missing? Are the limits or filters too broad or too narrow? Can any limits<br>or filters be added or removed?<br>Are sources cited for the filters used?

OVERALL EVALUATION (Note: If one or more "revision required" is noted above, the response below must be "revisions required".)

A ---No revisions	■
B --- Revision(s) suggested	☐
C --- Revision(s) required	☐

Additional comments:

Any specific time period for the included studies?

Co-morbidity between depression and anxiety common, how will you treat data on that?

Author response:

Thanks for your feedback. Databases will be searched from inception. If data allows, we will explore sources of heterogeneity via subgroup analysis of the moderating effect of time period of data collection on prevalence (e.g., <2020 vs. ≥ 2020).

We will analyze prevalence estimates of anxiety, depression or a combined estimate of their symptom (i.e. psychological distress) separately. We could consider data on comorbidity between anxiety and depression and this would involve presenting the percentage of adults who experience both conditions simultaneously. Any protocol amendments will be recorded in PROSPERO.
