# Supplementary material for: Prevalence and social determinants of anxiety and depression among adults in Ghana: a systematic review and meta-analysis protocol
Source: BMJ Open. 2024 Apr 23;14(4):e081927. doi: 10.1136/bmjopen-2023-081927 (PMC11043746; doi:10.1136/bmjopen-2023-081927)
Supplement: Supplementary data [file bmjopen-2023-081927supp003.pdf]

Prevalence and social determinants of anxiety and depression among adults in Ghana: a systematic review and meta-analysis protocol

Supplementary Appendix 3: Search strategies-electronic databases

PubMed

#	Searches	Comment
1	Mental health [Title/Abstract] OR<br>Mental disorders [Title/Abstract] OR<br>Mental disorder [Title/Abstract] OR<br>Mental illness [Title/Abstract] OR<br>Mental illnesses [Title/Abstract] OR<br>Anxiety [Title/Abstract] OR<br>Anxious [Title/Abstract] OR<br>Depress*[Title/Abstract] OR<br>Dysthymia [Title/Abstract] OR<br>Melancholy [Title/Abstract] OR<br>Mood [Title/Abstract] OR<br>Affective disorder [Title/Abstract] OR<br>Affective symptoms [Title/Abstract] OR<br>Negative affect [Title/Abstract] OR<br>distress [Title/Abstract] OR<br>Emotional stress [Title/Abstract] OR	
2	Mental health [MeSH] OR<br>Mental disorders [MeSH] OR<br>Anxiety [MeSH] OR<br>Anxiety disorders [MeSH] OR<br>Depression [MeSH] OR<br>Mood disorders [MeSH] OR<br>Psychological Distress [MeSH] OR	
3	1 OR 2	
Ghana		
4	Ghana [All fields]	
5	Ghana [MeSH]	
6	4 OR 5	
Final results	3 AND 6	
syntax	((((((((((((((((Mental health[Title/Abstract]) OR (Mental disorders[Title/Abstract])) OR (mental disorder[Title/Abstract])) OR (mental illness[Title/Abstract])) OR (mental illnesses[Title/Abstract])) OR (anxiety[Title/Abstract])) OR (anxious[Title/Abstract])) OR (depress*[Title/Abstract])) OR (dysthymia[Title/Abstract])) OR	

	(melancholy[Title/Abstract])) OR (mood[Title/Abstract])) OR (affective disorder[Title/Abstract])) OR (affective symptoms[Title/Abstract])) OR (negative affect[Title/Abstract])) OR (distress[Title/Abstract])) OR (emotional stress[Title/Abstract])) OR ((((((((mental health[MeSH Terms]) OR (mental disorders[MeSH Terms])) OR (anxiety[MeSH Terms])) OR (anxiety disorders[MeSH Terms])) OR (depression[MeSH Terms])) OR (depressive disorder[MeSH Terms])) OR (mood disorders[MeSH Terms])) OR (psychological distress[MeSH Terms]))) AND ((Ghana) OR (Ghana[MeSH Terms]))

CINAHL

#	Searches	Comment
1	MW= (mental health OR mental disorders OR anxiety OR anxiety disorders OR depression OR mood disorders OR psychological Distress)	
2	TI= (mental health OR mental disorder*OR mental disorder OR mental illness OR mental illnesses OR anxiety OR anxious OR depress* OR dysthymia OR melancholy OR mood OR affective disorder OR affective symptoms OR negative affect OR distress OR emotional stress).	
3	AB= (mental health OR mental disorder*OR mental disorder OR mental illness OR mental illnesses OR anxiety OR anxious OR depress* OR dysthymia OR melancholy OR mood OR affective disorder OR affective symptoms OR negative affect OR distress OR emotional stress)	In Cinahl Title and Abstract are divided. Search 3 is for abstract OR between terms.
4	1 OR 2 OR 3	
5	MW=(Ghana)	Same as concept 1
6	TI=(Ghana)	
7	AB= (Ghana)	
8	5 OR 6 OR 7	
9 Final results	4 AND 8	
syntax	((MW (Mental health OR mental disorders OR anxiety OR anxiety disorders OR depression OR mood disorders OR psychological Distress) OR TI (Mental health OR mental disorder*OR mental disorder OR mental illness OR mental illnesses OR anxiety OR anxious OR depress* OR dysthymia OR melancholy OR mood OR affective disorder OR affective symptoms OR negative affect OR distress OR emotional stress) OR AB(Mental health OR mental disorder*OR mental disorder OR mental illness OR mental illnesses OR anxiety OR anxious OR depress* OR dysthymia OR melancholy	

	OR mood OR affective disorder OR affective symptoms OR negative affect OR distress OR emotional stress)) AND (MW(Ghana) OR TI(Ghana) OR AB(Ghana))

Embase

#	Searches	Comment
1	TI, AB= (mental health OR mental disorders OR mental disorder OR mental illness OR mental illnesses OR anxiety OR anxious OR depress* OR dysthymia OR melancholy OR mood OR affective disorder OR affective symptoms OR negative affect OR distress OR emotional stress).	
2	/ DE= (mental health OR mental disorders OR anxiety OR anxiety disorders OR depression OR mood disorders OR psychological distress)	Unexploded includes only a single term in hierarchy.<br>[Emtree unexploded] =/de
3	1 OR 2	
4	“Ghana”/exp	
5	“Ghana”. ti, ab.	
6	4 OR 5	
9 Final results	3 AND 6	

PsycINFO

#	Searches	Comment
1	MA= (mental health OR mental disorders OR anxiety OR anxiety disorders OR depression OR mood disorders OR psychological distress)	
2	TI= (Mental health OR mental disorder OR mental disorder OR mental illness OR mental illnesses OR anxiety OR anxious	

	OR depress* OR dysthymia OR melancholy<br>OR mood OR affective disorder OR affective symptoms OR negative affect OR distress OR emotional stress)	
3	AB= (Mental health OR mental disorder OR mental disorder OR mental illness OR mental illnesses OR anxiety OR anxious OR depress* OR dysthymia OR melancholy<br>OR mood OR affective disorder OR affective symptoms OR negative affect OR distress OR emotional stress)	OR between terms.
4	1 OR 2 OR 3	
5	MA= (Ghana)	Same as concept 1
6	TI= (Ghana)	
7	AB= (Ghana)	
8	5 OR 6 OR 7	
9 Final results	4 AND 8	

Scopus

#	Searches
1	TITLE-ABS-KEY (mental health OR mental disorder*OR mental illness*OR anxiety disorders OR anxiety OR anxious OR dysthymia OR melancholy OR mood OR affective disorder OR affective symptoms OR negative affect OR distress OR emotional stress OR depress*OR mood disorders OR psychological Distress)
2	TITLE-ABS-KEY(Ghana)
3 Final results	1 AND 2

African Index Medicus (AIM)

#	Searches	Comment
1	TITLE-ABS-SUB (mental health OR mental disorders OR mental disorder OR mental illness OR mental illnesses OR anxiety OR anxious OR depress* OR dysthymia OR melancholy OR mood OR affective disorder OR affective symptoms OR negative affect OR distress OR emotional stress)	Due to no Search history a simplified strategy was employed: In one go, using title, abstract, subject field.
2	TITLE-ABS-SUB (Ghana)	
3 Final results	1 AND 2	

African journals online (AJOL)

#	Searches	Comment
1	Ghana AND ("mental disorders" OR "mental health" OR "anxiety" OR "anxiety disorders" OR "depression" OR "depressive symptoms" OR "psychological distress" OR “mental distress”) site:ajol.info	AJOL has a google search engine. We used Google scholar interface, incognito mode to minimize effect of Google algorithm. The search yields the same results without incognito mode. Cut-off at 5(50 studies), due to fluctuating result from page 6 onwards.

Health Science Investigation (HIS)

#	Searches	Comment
1	(mental disorders OR mental health OR anxiety OR anxiety disorders OR depression OR depressive symptoms OR psychological distress OR mental distress) AND Ghana	HIS has two advanced filters: Published After (2020,2021,2022 or 2023) and Published before (2020, 2021,2022 or 2023). The search yielded no result when no filter was applied. However,

		when, for instance, 2020 was selected for published after and 2023 for published before, the search yielded results. Note: The search did not yield any results when the inverted commas were added to the searches.

Ghana Medical Journal (GMJ)

#	Searches	Comment
1	(mental disorders OR mental health OR anxiety OR anxiety disorders OR depression OR depressive symptoms OR psychological distress OR mental distress) AND Ghana site:ghanamedj.org	GMJ is indexed in AJOL and since both sites use google interface, we searched in Google scholar interface, incognito mode to minimize effect of Google algorithm. The search yields the same results without incognito mode.
