# Supplementary material for: Prevalence and social determinants of anxiety and depression among adults in Ghana: a systematic review and meta-analysis protocol
Source: BMJ Open. 2024 Apr 23;14(4):e081927. doi: 10.1136/bmjopen-2023-081927 (PMC11043746; doi:10.1136/bmjopen-2023-081927)
Supplement: Supplementary data [file bmjopen-2023-081927supp004.pdf]

Prevalence and social determinants of anxiety and depression among adults in Ghana: a systematic review and meta-analysis protocol

Supplementary Appendix 4: Data extraction form

Study Identification Features	
Study ID/ record number	
Title and aim	
First author last name	
Year of publication	
Publication type or Source of data (e.g., journal or report)	
Study Characteristics	
Age (M, SD) and range	
Gender	
Socioeconomic status	
Chronic physical condition reported (e.g., cancer, diabetes, HIV/AIDs)	
Sample size	
Sampling methods (e.g., convenience sampling, random sampling)	
Geographic region	
Geographical location (rural, urban mixed)	
Recruitment setting (Community or clinical settings	
Study design (e.g., cohort study, cross-sectional study)	
Time period of data collection	
Type of mental health condition(s) assessed (i.e. anxiety, depression, and psychological distress)	
Structured diagnostic clinical interview	Yes/No
Name of diagnostic clinical interview	
Screening tool	Yes/No
Name of screening tool and cutoff scores	
Evidence of validity of the measurement instrument	
Evidence of ethical approval	
Result Summary	
Results Summary Prevalence[a]	
Binary prevalence data (n/N) and percentage with 95% confidence intervals	
Prevalence type (current, period or period or life time)	
Social determinants summary	

Domains of the Social determinants	Proximal factors identified		Distal factors identified	
	Risk factors	Protective factors	Risk factors	Protective factors
Demographic domain				
Economic domain				
Environmental domain				
Neighbourhood domain				
Social and cultural domain				
Reviewer comments, if any?				

[a]Prevalence data will be extracted for all conditions, namely, depression, anxiety and psychological distress
