# Supplementary material for: Prevalence and social determinants of anxiety and depression among adults in Ghana: a systematic review and meta-analysis protocol
Source: BMJ Open. 2024 Apr 23;14(4):e081927. doi: 10.1136/bmjopen-2023-081927 (PMC11043746; doi:10.1136/bmjopen-2023-081927)
Supplement: Author's manuscript [file bmjopen-2023-081927.draft_revisions.pdf]

BMJ Open is committed to open peer review. As part of this commitment we make the peer review history of every article we publish publicly available.

When an article is published we post the peer reviewers' comments and the authors' responses online. We also post the versions of the paper that were used during peer review. These are the versions that the peer review comments apply to.

The versions of the paper that follow are the versions that were submitted during the peer review process. They are not the versions of record or the final published versions. They should not be cited or distributed as the published version of this manuscript.

BMJ Open is an open access journal and the full, final, typeset and author-corrected version of record of the manuscript is available on our site with no access controls, subscription charges or pay-per-view fees (http://bmjopen.bmj.com).

If you have any questions on BMJ Open's open peer review process please email info.bmjopen@bmj.com

BMJ Open

Prevalence and social determinants of anxiety and depression among adults in Ghana: a systematic review and meta-analysis protocol

Journal:	*BMJ Open*
Manuscript ID	bmjopen-2023-081927
Article Type:	Protocol
Date Submitted by the Author:	09-Nov-2023

Complete List of Authors:	Awortwe, Victoria; Uppsala University, Department of Women’s and Children’s Health<br>Daivadanam, Meena ; Karolinska Institutet, Department of Global Public Health; Uppsala University, International Child Health and Nutrition Department of Women's and Children's Health<br>Adjorlolo, Samuel ; College of Health Sciences University of Ghana, Department of Mental Health<br>Olsson, Erik; Uppsala University, Department of Women’s and Children’s Health<br>Coumoundouros, Chelsea; Uppsala University, Department of Women’s and Children’s Health<br>Woodford, Joanne; Uppsala University, Department of Women’s and Children’s Health

Keywords:	Adult psychiatry < PSYCHIATRY, EPIDEMIOLOGY, MENTAL HEALTH, PUBLIC HEALTH, Systematic Review

SCHOLARONE™
Manuscripts

Prevalence and social determinants of anxiety and depression among adults in Ghana: a systematic review and meta-analysis protocol

Victoria AWORTWE[1] (victoria.awortwe@uu.se), Meena DAIVADANAM[2,3,4] (meena.daivadanam@kbh.uu.se), Samuel ADJORLOLO[5] (sadjorlolo@ug.edu.gh), Erik OLSSON[1] (erik.olsson@kbh.uu.se), Chelsea COUMOUNDOUROS[1]* (chelsea.coumoundouros@kbh.uu.se), Joanne WOODFORD[1]* (joanne.woodford@kbh.uu.se)

*Joint last authorship

[1]Healthcare Sciences and e-Health, Department of Women's and Children's Health, Uppsala University, Uppsala, Sweden.

[2]Department of Global Public Health, Karolinska Institutet, Stockholm, Sweden.

[3]Department of Food Studies, Nutrition and Dietetics, Uppsala University, Uppsala, Sweden.

[4]International Child Health and Nutrition, Department of Women's and Children's Health, Uppsala University, Uppsala, Sweden.

[5]Department of Mental Health, School of Nursing and Midwifery, College of Health Sciences, University of Ghana, Accra, Ghana.

Corresponding author

Victoria Awortwe

For peer review only - http://bmjopen.bmj.com/site/about/guidelines.xhtml

ABSTRACT

Introduction: Anxiety and depression pose a significant global health challenge, especially affecting adults in low-and middle-income countries. In many low and middle-income countries, including those in sub-Saharan Africa, social determinants such as access to affordable health services, conflict, food insecurity, and poverty influence the prevalence of anxiety and depression, further contributing to health disparities. To mitigate the burden of anxiety and depression in sub-Saharan Africa, it is essential to develop country-level tailored mental health policies and strategies. For example, Ghana is working towards improving mental health via its 12-year Mental Health policy launched in 2021. However, the prevalence of anxiety and depression among adults in Ghana, along with associated social determinants remains largely unknown, posing challenges for mental health planning, resource allocation, and developing targeted interventions. This review aims to determine the prevalence of anxiety and depression among adults in Ghana, and explore social determinants potentially associated with anxiety and depression.

Methods and analysis: Electronic databases (e.g., African Index Medicus, CINAHL, EMBASE, MEDLINE, PsycINFO) will be searched with all screening steps conducted by two independent reviewers. Secondary search strategies, including grey literature searches, will be used. Studies reporting on the prevalence of anxiety, depression, and/or a combined symptom measure (i.e. psychological distress) among adults in Ghana, using validated instruments will be included. If data allows, random-effects-meta-analyses will be performed to estimate pooled prevalence rates of anxiety and depression. Potential clinical and methodological moderators will be examined using subgroup analyses and meta-regression. A narrative synthesis will explore social determinants potentially associated with anxiety and depression among adults in Ghana.

Ethics and dissemination: Ethical approval is not required as no primary data will be collected. Results will be disseminated via a peer-reviewed publication and presentations at academic conferences. Plain language summaries will be provided to relevant non-governmental organizations working in Ghana.

PROSPERO registration number: CDR42023463078

For peer review only - http://bmjopen.bmj.com/site/about/guidelines.xhtml

Strengths and limitations of this study

- This review, to our knowledge, is the first of its kind, aiming to determine the prevalence of anxiety and depression among adults in Ghana and explore the social determinants potentially associated with anxiety and depression.
- The review protocol adheres to quality standards informed by the Preferred Reporting Items for Systematic Reviews and Meta-Analysis Protocols (PRISMA-P) checklist, with all screening steps and quality appraisal conducted by two independent reviewers.
- The peer-reviewed, comprehensive search strategy will ensure the inclusion of a wide range of relevant studies, reducing the risk of selection bias.
- High levels of heterogeneity across studies due to differences in populations, condition measurements and study designs may limit the possibility of conducting a meta-analysis.
- Qualitative studies will be excluded therefore limiting an in-depth exploration of the perspectives of adults in Ghana on social determinants associated with their mental health, which may have provided important information to inform future intervention development.

For peer review only

For peer review only - http://bmjopen.bmj.com/site/about/guidelines.xhtml

INTRODUCTON

Common mental disorders, such as anxiety and depression are among the most prevalent and rising health problems affecting adults worldwide.[1 2] Anxiety and depression are leading causes of health-related burden globally, with depression being the second and anxiety being the eighth leading cause of years lived with disability (YLDs) out of 369 diseases and injuries in 204 countries and territories.[1] The consequences of anxiety and depression can be severe; resulting in impaired social functioning, increased mortality rates, low productivity, and reduced quality of life.[3] Research suggests anxiety and depression are associated with an increased risk of developing complications or worsening a range of chronic physical conditions including cancer, diabetes, heart disease, and human immunodeficiency virus (HIV).[4–6] The economic burden of anxiety and depression is also substantial with costs stemming from healthcare service use, lost productivity, and impact on families, caregivers, and wider society. [7 8] The burden and impact of anxiety and depression on individuals and societies underscores the critical need to prioritize adult mental health and well-being globally.

The majority of the global burden of mental disorders is located in low-and middle-income countries (LMICs), [9] where 75% of individuals experiencing common mental disorders do not receive appropriate mental health care.[10] This treatment gap is particularly pronounced in Africa owing to the limited availability of mental health resources, coupled with healthcare systems that are inadequately equipped to meet the needs of individuals seeking care.[11 12] A previous scoping review conducted in 12 African countries found a lifetime prevalence rate of anxiety ranging from 5.7% to 15.8% and depression ranging from 3.3% to 9.8%.[13] Within Africa, the prevalence of mental disorders including anxiety and depression is expected to increase by 130% between 2010 and 2050, potentially leading to 45 million YLDs in sub-Saharan Africa (SSA),[14] with major depression predicted to be the largest contributor to disease burden in SSA.[14] Therefore, researchers, global health entities, and advocacy groups are urging actions to address the growing mental health crisis in SSA,[15] advocating for integrating mental health into primary care, establishing national policies, training paraprofessionals, expanding community healthcare, and collaborating with traditional healers.[14 16] To further inform these actions,[15] there is a need to better understand the determinants of mental health that may contribute to the development and exacerbation of mental disorders in SSA, particularly in resource-constrained countries in the region.

For peer review only - http://bmjopen.bmj.com/site/about/guidelines.xhtml

Determinants of mental health among adults are multifaceted, and can be broadly categorized into biological (e.g., genetics, brain chemistry, and hormonal imbalances), psychological (e.g., cognitive and interpersonal factors), and social (e.g., ethnicity, food security, neighbourhood deprivation).[17] Social determinants can also exacerbate biological and psychological vulnerabilities via mechanisms such as gene-environment interactions, epigenetic modifications, and maladaptive coping.[18–20] Globally, social determinants are recognized for their role in either providing protection or increasing the risk of adult mental disorders such as anxiety and depression, while also contributing to health disparities.[21–23] Within LMICs, including those in Africa, social determinants such as including diminished social capital, environmental events, food insecurity, forced migration, income inequality, violence, low education, poor housing, poverty, and unemployment[24–27] may increase vulnerability to experiencing mental health difficulties.[15] Recognizing the importance of social determinants, the World Health Organization (WHO) have urged focus on reshaping economic, physical, and social factors to improve mental health and reduce inequalities by accelerating the implementation of the Comprehensive Mental Health Action Plan 2013-2030.[28] Importantly, the majority of social determinants such as poor living arrangements and low or worsening socioeconomic status are modifiable,[29] and may be improved by implementing appropriate governmental policies and adopting a multi-sectoral approach to provide comprehensive mental health and social care services.[7 30] In SSA, there is a growing consensus that mental health policies and services should be tailored to the contextual realities of each country in the region, with evidence emphasizing the importance of documenting the social determinants specific to each SSA country where mental disorders are experienced. [15,31]

The focus of the present review is Ghana, a LMIC in SSA with a population of approximately 31 million, with an estimated 13% of adults experiencing mental disorders including anxiety and depression.[32] Despite this, only 2% receive treatment.[33] Chronic physical conditions like cancer, diabetes, and HIV are commonly linked to mental disorders among adults in Ghana [34 35] and the burden of anxiety and depression in Ghana is further worsened by inadequate mental health resources and healthcare disparities.[35] Social determinants such as crime, food insecurity, poor sanitation, poverty and unemployment may also contribute to elevated rates of mental disorders in adults in Ghana.[35–38]. To our knowledge, there has been no systematic review undertaken to consolidate existing research, determine the prevalence rates of anxiety and depression among adults; and explore key social determinants specific to the Ghanaian context. By determining the prevalence of anxiety and depression among adults in Ghana and

For peer review only - http://bmjopen.bmj.com/site/about/guidelines.xhtml

exploring social determinants potentially associated with anxiety and depression, this review will provide insights into the magnitude of the problem in Ghana, and social determinants influencing their occurrence to inform evidence-based policies and interventions aimed at enhancing mental well-being and overall health of adults in Ghana. The review's findings will also be valuable for countries experiencing similar social determinants as the Ghanaian population.

Research Objectives

This systematic review seeks to: (1) determine the prevalence of anxiety and depression among adults in Ghana and (2) explore the social determinants potentially associated with anxiety and depression.

METHODS

This review protocol adheres to the Preferred Reporting Items for Systematic Reviews and Meta-Analysis Protocols (PRISMA-P) checklist[39] (online supplementary appendix 1). The Joanna Briggs Institute (JBI) methodology for systematic reviews on prevalence was used to guide protocol development.[40] The protocol has been registered in the International Prospective Register of Systematic Reviews (PROSPERO, registration number CRD42023463078). Any protocol amendments will be recorded in PROSPERO.

Eligibility criteria

Eligibility criteria was developed and defined in accordance with the CoCoPop (Condition, Context, and Population) and type of studies framework.[41]

Population

Adults (aged ≥ 18 years) living in Ghana will be eligible for inclusion. Studies solely focused on children/adolescents will be excluded. Studies focusing on both adults and adolescents will be excluded if they do not present data separately for adults and adolescents.

Condition

Conditions eligible for inclusion include anxiety and / or depression assessed using a structured diagnostic clinical interview in accordance with the International Classification of Diseases

For peer review only - http://bmjopen.bmj.com/site/about/guidelines.xhtml

and Related Health Problems (ICD-10 or ICD-11), or the Diagnostic and Statistical Manual of Mental disorders fifth edition (DSM-V) such as the Structured Clinical Interview for (SCID), the Mini-International Neuropsychiatric Interview (MINI), or the Composite International Diagnostic Interview (CIDI). Studies using a self-report, clinician or proxy administered screening tool for anxiety (e.g., Beck Anxiety Inventory), depression (e.g., Beck Depression Inventory), or psychological distress (e.g., Kessler Psychological Distress Scale) will also be eligible for inclusion. Studies reporting point (current), period (timepoint), or lifetime estimates of the prevalence of anxiety; depression and psychological distress will be included.[42] To ensure the reliability and quality of assessment methods, only studies using validated instruments will be included. Studies solely focused on other mental disorders (e.g., psychotic disorders, bipolar affective disorders), substance use and neurological disorders (e.g., alcohol dependence, multiple sclerosis) will be excluded.

Context

This review will include studies conducted in Ghana with adults sampled from the community or clinical settings (e.g., primary health care facilities, hospitals). Studies conducted in regions that encompass Ghana (e.g., West Africa and Sub-Saharan Africa) will be considered eligible if data on participants living in Ghana can be extracted from the publication or obtained via correspondence with study authors. Studies conducted immediately after conflict, humanitarian crises, or natural disaster will be excluded given we seek to understand the general prevalence of anxiety and depression.[43]

Types of studies

Primary quantitative studies, with observational study designs including longitudinal cohort studies (baseline data only), case-control, and cross-sectional studies. Mixed method studies will be eligible for inclusion only if data from the quantitative component can be clearly extracted.

Information sources

Searches will be conducted in accordance with PRISMA 2020 guidelines.[44] Electronic database searches will be carried out in African Index Medicus (AIM), African Journals Online (AJOL), Cumulative Index to Nursing and Allied Health Literature (CINAHL), Excerpta Medica Database (Embase), Ghana Medical Journal (GMJ), Health Sciences Investigation (HIS),

For peer review only - http://bmjopen.bmj.com/site/about/guidelines.xhtml

For peer review only

MEDLINE (PubMed), PsycINFO and SCOPUS. Databases will be searched from inception. Reference lists of included studies will be manually checked and forward citation checks of included studies will be performed. Reference lists of relevant systematic reviews conducted in sub-Saharan Africa, including Ghana will also be manually checked. Grey literature will be searched in Agency for Healthcare Research and Quality (AHRQ), Google Scholar, Health Systems Trust, Open Grey (http://www. opengrey.eu/) and the World Health Organization (WHO). Researchers and non-governmental organizations working in the area of adult mental health in Ghana will be contacted to identify unpublished or ongoing studies.

For peer review only

Search strategy

The search strategy has been developed in collaboration with Mattias Axén, a librarian at Uppsala University Library and was reviewed by Alkistis Skalkidou and Lene Lindberg, following the PRESS Peer Review guidelines[45] (online supplementary appendix 2). The search was constructed using terms related to (1) mental disorders and (2) Ghana (see online supplementary appendix 3). Electronic databases will be searched using Medical Subject Headings (MeSH) when possible and free text words in title and abstract word searches. No date restriction will be imposed and only studies published in English and Ghanaian languages (e.g., Fante, Ga, Twi) will be considered for inclusion.

Study selection

Studies retrieved from searches will be uploaded into Endnote 20 with duplicates identified and removed. Two reviewers will independently screen titles and abstracts in Rayyan,[46] followed by full paper checks of potentially eligible studies. Studies not meeting the eligibility criteria will be excluded. Overall reasons for exclusion will be documented and reported using the PRISMA flow chart and detailed reasons for exclusion, in accordance with the eligibility criteria, will be presented in a table. If study data/information required to determine eligibility is missing, authors will be contacted via email for additional information. Any disagreement between reviewers will be resolved by discussion and/ or involvement of a third reviewer.

For peer review only - http://bmjopen.bmj.com/site/about/guidelines.xhtml

Data extraction

Data from included studies (see online supplementary appendix 4), will be extracted by one reviewer independently into a standardised Microsoft Excel data extraction form, and crosschecked by a second reviewer for accuracy. The following data will be extracted:

1. Study identification features: study ID/record number, study title and aim, first author name, year of publication, and publication type or data source (e.g., journal, or report).
2. Study characteristics: characteristics of study population (age, gender, socioeconomic status, chronic physical condition reported), sample size, sampling methods (e.g., convenience sampling, random sampling), study setting including geographical region(s) in Ghana where study was conducted, location (rural, urban, mixed), and recruitment setting (community or clinical settings), study design (e.g., case-control, cross-sectional, longitudinal cohort studies), time period of data collection, type of mental health condition (anxiety, depression and psychological distress), structured diagnostic clinical interview (yes/no), name of diagnostic clinical interview, screening tool (yes/no), type of screening tool (self- report, clinician or proxy administered), name of screening tool, prevalence, and prevalence type (point, period, lifetime prevalence).
3. Results summary: sample size, binary prevalence data format (number of cases/ sample size, i.e. n/N) or percentage of the proportion of sample with either current, period or life time prevalence of anxiety, depression and psychological distress with 95% confidence intervals, and summary of social determinants classified into demographic, economic, environmental, neighbourhood and, social and cultural domains.

Quality assessment

The methodological quality of all included studies will be independently assessed by two reviewers using the standardized JBI Critical Appraisal Tool for Prevalence Studies.[47] The JBI Critical Appraisal Tool for Prevalence Studies comprises nine items including: appropriateness of sampling frame and sampling technique, adequacy of sample size, coverage of identified sample, description of study setting and subjects, validity of condition identification methods, standard and reliable measurement of condition, statistical analysis, and adequacy of response rate to assess the methodological quality and/or risk of bias of sampling, analysis and measurements in primary studies. The total score ranges from 1 to 9 for individual studies,[48] with the total number of "yes" scores for individual studies averaged to appraise studies as low

For peer review only

(≤ 3 score), moderate (4-6 points) or high quality (≥ 7 points).[49] Any disagreement between reviewers will be resolved by discussion and/ or involvement of a third reviewer.

Data analysis and synthesis

Quantitative data synthesis

If data allows, a meta-analysis with prevalence data from eligible studies will be conducted using Comprehensive Meta-Analysis software.[50] Data on the proportion of adults with anxiety, depression and psychological distress and their respective sample size will be extracted separately from individual studies to generate pooled estimates with exact binomial test and associated 95% confidence intervals. Data will be transformed to their logits before meta-analysis to stabilize variances.[51] Due to expected heterogeneity in individual studies, the random-effect model will be used to generate pooled prevalence estimates for anxiety, depression and psychological distress, respectively.[50] Heterogeneity across studies will be estimated using Cochran's Q statistic. The I^2 statistics will be used to measure the proportion of total variability due to between-study heterogeneity. The prediction interval (T^2) will be used as an estimate of between-study variance in true effects observed in eligible studies. Sensitivity analyses will be performed to explore the impact of individual studies on the overall prevalence estimate of anxiety and depression. This will be conducted for example, by removing studies of lower quality individually from the overall analysis to ascertain if their removal causes any substantial change to overall prevalence estimates. Egger's regression statistic and funnel plots will be used to assess the presence of any publication bias. In the case of significant publication bias,[52] the trim & fill method will be used to identify and correct the asymmetry of the funnel plot to yield a corrected pooled prevalence.

Subgroup analysis

If data allows, we will explore sources of heterogeneity via subgroup analysis of the moderating effects of the following factors on prevalence:

- Chronic physical conditions (e.g., cancer, diabetes, HIV/AIDs).
- Method of mental health assessment (e.g., structured clinical interview or screening tool)

For peer review only - http://bmjopen.bmj.com/site/about/guidelines.xhtml

- Sample size (<100 vs. ≥100)
- Study design (e.g., case-control study, cross-sectional studies, longitudinal cohort study)
- Study quality (low, medium or high-quality studies).
- Prevalence type (point, period, lifetime).
- Time period of data collection (e.g., <2013 vs. ≥2013 or <2020 vs. ≥2020).

Narrative synthesis

Social determinants will be narratively synthesised, with specific distal and/or proximal factors grouped under domains of the social determinants of mental disorders and Sustainable Development Goals (SDGs) framework,[22] outlined below:

1. Demographics: proximal factors (e.g., age, ethnicity, gender) and distal factors (e.g., community diversity, population density).

2. Economic: proximal factors (e.g., assets, debt, unemployment) and distal factors (e.g., economic inequality, recessions).

3. Neighbourhood: proximal factors (e.g., safety and security, housing structure, overcrowding) and distal factors (e.g., neighbourhood deprivation, built environment).

4. Environmental events: proximal factors (e.g., trauma) and distal factors (climate change, forced migration, war or conflict).

5. Social and cultural: proximal factors (e.g., individual social capital, social participation, education) and distal factors (e.g., social stability, community social capital).

Patient and public involvement

We did not involve patient and public contributors in the design and development of this protocol as the review primarily entails data synthesis from existing literature.

For peer review only - http://bmjopen.bmj.com/site/about/guidelines.xhtml

DISCUSSION

To the best of our knowledge, there is currently no comprehensive review examining the prevalence and social determinants of anxiety and depression among adults in Ghana. This review will extend existing epidemiology literature on mental health[36 53 54] by providing a comprehensive summary of prevalence estimates of anxiety and depression among adults in Ghana, as well as social determinants potentially influencing their occurrence. The review has a number of strengths including a peer-reviewed, comprehensive search strategy ensuring the inclusion of a wide range of relevant studies, and reducing the risk of selection bias. By considering comorbidities, the review recognizes that mental health issues often co-occur with physical conditions, contributing to a better understanding of the overall health and mental well-being of adults in Ghana. The review protocol also adheres to quality standards informed by the PRISMA-P checklist,[39] with screening, selection, and quality appraisal assessed by two independent reviewers.

While there are several strengths, there are also limitations. Qualitative studies will be excluded, limiting an in-depth exploration of the perspectives of adults in Ghana on social determinants associated with their mental health, which may have provided important information to inform mental health intervention development. High levels of clinical and methodological heterogeneity across studies due to factors such as differences in populations, condition measurements and study design may limit the possibility of conducting a meta-analysis. This situation may warrant the need for narrative synthesis.

Despite these limitations, the significance of this review is highlighted by the widespread global attention on mental health, with initiatives such as the Comprehensive Mental Health Action Plan 2013-2030 and the United Nation's Sustainable Developmental Goals (SDGs) highlighting the importance of mental health care equity, prevention, treatment, and promotion.[55] Whilst Ghana is working towards improving mental health via its revised 12-year Mental Health Policy launched in 2021,[35] a comprehensive review examining the prevalence of anxiety and depression among adults in Ghana can facilitate the planning and allocation of resources for mental health care.[35] Addressing the social determinants is also crucial for achieving broader development targets, such as SDG 1 for no poverty, SDG 5 for gender equality, and SDG 4 for quality education, among others.[22] Findings may be used to inform future research, mental health-care planning, and the development of culturally responsive interventions aimed at improving the mental well-being and overall health of adults in Ghana.

For peer review only - http://bmjopen.bmj.com/site/about/guidelines.xhtml

ETHICS AND DISSEMINATION

Findings will be disseminated through scientific publication in a peer-reviewed journal and as presentations at conferences. The authors will use scientific presentations at institutional events, regional and international conferences and social media to promote the review result. Plain language summaries will be provided to non-governmental organizations working in the area of adult mental health in Ghana.

Acknowledgments Alkistis Skalkidou (AS) from the Department of Women's and Children's Health at Uppsala University and Lene Lindberg (LL) from the Department of Public Health Sciences at Karolinska Institute for providing peer review of the search strategy. Mattias Axén (MA), a Librarian at Uppsala University Library for assisting with the development of the electronic search strategy. Febrina Maharani (FM), master student in Global Health at Uppsala University for assisting with study selection process.

Contributors: VA, MD and JW conceptualized the study. VA drafted the proposal. VA, CC and JW designed the study. All authors (VA, MD, EO, SA, CC and JW) assisted with manuscript writing and critical revision of the study design and manuscript. All authors read and approved the final manuscript. JW is the guarantor of the review.

Funding This work was supported by U-CARE, which is a strategic research environment funded by the Swedish Research Council (dnr 2009–1093).

Competing interests None declared.

Patient consent for publication Not required.

Ethics approval This study protocol is for a systematic review, meta- analysis and meta synthesis using secondary data. No raw individual level data is included within this review, with only group level data extracted and analysed, as such no formal ethical approval is required.

Provenance and peer review Not commissioned; externally peer reviewed.

For peer review only - http://bmjopen.bmj.com/site/about/guidelines.xhtml

References

1. GBD 2019 Mental Disorders Collaborators. Global, regional, and national burden of 12 mental disorders in 204 countries and territories, 1990-2019: a systematic analysis for the Global Burden of Disease Study 2019. Lancet Psychiatry. 2022 Feb;9(2):137–50.

2. Hyman S, Parikh R, Collins PY, Patel V. Adult Mental Disorders. In: Mental, Neurological, and Substance Use Disorders: Disease Control Priorities, Third Edition (Volume 4) [Internet]. The International Bank for Reconstruction and Development / The World Bank; 2016 [cited 2023 Apr 28]. Available from: https://www.ncbi.nlm.nih.gov/books/NBK361952/

3. Ormel J, VonKorff M. Reducing Common Mental Disorder Prevalence in Populations. JAMA Psychiatry. 2021 Apr 1;78(4):359–60.

4. Daré LO, Bruand PE, Gérard D, Marin B, Lameyre V, Boumédiène F, et al. Co-morbidities of mental disorders and chronic physical diseases in developing and emerging countries: a meta-analysis. BMC Public Health. 2019 Mar 13;19(1):304.

5. Scott KM, Lim C, Al-Hamzawi A, Alonso J, Bruffaerts R, Caldas-de-Almeida JM, et al. Association of Mental Disorders with Subsequent Chronic Physical Conditions: World Mental Health Surveys From 17 Countries. JAMA Psychiatry. 2016 Feb;73(2):150–8.

6. World Health Organisation. Comprehensive Mental Health Action Plan 2013-2030 [Internet]. 2021 [cited 2022 Dec 28]. Available from: https://www.who.int/publications-detail-redirect/9789240031029

7. World Health Organization. Comprehensive Mental Health Action Plan 2013-2030 [Internet]. Geneva: World Health Organization; 2021 [cited 2023 May 29]. Available from: https://www.who.int/publications-detail-redirect/9789240031029

8. World Health Organization and Calouste Gulbenkian Foundation. Social determinants of mental health [Internet]. World Health Organization, Geneva; 2014 [cited 2023 Apr 28]. Available from: https://www.who.int/publications-detail-redirect/9789241506809

9. World Health Organization. Depression and Other Common Mental Disorders: Global Health Estimates [Internet]. Geneva; 2017 [cited 2023 Mar 15]. Available from: https://www.who.int/publications-detail-redirect/depression-global-health-estimates

10. Dua T, Barbui C, Clark N, Fleischmann A, Poznyak V, Ommeren M van, et al. Evidence-Based Guidelines for Mental, Neurological, and Substance Use Disorders in Low- and Middle-Income Countries: Summary of WHO Recommendations. PLOS Medicine. 2011 Nov 15;8(11): e1001122.

11. Lund C, Alem A, Schneider M, Hanlon C, Ahrens J, Bandawe C, et al. Generating evidence to narrow the treatment gap for mental disorders in sub-Saharan Africa: rationale, overview and methods of AFFIRM. Epidemiol Psychiatr Sci. 2015 Jun;24(3):233–40.

12. Sankoh O, Sevalie S, Weston M. Mental health in Africa. The Lancet Global Health. 2018 Sep;6(9): e954–5.

For peer review only - http://bmjopen.bmj.com/site/about/guidelines.xhtml

13. Greene MC, Yangchen T, Lehner T, Sullivan PF, Pato CN, McIntosh A, et al. The epidemiology of psychiatric disorders in Africa: a scoping review. The Lancet Psychiatry. 2021 Aug;8(8):717–31.

14. Charlson FJ, Diminic S, Lund C, Degenhardt L, Whiteford HA. Mental and Substance Use Disorders in Sub-Saharan Africa: Predictions of Epidemiological Changes and Mental Health Workforce Requirements for the Next 40 Years. PLOS ONE. 2014 Oct 13;9(10): e110208.

15. Monteiro N. Addressing mental illness in Africa: Global health challenges and local opportunities. Community Psychology in Global Perspective. 2015 Oct 1; 1:78–95.

16. Atilola O. Cross-cultural child and adolescent psychiatry research in developing countries. Glob Ment Health (Camb). 2015 May 19;2: e5.

17. Patel V, Saxena S, Lund C, Thornicroft G, Baingana F, Bolton P, et al. The Lancet Commission on global mental health and sustainable development. Lancet. 2018 Oct 27;392(10157):1553–98.

18. Compton MT, Shim RS. The Social Determinants of Mental Health. FOC. 2015 Oct;13(4):419–25.

19. Gottschalk MG, Domschke K. Novel developments in genetic and epigenetic mechanisms of anxiety. Curr Opin Psychiatry. 2016 Jan;29(1):32–8.

20. Remes O, Mendes JF, Templeton P. Biological, Psychological, and Social Determinants of Depression: A Review of Recent Literature. Brain Sci. 2021 Dec 10;11(12):1633.

21. Alegría M, NeMoyer A, Falgàs Bagué I, Wang Y, Alvarez K. Social Determinants of Mental Health: Where We Are and Where We Need to Go. Curr Psychiatry Rep. 2018 Sep 17;20(11):95.

22. Lund C, Brooke-Sumner C, Baingana F, Baron EC, Breuer E, Chandra P, et al. Social determinants of mental disorders and the Sustainable Development Goals: a systematic review of reviews. Lancet Psychiatry. 2018 Apr;5(4):357–69.

23. Moitra M, Owens S, Hailemariam M, Wilson KS, Mensa-Kwao A, Gonese G, et al. Global Mental Health: Where We Are and Where We Are Going. Curr Psychiatry Rep. 2023 Jul 1;25(7):301–11.

24. Deglon M, Dalvie MA, Abrams A. The impact of extreme weather events on mental health in Africa: A scoping review of the evidence. Science of The Total Environment. 2023 Jul 10; 881:163420.

25. Hawkins C, Bwanika JM, Ibanda M. Socio-economic factors associated with mental health disorders in Fort Portal, western Uganda. S Afr J Psychiatr. 2020 Jul 7; 26:1391.

26. Musisi S, Kinyanda E. Long-Term Impact of War, Civil War, and Persecution in Civilian Populations—Conflict and Post-Traumatic Stress in African Communities. Frontiers in Psychiatry [Internet]. 2020 [cited 2023 Oct 23];11. Available from: https://www.frontiersin.org/articles/10.3389/fpsyt.2020.00020

For peer review only - http://bmjopen.bmj.com/site/about/guidelines.xhtml

27. Robertson LJ. The impact of urbanization on mental health service provision: a Brazil, Russia, India, China, South Africa and Africa focus. Current Opinion in Psychiatry. 2019 May;32(3):224.

28. World Health Organization. World mental health report: Transforming mental health for all [Internet]. Geneva; 2022 [cited 2023 May 8]. Available from: https://www.who.int/publications-detail-redirect/9789240049338

29. Furber G, Leach M, Guy S, Segal L. Developing a broad categorisation scheme to describe risk factors for mental illness, for use in prevention policy and planning. Aust N Z J Psychiatry. 2017 Mar;51(3):230–40.

30. Yu S. Uncovering the hidden impacts of inequality on mental health: a global study. Transl Psychiatry. 2018 May 18;8(1):98.

31. Adhvaryu A, Fenske J, Nyshadham A. Early Life Circumstance and Adult Mental Health. Journal of Political Economy. 2019 Aug 1;127(4):1516–49.

32. Oppong S, Kretchy IA, Imbeah EP, Afrane BA. Managing mental illness in Ghana: the state of commonly prescribed psychotropic medicines. International Journal of Mental Health Systems. 2016 Apr 5;10(1):28.

33. Eaton J, Ohene S. Providing Sustainable Mental Health Care in Ghana: A Demonstration Project. In: Providing Sustainable Mental and Neurological Health Care in Ghana and Kenya: Workshop Summary [Internet]. National Academies Press (US); 2016 [cited 2023 Jun 9]. Available from: https://www.ncbi.nlm.nih.gov/books/NBK350318/

34. de-Graft Aikins A, Koram K. Health and Healthcare in Ghana, 1957-2017. In: The Economy of Ghana: Sixty Years after Independence. Oxford University Press; 2017.

35. Government of Ghana. Mental Health Policy 2019 -2030: Ensuring A Mentally Healthy Population [Internet]. 2018 [cited 2023 May 8]. Available from: https://extranet.who.int/mindbank/item/7134

36. Amu H, Osei E, Kofie P, Owusu R, Bosoka SA, Konlan KD, et al. Prevalence and predictors of depression, anxiety, and stress among adults in Ghana: A community-based cross-sectional study. PLoS One. 2021 Oct 8;16(10): e0258105.

37. Atuoye KN, Luginaah I. Food as a social determinant of mental health among household heads in the Upper West Region of Ghana. Social Science & Medicine. 2017 May 1; 180:170–80.

38. Greif MJ, Nii-Amoo Dodoo F. How community physical, structural, and social stressors relate to mental health in the urban slums of Accra, Ghana. Health Place. 2015 May; 33:57–66.

39. Moher D, Shamseer L, Clarke M, Ghersi D, Liberati A, Petticrew M, et al. Preferred reporting items for systematic review and meta-analysis protocols (PRISMA-P) 2015 statement. Systematic Reviews. 2015 Jan 1;4(1):1.

For peer review only

40. Munn Z, Moola S, Lisy K, Riitano D, Tufanaru C. Methodological guidance for systematic reviews of observational epidemiological studies reporting prevalence and cumulative incidence data. JBI Evidence Implementation. 2015 Sep;13(3):147.

41. Moola S, Munn Z, Sears K, Sfetcu R, Currie M, Lisy K, et al. Conducting systematic reviews of association (etiology): The Joanna Briggs Institute's approach. JBI Evidence Implementation. 2015 Sep;13(3):163.

42. Steel Z, Marnane C, Iranpour C, Chey T, Jackson JW, Patel V, et al. The global prevalence of common mental disorders: a systematic review and meta-analysis 1980–2013. International Journal of Epidemiology. 2014 Apr;43(2):476–93.

43. Edwards N, Walker S, Paddick SM, Prina AM, Chinnasamy M, Reddy N, et al. Prevalence of depression and anxiety in older people in low- and middle- income countries in Africa, Asia and South America: A systematic review and meta-analysis. Journal of Affective Disorders. 2023 Mar; 325:656–74.

44. Page MJ, McKenzie JE, Bossuyt PM, Boutron I, Hoffmann TC, Mulrow CD, et al. The PRISMA 2020 statement: an updated guideline for reporting systematic reviews. BMJ. 2021 Mar 29;372: n71.

45. McGowan J, Sampson M, Salzwedel DM, Cogo E, Foerster V, Lefebvre C. PRESS Peer Review of Electronic Search Strategies: 2015 Guideline Statement. J Clin Epidemiol. 2016 Jul; 75:40–6.

46. Ouzzani M, Hammady H, Fedorowicz Z, Elmagarmid A. Rayyan—a web and mobile app for systematic reviews. Systematic Reviews. 2016 Dec 5;5(1):210.

47. Munn Z, Moola S, Riitano D, Lisy K. The development of a critical appraisal tool for use in systematic reviews addressing questions of prevalence. Int J Health Policy Manag. 2014;3(3):123–8.

48. Opio JN, Munn Z, Aromataris E. Prevalence of Mental Disorders in Uganda: a Systematic Review and Meta-Analysis. Psychiatr Q. 2022 Mar;93(1):199–226.

49. Aarah-Bapuah M, Sarpomaa Oppong S, Ohenewaa Yawson A, Dzansi G, Adjorlolo S. Covid-19 and mental health of children and adolescents: A systematic review. Cogent Psychology. 2022 Dec 31;9(1):2111849.

50. Borenstein M. Comprehensive Meta-Analysis Software. In 2022. p. 535–48.

51. Doi SAR, Williams GM. Methods of Clinical Epidemiology. Springer Science & Business Media; 2013. 289 p.

52. Sutton AJ, Song F, Gilbody SM, Abrams KR. Modelling publication bias in meta-analysis: a review. Stat Methods Med Res. 2000 Oct;9(5):421–45.

53. Adu MK, Wallace LJ, Lartey KF, Arthur J, Oteng KF, Dwomoh S, et al. Prevalence and Correlates of Likely Major Depressive Disorder among the Adult Population in Ghana during the COVID-19 Pandemic. International Journal of Environmental Research and Public Health. 2021 Jan;18(13):7106.

For peer review only - http://bmjopen.bmj.com/site/about/guidelines.xhtml

54. Ae-Ngibise KA, Sakyi L, Adwan-Kamara L, Lund C, Weobong B. Prevalence of probable mental, neurological and substance use conditions and case detection at primary healthcare facilities across three districts in Ghana: findings from a cross-sectional health facility survey. BMC Psychiatry [Internet]. 2023;23(1). Available from: https://www.scopus.com/inward/record.uri?eid=2-s2.0-85153429606&doi=10.1186%2fs12888-023-04775-z&partnerID=40&md5=4935fd5b031785d325184de537d7941c

55. Campion J, Javed A, Lund C, Sartorius N, Saxena S, Marmot M, et al. Public mental health: required actions to address implementation failure in the context of COVID-19. Lancet Psychiatry. 2022 Feb;9(2):169–82.

For peer review only

Prevalence and social determinants of anxiety and depression among adults in Ghana: a systematic review and meta-analysis protocol

PRISMA-P (Preferred Reporting Items for Systematic review and Meta-Analysis Protocols) 2015 checklist: recommended items to address in a systematic review protocol*

Section and topic	Item No	Checklist item	Location in text (page number)
**ADMINISTRATIVE INFORMATION**			
Title:			
Identification	1a	Identify the report as a protocol of a systematic review	1
Update	1b	If the protocol is for an update of a previous systematic review, identify as such	N/A
Registration	2	If registered, provide the name of the registry (such as PROSPERO) and registration number	2 & 6
Authors:			
Contact	3a	Provide name, institutional affiliation, e-mail address of all protocol authors; provide physical mailing address of corresponding author	1
Contributions	3b	Describe contributions of protocol authors and identify the guarantor of the review	13
Amendments	4	If the protocol represents an amendment of a previously completed or published protocol, identify as such and list changes; otherwise, state plan for documenting important protocol amendments	6
Support:			
Sources	5a	Indicate sources of financial or other support for the review	13
Sponsor	5b	Provide name for the review funder and/or sponsor	13
Role of sponsor or funder	5c	Describe roles of funder(s), sponsor(s), and/or institution(s), if any, in developing the protocol	13
**INTRODUCTION**			
Rationale	6	Describe the rationale for the review in the context of what is already known	5 & 6 &12
Objectives	7	Provide an explicit statement of the question(s) the review will address with reference to participants, interventions, comparators, and outcomes (PICO)	6
**METHODS**			
Eligibility criteria	8	Specify the study characteristics (such as PICO, study design, setting, time frame) and report characteristics (such as years considered, language, publication status) to be used as criteria for eligibility for the review	6 & 7

For peer review only

Information sources	9	Describe all intended information sources (such as electronic databases, contact with study authors, trial registers or other grey literature sources) with planned dates of coverage	7 & 8
Search strategy	10	Present draft of search strategy to be used for at least one electronic database, including planned limits, such that it could be repeated	8 & supplementary appendix 3
Study records:			
Data management	11a	Describe the mechanism(s) that will be used to manage records and data throughout the review	8
Selection process	11b	State the process that will be used for selecting studies (such as two independent reviewers) through each phase of the review (that is, screening, eligibility and inclusion in meta-analysis)	8
Data collection process	11c	Describe planned method of extracting data from reports (such as piloting forms, done independently, in duplicate), any processes for obtaining and confirming data from investigators	9
Data items	12	List and define all variables for which data will be sought (such as PICO items, funding sources), any pre-planned data assumptions and simplifications	9
Outcomes and prioritization	13	List and define all outcomes for which data will be sought, including prioritization of main and additional outcomes, with rationale	9
Risk of bias in individual studies	14	Describe anticipated methods for assessing risk of bias of individual studies, including whether this will be done at the outcome or study level, or both; state how this information will be used in data synthesis	9 & 10
Data synthesis	15a	Describe criteria under which study data will be quantitatively synthesised	10
	15b	If data are appropriate for quantitative synthesis, describe planned summary measures, methods of handling data and methods of combining data from studies, including any planned exploration of consistency (such as I^2, Kendall's τ)	10
	15c	Describe any proposed additional analyses (such as sensitivity or subgroup analyses, meta-regression)	10 & 11
	15d	If quantitative synthesis is not appropriate, describe the type of summary planned	10 &12
Meta-bias(es)	16	Specify any planned assessment of meta-bias(es) (such as publication bias across studies, selective reporting within studies)	9 &10
Confidence in cumulative evidence	17	Describe how the strength of the body of evidence will be assessed (such as GRADE)	N/A

N/A not applicable

*** It is strongly recommended that this checklist be read in conjunction with the PRISMA-P Explanation and Elaboration (cite when available) for important clarification on the items. Amendments to a review protocol should be tracked and dated. The copyright for PRISMA-P (including checklist) is held by the PRISMA-P Group and is distributed under a Creative Commons Attribution Licence 4.0.**

From: Shamseer L, Moher D, Clarke M, Ghersi D, Liberati A, Petticrew M, Shekelle P, Stewart L, PRISMA-P Group. Preferred reporting items for systematic review and meta-analysis protocols (PRISMA-P) 2015: elaboration and explanation. BMJ. 2015 Jan 2;349(jan02 1):pg7647.

For peer review only

Supplementary appendix 2

PRESS Guideline — Search Submission & Peer Review Assessment

SEARCH SUBMISSION: THIS SECTION TO BE FILLED IN BY THE SEARCHER

Searcher: Victoria Awortwe	Email: victoria.awortwe@uu.se
Date submitted: 31/8/2023	Date requested by:11/9/2023

Systematic Review Title:

Prevalence and social determinants of anxiety and depression among adults in Ghana: a systematic review and meta-analysis protocol

This search strategy is …

X	My PRIMARY (core) database strategy — First time submitting a strategy for search question and database
	My PRIMARY (core) strategy — Follow-up review NOT the first time submitting a strategy for search question and database. If this is a response to peer review, itemize the changes made to the review suggestions
	SECONDARY search strategy— First time submitting a strategy for search question and database
	SECONDARY search strategy — NOT the first time submitting a strategy for search question and database. If this is a response to peer review, itemize the changes made to the review suggestions

Database

(i.e., MEDLINE, CINAHL…): *[mandatory]*

MEDLINE

Interface

(i.e., Ovid, EBSCO…): *[mandatory]*

PubMed

Research Question

(Describe the purpose of the search) *[mandatory]*

This review aims to (1) determine the prevalence of anxiety and depression among adults in Ghana; and (2) explore the social determinants potentially associated with anxiety and depression.

For peer review only - http://bmjopen.bmj.com/site/about/guidelines.xhtml

CoCoPop Format

(Outline the CoCoPop for your question — i.e., Condition, Context, Population, and Study Design — as applicable)

**Population**	Adults (individuals aged ≥ 18 years) living in Ghana
**Condition**	Anxiety (i.e. anxiety disorders, anxiety symptoms), depression (i.e. depression, depressive disorders), and/ or psychological distress.
**Context**	Ghana, with adults sampled from the community or clinical settings (e.g., primary health care facilities, hospitals).
**Study design**	Observational study designs

Inclusion Criteria

(List criteria such as age groups, study designs, etc., to be included) *[optional]*

Population: Adults (aged ≥ 18 years) living in Ghana will be eligible for inclusion

Condition: Anxiety and/or depression assessed using structured diagnostic clinical interview in accordance with the International Classification of Diseases and Related Health Problems (ICD-10 or ICD-11), or the Diagnostic and Statistical Manual of Mental disorders (DSM) fifth edition (DSM-V); OR clinician or proxy administered screening tool for anxiety, depression and/or psychological distress. For the meta-analyses, sstudies reporting on the prevalence of anxiety, depression and/or psychological distress among adults in Ghana, measured using validated instruments will be included.

Context: Studies from Ghana, with adults sampled from the community or clinical settings (e.g., primary health care facilities, hospitals). Studies conducted in regions that encompass Ghana (e.g., West Africa and Sub-Saharan Africa) will be considered eligible if data on participants living in Ghana is available. No date restriction will be imposed and only studies published in English and Ghanaian languages (e.g., Fante, Ga, Twi) will be considered for inclusion.

Study design: Primary quantitative studies, with observational study designs including longitudinal cohort studies, case-control, and cross-sectional studies. Mixed method studies will be eligible for inclusion only if data from the quantitative component can be clearly extracted.

Exclusion Criteria

(List criteria such as study designs, date limits, etc., to be excluded) *[optional]*

Population: Studies solely focused on children/adolescents will be excluded.
Studies focusing on both adults and adolescents will be excluded if they do not present data separately for adults and adolescents.

For peer review only

Condition: Studies solely focused on other mental disorders (e.g., psychotic disorders, bipolar affective disorders), substance use and neurological disorders (e.g., alcohol dependence, multiple sclerosis) will be excluded.

Context: Studies that were conducted immediately after conflict, humanitarian crises, or natural disaster will be excluded.

Study design: Review articles, qualitative studies, letters to the editor or editorial comments, and working papers will be excluded; and studies published in languages other than English and Ghanaian will be excluded.

Was a search filter applied?

Yes ☐ No ■

If YES, which one(s) (e.g., Cochrane RCT filter, PubMed Clinical Queries filter)? Provide the source if this is a published filter. *[mandatory if YES to previous question — textbox]*

Other notes or comments you feel would be useful for the peer reviewer? ***[optional]***

We did not include search terms regarding (1) prevalence, as that is not the only area of interest; and (2) adults, as the term can be referred to in a variety of ways. Furthermore, we searched Ghana in All fields to accommodate studies conducted in regions that encompass Ghana (e.g., West Africa and Sub-Saharan Africa) providing data on participants living in Ghana.

Please copy and paste your search strategy here, exactly as run, including the number of hits per line. ***[mandatory]***

For peer review only

Database: PubMed. Final number of results from the full search 1297	
**1: Mental disorders**	**2: Ghana**
**Title & Abstract**<br>Mental health 228145<br>Mental disorders 51217<br>Mental disorder 11824<br>Mental illness 37257<br>Mental illnesses 6045<br>Anxiety 265049<br>Anxious 20603<br>Depress* 569151<br>Dysthymia 2320<br>Melancholy 426<br>Mood 92155<br>Affective disorder 7725<br>Affective symptoms 2417<br>Negative affect 13573<br>distress 154761<br>Emotional stress 5232<br><br>**MeSH terms**<br>Mental health 61933<br>Mental disorders 1436875<br>Anxiety 112111<br>Anxiety disorders 90630<br>Depression 257186<br>Depressive disorder 122190<br>Mood disorders 170032<br>Psychological Distress 6832	**All fields**<br>Ghana 24845<br><br>**MeSH term**<br>Ghana 11236
**Total for concept 1:**	**Total for concept 2:**
**2183845**	**25013**

Total for complete search: 1297

For peer review only - http://bmjopen.bmj.com/site/about/guidelines.xhtml

For peer review only

Raw PubMed Search

("mental health"[Title/Abstract] OR "mental disorders"[Title/Abstract] OR "mental disorder"[Title/Abstract] OR "mental illness"[Title/Abstract] OR "mental illnesses"[Title/Abstract] OR "anxiety"[Title/Abstract] OR "anxious"[Title/Abstract] OR "depress*"[Title/Abstract] OR "dysthymia"[Title/Abstract] OR "melancholy"[Title/Abstract] OR "mood"[Title/Abstract] OR "affective disorder"[Title/Abstract] OR "affective symptom"[Title/Abstract] OR "negative affect"[Title/Abstract] OR "distress"[Title/Abstract] OR "emotional stress"[Title/Abstract] OR ("mental health"[MeSH Terms] OR "mental disorders"[MeSH Terms] OR "anxiety"[MeSH Terms] OR "anxiety disorders"[MeSH Terms] OR ("depressive disorder"[MeSH Terms] OR "depression"[MeSH Terms]) OR "depressive disorder"[MeSH Terms] OR "mood disorders"[MeSH Terms] OR "psychological distress"[MeSH Terms])) AND ("Ghana"[MeSH Terms] OR "Ghana"[All Fields] OR "Ghana s"[All Fields] OR "Ghana"[MeSH Terms]).

For peer review only

PEER REVIEW ASSESSMENT: THIS SECTION TO BE FILLED IN BY THE REVIEWER

Reviewer: Professor Alkistis Skalkidou	Email: Alkistis.skalkidou@kbh.uu.se	Date completed: 14/9/2023

1. TRANSLATION

A ---No revisions	■
B --- Revision(s) suggested	☐
C --- Revision(s) required	☐

If "B" or "C," please provide an explanation or example:

Does the search strategy match the research question/PICO?
Are the search concepts clear?
Are there too many or too few PICO elements included?
Are the search concepts too narrow or too broad?
Does the search retrieve too many or too few records? (Please show number of hits per line.)
Are unconventional or complex strategies explained

2. BOOLEAN AND PROXIMITY OPERATORS

A ---No revisions	■
B --- Revision(s) suggested	☐
C --- Revision(s) required	☐

If "B" or "C," please provide an explanation or example:

Are Boolean or proximity operators used correctly?
Is the use of nesting with brackets appropriate and effective for the search?
If NOT is used, is this likely to result in any unintended exclusions?
Could precision be improved by using proximity operators (eg, adjacent, near, within) or phrase searching instead of AND?
Is the width of proximity operators suitable (eg, might adj5 pick up more variants than adj2)?
Not used

3. SUBJECT HEADINGS

A ---No revisions	☐
B --- Revision(s) suggested	■
C --- Revision(s) required	☐

If "B" or "C," please provide an explanation or example:

For peer review only

Are the subject headings relevant?
Are any subject headings too broad or too narrow?
Are subject headings exploded where necessary and vice versa?
Are major headings ("starring" or restrict to focus) used? If so, is there adequate justification?
Are subheadings missing?
Are subheadings attached to subject headings? (Floating subheadings may be preferred.)
Are floating subheadings relevant and used appropriately?
Are both subject headings and terms in free text (see the following) used for each concept?

For peer review only

4. TEXT WORD SEARCHING

A ---No revisions	■
B --- Revision(s)suggested	☐
C --- Revision(s) required	☐

If "B" or "C," please provide an explanation or example:

Does the search include all spelling variants in free text (eg, UK vs. US spelling)?
Does the search include all synonyms or antonyms (eg, opposites)?
Does the search capture relevant truncation (i.e., is truncation at the correct place)?
Is the truncation too broad or too narrow?
Are acronyms or abbreviations used appropriately? Do they capture irrelevant material? Are the full terms also included?
Have the appropriate fields been searched; for example, is the choice of the text word fields (.tw.) or all fields (.af.) appropriate? Are there any other fields to be included or excluded (database specific)?
Should any long strings be broken into several shorter search statements?

5. SPELLING, SYNTAX, AND LINE NUMBERS

A ---No revisions	■
B --- Revision(s)suggested	☐
C --- Revision(s) required	☐

If "B" or "C," please provide an explanation or example:

Are there any spelling errors?
Are there errors in system syntax (e.g. the use of a truncation symbol from a different search interface)?
Are there incorrect line combinations or orphan lines (e.g. lines that are not referred to in the final summation that?
could indicate an error in an AND or OR statement)?

6. LIMITS AND FILTERS

A ---No revisions	☐

B --- Revision(s) suggested	■
C --- Revision(s) required	☐

If "B" or "C," please provide an explanation or example:

Are all limits and filters used appropriately and are they relevant given the research question?

Are all limits and filters used appropriately and are they relevant for the database?

Are any potentially helpful limits or filters missing? Are the limits or filters too broad or too narrow? Can any limits

or filters be added or removed? Language? Time period? Only humans?

Are sources cited for the filters used?

Author response:

Thanks for the suggestion. Databases will be searched from inception, and the time period for data collection (e.g., <2020 vs. ≥2020) will be investigated as a methodological moderator of prevalence utilising subgroup analyses and meta-regression. Only human studies and language search filters were not used. However, non-human studies and studies published in languages other than English and Ghanaian would be excluded during the screening phase.

OVERALL EVALUATION (Note: If one or more "revision required" is noted above, the response below must be "revisions required".)

A ---No revisions	☐
B --- Revision(s) suggested	■
C --- Revision(s) required	☐

Additional comments:

I am not familiar with Medline, but many within psychology also search the PSYCHINFO database

Author response:

Thank you for your feedback. MEDLINE (PubMed) is one of the electronic databases in which the searches will be conducted. However, electronic database searches will be also carried out in African Index Medicus (AIM), African Journals Online (AJOL), Cumulative Index to Nursing and Allied Health Literature (CINAHL), Excerpta Medica Database (Embase), Ghana Medical Journal (GMJ), Health Sciences Investigation (HIS), **PsycINFO** and SCOPUS.

For peer review only - http://bmjopen.bmj.com/site/about/guidelines.xhtml

PEER REVIEW ASSESSMENT #2: THIS SECTION TO BE FILLED IN BY THE REVIEWER

Reviewer: Associate Professor Lene Lindberg	Email: lene.lindberg@ki.se	Date completed:11/9/2023

1. TRANSLATION

A ---No revisions	☐
B --- Revision(s) suggested	■
C --- Revision(s) required	☐

If "B" or "C," please provide an explanation or example: See my questions above

Does the search strategy match the research question/PICO?
Are the search concepts clear?
Are there too many or too few PICO elements included?
Are the search concepts too narrow or too broad? Mental disorders is too broad?
Does the search retrieve too many or too few records? (Please show number of hits per line.)
Are unconventional or complex strategies explained

For peer review only

2. BOOLEAN AND PROXIMITY OPERATORS

A ---No revisions	■
B --- Revision(s) suggested	☐
C --- Revision(s) required	☐

If "B" or "C," please provide an explanation or example:

Are Boolean or proximity operators used correctly?
Is the use of nesting with brackets appropriate and effective for the search?
If NOT is used, is this likely to result in any unintended exclusions?
Could precision be improved by using proximity operators (eg, adjacent, near, within) or phrase searching instead of AND?
Is the width of proximity operators suitable (eg, might adj5 pick up more variants than adj2)?

3. SUBJECT HEADINGS

A ---No revisions	■
B --- Revision(s) suggested	☐
C --- Revision(s) required	☐

If "B" or "C," please provide an explanation or example: See my comment above

Are the subject headings relevant?
Are any subject headings too broad or too narrow?

Are subject headings exploded where necessary and vice versa?

Are major headings ("starring" or restrict to focus) used? If so, is there adequate justification?

Are subheadings missing?

Are subheadings attached to subject headings? (Floating subheadings may be preferred.)

Are floating subheadings relevant and used appropriately?

Are both subject headings and terms in free text (see the following) used for each concept?

4. TEXT WORD SEARCHING

A ---No revisions	■
B --- Revision(s)suggested	☐
C --- Revision(s) required	☐

If "B" or "C," please provide an explanation or example:

Does the search include all spelling variants in free text (eg, UK vs. US spelling)?
Does the search include all synonyms or antonyms (eg, opposites)?
Does the search capture relevant truncation (i.e., is truncation at the correct place)?
Is the truncation too broad or too narrow?
Are acronyms or abbreviations used appropriately? Do they capture irrelevant material? Are the full terms also included?
Have the appropriate fields been searched; for example, is the choice of the text word fields (.tw.) or all fields (.af.) appropriate? Are there any other fields to be included or excluded (database specific)?
Should any long strings be broken into several shorter search statements?

5. SPELLING, SYNTAX, AND LINE NUMBERS

A ---No revisions	■
B --- Revision(s)suggested	☐
C --- Revision(s) required	☐

If "B" or "C," please provide an explanation or example:

Are there any spelling errors?
Are there errors in system syntax (e.g. the use of a truncation symbol from a different search interface)?
Are there incorrect line combinations or orphan lines (e.g. lines that are not referred to in the final summation that?
could indicate an error in an AND or OR statement)?

6. LIMITS AND FILTERS

A ---No revisions	■
B --- Revision(s) suggested	☐
C --- Revision(s) required	☐

For peer review only

If "B" or "C," please provide an explanation or example:

Are all limits and filters used appropriately and are they relevant given the research question?<br>Are all limits and filters used appropriately and are they relevant for the database?<br>Are any potentially helpful limits or filters missing? Are the limits or filters too broad or too narrow? Can any limits<br>or filters be added or removed?<br>Are sources cited for the filters used?

OVERALL EVALUATION (Note: If one or more "revision required" is noted above, the response below must be "revisions required".)

A ---No revisions	■
B --- Revision(s) suggested	□
C --- Revision(s) required	□

Additional comments:

Any specific time period for the included studies?

Co-morbidity between depression and anxiety common, how will you treat data on that?

Author response:

Thanks for your feedback. Databases will be searched from inception. If data allows, we will explore sources of heterogeneity via subgroup analysis of the moderating effect of time period of data collection on prevalence (e.g., <2020 vs. ≥ 2020).

We will analyze prevalence estimates of anxiety, depression or a combined estimate of their symptom (i.e. psychological distress) separately. We could consider data on comorbidity between anxiety and depression and this would involve presenting the percentage of adults who experience both conditions simultaneously. Any protocol amendments will be recorded in PROSPERO.

For peer review only

For peer review only - http://bmjopen.bmj.com/site/about/guidelines.xhtml

Prevalence and social determinants of anxiety and depression among adults in Ghana: a systematic review and meta-analysis protocol

Supplementary File 3: Search strategies-electronic databases

PubMed

#	Searches	Comment
1	Mental health [Title/Abstract] OR<br>Mental disorders [Title/Abstract] OR<br>Mental disorder [Title/Abstract] OR<br>Mental illness [Title/Abstract] OR<br>Mental illnesses [Title/Abstract] OR<br>Anxiety [Title/Abstract] OR<br>Anxious [Title/Abstract] OR<br>Depress*[Title/Abstract] OR<br>Dysthymia [Title/Abstract] OR<br>Melancholy [Title/Abstract] OR<br>Mood [Title/Abstract] OR<br>Affective disorder [Title/Abstract] OR<br>Affective symptoms [Title/Abstract] OR<br>Negative affect [Title/Abstract] OR<br>distress [Title/Abstract] OR<br>Emotional stress [Title/Abstract]	
2	Mental health [MeSH] OR<br>Mental disorders [MeSH] OR<br>Anxiety [MeSH] OR<br>Anxiety disorders [MeSH] OR<br>Depression [MeSH] OR<br>Mood disorders [MeSH] OR<br>Psychological Distress [MeSH] OR	
3	1 OR 2	
Ghana		
4	Ghana [All fields]	
5	Ghana [MeSH]	
6	4 OR 5	
Final results	3 AND 6	
syntax	(((((((((((((((Mental health[Title/Abstract]) OR (Mental disorders[Title/Abstract])) OR (mental disorder[Title/Abstract])) OR (mental illness[Title/Abstract])) OR (mental illnesses[Title/Abstract])) OR (anxiety[Title/Abstract])) OR (anxious[Title/Abstract])) OR (depress*[Title/Abstract])) OR (dysthymia[Title/Abstract])) OR	

For peer review only - http://bmjopen.bmj.com/site/about/guidelines.xhtml

	(melancholy[Title/Abstract])) OR (mood[Title/Abstract])) OR (affective disorder[Title/Abstract])) OR (affective symptoms[Title/Abstract])) OR (negative affect[Title/Abstract])) OR (distress[Title/Abstract])) OR (emotional stress[Title/Abstract])) OR ((((((((mental health[MeSH Terms]) OR (mental disorders[MeSH Terms])) OR (anxiety[MeSH Terms])) OR (anxiety disorders[MeSH Terms])) OR (depression[MeSH Terms])) OR (depressive disorder[MeSH Terms])) OR (mood disorders[MeSH Terms])) OR (psychological distress[MeSH Terms]))) AND ((Ghana) OR (Ghana[MeSH Terms]))

CINAHL

#	Searches	Comment
1	MW= (mental health OR mental disorders OR anxiety OR anxiety disorders OR depression OR mood disorders OR psychological Distress)	
2	TI= (mental health OR mental disorder*OR mental disorder OR mental illness OR mental illnesses OR anxiety OR anxious OR depress* OR dysthymia OR melancholy OR mood OR affective disorder OR affective symptoms OR negative affect OR distress OR emotional stress).	
3	AB= (mental health OR mental disorder*OR mental disorder OR mental illness OR mental illnesses OR anxiety OR anxious OR depress* OR dysthymia OR melancholy OR mood OR affective disorder OR affective symptoms OR negative affect OR distress OR emotional stress)	In Cinahl Title and Abstract are divided. Search 3 is for abstract OR between terms.
4	1 OR 2 OR 3	
5	MW=(Ghana)	Same as concept 1
6	TI=(Ghana)	
7	AB= (Ghana)	
8	5 OR 6 OR 7	
9 Final results	4 AND 8	
syntax	((MW (Mental health OR mental disorders OR anxiety OR anxiety disorders OR depression OR mood disorders OR psychological Distress) OR TI (Mental health OR mental disorder*OR mental disorder OR mental illness OR mental illnesses OR anxiety OR anxious OR depress* OR dysthymia OR melancholy OR mood OR affective disorder OR affective symptoms OR negative affect OR distress OR emotional stress) OR AB(Mental health OR mental disorder*OR mental disorder OR mental illness OR mental illnesses OR anxiety OR anxious OR depress* OR dysthymia OR melancholy	

For peer review only

	OR mood OR affective disorder OR affective symptoms OR negative affect OR distress OR emotional stress)) AND (MW(Ghana) OR TI(Ghana) OR AB(Ghana))

Embase

#	searches	Comment
1	TI, AB= (mental health OR mental disorders OR mental disorder OR mental illness OR mental illnesses OR anxiety OR anxious OR depress* OR dysthymia OR melancholy OR mood OR affective disorder OR affective symptoms OR negative affect OR distress OR emotional stress).	
2	/ DE= (mental health OR mental disorders OR anxiety OR anxiety disorders OR depression OR mood disorders OR psychological distress)	Unexploded includes only a single term in hierarchy. [Emtree unexploded] =/de
3	1 OR 2	
4	"Ghana"/exp	
5	"Ghana". ti, ab.	
6	4 OR 5	
9 Final results	3 AND 6	

PsycINFO

#	Searches	Comment
1	MA= (mental health OR mental disorders OR anxiety OR anxiety disorders OR depression OR mood disorders OR psychological distress)	
2	TI= (Mental health OR mental disorder OR mental disorder OR mental illness OR mental illnesses OR anxiety OR anxious	

For peer review only

	OR depress* OR dysthymia OR melancholy OR mood OR affective disorder OR affective symptoms OR negative affect OR distress OR emotional stress)	
3	AB= (Mental health OR mental disorder OR mental disorder OR mental illness OR mental illnesses OR anxiety OR anxious OR depress* OR dysthymia OR melancholy OR mood OR affective disorder OR affective symptoms OR negative affect OR distress OR emotional stress)	OR between terms.
4	1 OR 2 OR 3	
5	MA= (Ghana)	Same as concept 1
6	TI= (Ghana)	
7	AB= (Ghana)	
8	5 OR 6 OR 7	
9 Final results	4 AND 8	

Scopus

#	**Searches**
1	TITLE-ABS-KEY (mental health OR mental disorder*OR mental illness*OR anxiety disorders OR anxiety OR anxious OR dysthymia OR melancholy OR mood OR affective disorder OR affective symptoms OR negative affect OR distress OR emotional stress OR depress*OR mood disorders OR psychological Distress)
2	TITLE-ABS-KEY(Ghana)
3 Final results	1 AND 2

African Index Medicus (AIM)

#	Searches	Comment
1	TITLE-ABS-SUB (mental health OR mental disorders OR mental disorder OR mental illness OR mental illnesses OR anxiety OR anxious OR depress* OR dysthymia OR melancholy OR mood OR affective disorder OR affective symptoms OR negative affect OR distress OR emotional stress)	Due to no Search history a simplified strategy was employed: In one go, using title, abstract, subject field.
2	TITLE-ABS-SUB (Ghana)	
3 Final results	1 AND 2	

African journals online (AJOL)

#	Searches	Comment
1	Ghana AND ("mental disorders" OR "mental health" OR "anxiety" OR "anxiety disorders" OR "depression" OR "depressive symptoms" OR "psychological distress" OR “mental distress”) site:ajol.info	AJOL has a google search engine. We used Google scholar interface, incognito mode to minimize effect of Google algorithm. The search yields the same results without incognito mode. Cut-off at 5(50 studies), due to fluctuating result from page 6 onwards.

Health Science Investigation (HIS)

#	Searches	Comment
1	(mental disorders OR mental health OR anxiety OR anxiety disorders OR depression OR depressive symptoms OR psychological distress OR mental distress) AND Ghana	HIS has two advanced filters: Published After (2020,2021,2022 or 2023) and Published before (2020, 2021,2022 or 2023). The search yielded no result when no filter was applied. However,

For peer review only

		when, for instance, 2020 was selected for published after and 2023 for published before, the search yielded results. Note: The search did not yield any results when the inverted commas when added to the searches.

Ghana Medical Journal (GMJ)

#	Searches	Comment
1	(mental disorders OR mental health OR anxiety OR anxiety disorders OR depression OR depressive symptoms OR psychological distress OR mental distress) AND Ghana site:ghanamedj.org	GMJ is indexed in AJOL and since both sites use google interface, we searched in Google scholar interface, incognito mode to minimize effect of Google algorithm. The search yields the same results without incognito mode.

For peer review only

Prevalence and social determinants of anxiety and depression among adults in Ghana: a systematic review and meta-analysis protocol

Supplementary Appendix 4: Data extraction form

Study Identification Features	
Study ID/ record number	
Title and aim	
First author last name	
Year of publication	
Publication type or Source of data (e.g., journal or report)	
Study Characteristics	
Age (M, SD)	
Gender	
Socioeconomic status	
Chronic physical condition reported (e.g., cancer, diabetes, HIV/AIDs)	
Sample size	
Sampling methods (e.g., convenience sampling, random sampling)	
Geographic region	
Geographical location (rural, urban mixed)	
Recruitment setting (Community or clinical settings	
Study design (e.g., cohort study, cross-sectional study)	
Time period of data collection	
Type of mental health condition(s) assessed (i.e. anxiety, depression, and psychological distress)	
Structured diagnostic clinical interview	Yes/No
Name of diagnostic clinical interview	
Screening tool	Yes/No
Type of screening tool (self- report, clinician or proxy administered),	
Name of screening tool	
Prevalence reported	
Prevalence type (point, period, lifetime prevalence)	
Result Summary	
Results Summary Prevalence[a]	
Binary prevalence data format (n/N) of anxiety, depression and/ or psychological distress	
Percentage of the proportion of sample reported with either current or period or life time prevalence with 95% Confidence Intervals	

Social determinants summary				
Domains of the Social determinants	Proximal factors identified		Distal factors identified	
	Risk factors	Protective factors	Risk factors	Protective factors
Demographic domain				
Economic domain				
Environmental domain				
Neighbourhood domain				
Social and cultural domain				
Reviewer comments, if any?				

[a]Prevalence data will be extracted for all conditions, namely, depression, anxiety and/or psychological distress

For peer review only

BMJ Open

Prevalence and social determinants of anxiety and depression among adults in Ghana: a systematic review and meta-analysis protocol

Journal:	*BMJ Open*
Manuscript ID	bmjopen-2023-081927.R1
Article Type:	Protocol
Date Submitted by the Author:	06-Mar-2024
Complete List of Authors:	Awortwe, Victoria; Uppsala University, Healthcare Sciences and e-Health, Department of Women's and Children's Health<br>Daivadanam, Meena ; Uppsala University, Global Health and Migration Unit, Department of Women's and Children's Health<br>Adjorlolo, Samuel ; University of Ghana, Department of Mental Health, School of Nursing & Midwivery, College of Health Sciences<br>Olsson, Erik; Uppsala University, Healthcare Sciences and e-Health, Department of Women's and Children's Health<br>Coumoundouros, Chelsea; Uppsala University, Healthcare Sciences and e-Health, Department of Women's and Children's Health<br>Woodford, Joanne; Uppsala University, Healthcare Sciences and e-Health, Department of Women's and Children's Health
**Primary Subject Heading**:	Mental health
Secondary Subject Heading:	Epidemiology, Public health, Global health
Keywords:	Adult psychiatry < PSYCHIATRY, EPIDEMIOLOGY, MENTAL HEALTH, PUBLIC HEALTH, Systematic Review

SCHOLARONE™
Manuscripts

Prevalence and social determinants of anxiety and depression among adults in Ghana: a systematic review and meta-analysis protocol

Victoria AWORTWE[1] (victoria.awortwe@uu.se), Meena DAIVADANAM[2] (meena.daivadanam@kbh.uu.se), Samuel ADJORLOLO[3] (sadjorlolo@ug.edu.gh), Erik OLSSON[1] (erik.olsson@kbh.uu.se), Chelsea COUMOUNDOUROS[1]* (chelsea.coumoundouros@kbh.uu.se), Joanne WOODFORD[1]* (joanne.woodford@kbh.uu.se)

*Joint last authorship

[1]Healthcare Sciences and e-Health, Department of Women's and Children's Health, Uppsala University, Uppsala, Sweden.

[2]Global Health and Migration Unit, Department of Women's and Children's Health, Uppsala University, Uppsala, Sweden.

[3]Department of Mental Health, School of Nursing and Midwifery, College of Health Sciences, University of Ghana, Accra, Ghana.

Corresponding author

Victoria Awortwe

For peer review only

ABSTRACT

Introduction: Anxiety and depression pose a significant global health challenge, especially affecting adults in low-and middle-income countries. In many low and middle-income countries, including those in sub-Saharan Africa, social determinants such as access to affordable health services, conflict, food insecurity, and poverty may be associated with the prevalence of anxiety and depression, further contributing to health disparities. To mitigate the burden of anxiety and depression in sub-Saharan Africa, it is essential to develop country-level tailored mental health policies and strategies. For example, Ghana is working towards improving mental health via its 12-year Mental Health policy launched in 2021. However, the prevalence of anxiety and depression among adults in Ghana, along with associated social determinants remains largely unknown, posing challenges for mental health planning, resource allocation, and developing targeted interventions. This review aims to examine the prevalence of anxiety and depression among adults in Ghana, and explore social determinants potentially associated with anxiety and depression.

Methods and analysis: Electronic databases (e.g., African Index Medicus, CINAHL, EMBASE, MEDLINE, PsycINFO) will be searched with all screening steps conducted by two independent reviewers. Secondary search strategies, including grey literature searches, will be used. Studies reporting on the prevalence of anxiety, depression, and/or a combined symptom measure (i.e. psychological distress) among adults in Ghana, using validated instruments will be included. If data allows, random-effects-meta-analyses will be performed to estimate pooled prevalence rates of anxiety and depression. Potential clinical and methodological moderators will be examined using subgroup analyses and meta-regression. A narrative synthesis will explore social determinants potentially associated with anxiety and depression among adults in Ghana.

Ethics and dissemination: Ethical approval is not required as no primary data will be collected. Results will be disseminated via a peer-reviewed publication and presentations at academic conferences. Plain language summaries will be provided to relevant non-governmental organizations working in Ghana.

PROSPERO registration number: CRD42023463078

For peer review only

For peer review only - http://bmjopen.bmj.com/site/about/guidelines.xhtml

Strengths and limitations of this study

- This review, to our knowledge, is the first of its kind, aiming to examine the prevalence of anxiety and depression among adults in Ghana and explore the social determinants potentially associated with anxiety and depression.
- The review protocol adheres to quality standards informed by the Preferred Reporting Items for Systematic Reviews and Meta-Analysis Protocols (PRISMA-P) checklist, with all screening steps and quality appraisal conducted by two independent reviewers.
- The peer-reviewed, comprehensive search strategy will ensure the inclusion of a wide range of relevant studies, reducing the risk of selection bias.
- High levels of heterogeneity across studies due to differences in populations, condition measurements and study designs may limit the possibility of conducting a meta-analysis.
- Qualitative studies will be excluded therefore limiting an in-depth exploration of the perspectives of adults in Ghana on social determinants associated with their mental health, which may have provided important information to inform future intervention development.

For peer review only

INTRODUCTON

Common mental disorders, such as anxiety and depression are among the most prevalent and rising health problems affecting adults worldwide.[1, 2] Anxiety and depression are leading causes of health-related burden globally, with depression being the second and anxiety being the eighth leading cause of years lived with disability (YLDs) out of 369 diseases and injuries in 204 countries and territories.[1] The consequences of anxiety and depression can be severe; resulting in impaired social functioning, increased mortality rates, low productivity, and reduced quality of life.[3] Research suggests anxiety and depression are associated with an increased risk of developing complications or worsening a range of chronic physical conditions including cancer, diabetes, heart disease, and human immunodeficiency virus (HIV).[4–6] The economic burden of anxiety and depression is also substantial with costs stemming from healthcare service use, lost productivity, and impact on families, caregivers, and wider society.[7, 8] The burden and impact of anxiety and depression on individuals and societies underscores the critical need to prioritize adult mental health and well-being globally.

The majority of the global burden of mental disorders is located in low-and middle-income countries (LMICs),[9] where 75% of individuals experiencing common mental disorders do not receive appropriate mental healthcare.[10] This treatment gap is particularly pronounced in Africa owing to the limited availability of mental health resources, coupled with healthcare systems that are inadequately equipped to meet the needs of individuals seeking care.[11, 12] A previous scoping review conducted in 12 African countries found a lifetime prevalence rate of anxiety ranging from 5.7% to 15.8% and depression ranging from 3.3% to 9.8%.[13] Within Africa, the prevalence of mental disorders including anxiety and depression is expected to increase by 130% between 2010 and 2050, potentially leading to 45 million YLDs in sub-Saharan Africa (SSA),[14] with major depression predicted to be the largest contributor to disease burden in SSA.[14] Therefore, researchers, global health entities, and advocacy groups are urging actions to address the growing mental health crisis in SSA,[15] advocating for integrating mental health into primary care, establishing national policies, training paraprofessionals, expanding community healthcare, and collaborating with traditional healers.[14, 16] To further inform these actions,[15] there is a need to better understand the determinants of mental health that may contribute to the development and exacerbation of mental disorders in SSA, particularly in resource-constrained countries in the region.

For peer review only

Determinants of mental health among adults are multifaceted, and can be broadly categorized into biological (e.g., genetics, brain chemistry, and hormonal imbalances), psychological (e.g., cognitive and interpersonal factors), and social (e.g., ethnicity, food security, neighborhood deprivation).[17] Social determinants can also exacerbate biological and psychological vulnerabilities via mechanisms such as gene-environment interactions, epigenetic modifications, and maladaptive coping.[18–20] Globally, social determinants are recognized for their role in either providing protection or increasing the risk of adult mental disorders such as anxiety and depression, while also contributing to health disparities.[21–23] Within LMICs, including those in Africa, social determinants such as including diminished social capital, environmental events, food insecurity, forced migration, income inequality, violence, low education, poor housing, poverty, and unemployment,[24–27] may increase vulnerability to experiencing mental health difficulties.[15] Recognizing the importance of social determinants, the World Health Organization (WHO) have urged focus on reshaping economic, physical, and social factors to improve mental health and reduce inequalities by accelerating the implementation of the Comprehensive Mental Health Action Plan 2013-2030.[28] Importantly, the majority of social determinants such as poor living arrangements and low or worsening socioeconomic status are modifiable,[29] and may be improved by implementing appropriate governmental policies and adopting a multi-sectoral approach to provide comprehensive mental health and social care services.[7, 30] In SSA, there is a growing consensus that mental health policies and services should be tailored to the contextual realities of each country in the region, with evidence emphasizing the importance of documenting the social determinants specific to each SSA country where mental disorders are experienced.[15, 31]

The focus of the present review is Ghana, a LMIC in SSA with a population of approximately 31 million, with an estimated 13% of adults experiencing mental disorders including anxiety and depression.[32] Despite this, only 2% receive treatment.33 Chronic physical conditions like cancer, diabetes, and HIV are commonly linked to mental disorders among adults in Ghana[34, 35] and the burden of anxiety and depression in Ghana is further worsened by inadequate mental health resources and healthcare disparities.[35] Social determinants such as crime, food insecurity, poor sanitation, poverty and unemployment may also contribute to elevated rates of mental disorders in adults in Ghana.[35–38] To our knowledge, there has been no systematic review undertaken to consolidate existing research, determine the prevalence rates of anxiety and depression among adults; and explore key social determinants specific to

For peer review only - http://bmjopen.bmj.com/site/about/guidelines.xhtml

the Ghanaian context. By determining the prevalence of anxiety and depression among adults in Ghana and exploring social determinants potentially associated with anxiety and depression, this review will provide insights into the magnitude of the problem in Ghana, and social determinants influencing their occurrence to inform evidence-based policies and interventions aimed at enhancing mental well-being and overall health of adults in Ghana. The review's findings will also be valuable for countries experiencing similar social determinants as the Ghanaian population.

Research Objectives

This systematic review seeks to: (1) examine the prevalence of anxiety, depression, and psychological distress among adults in Ghana and (2) explore the social determinants potentially associated with anxiety, depression and psychological distress.

METHODS

This review protocol adheres to the Preferred Reporting Items for Systematic Reviews and Meta-Analysis Protocols (PRISMA-P) checklist[39] (online supplementary appendix 1). The Joanna Briggs Institute (JBI) methodology for systematic reviews on prevalence was used to guide protocol development.[40] The protocol has been registered in the International Prospective Register of Systematic Reviews (PROSPERO, registration number CRD42023463078). The study is scheduled to commence on September 25, 2023, with an anticipated completion date of September 1, 2024. Any protocol amendments will be recorded in PROSPERO.

Eligibility criteria

Eligibility criteria was developed and defined in accordance with the CoCoPop (Condition, Context, and Population) and type of studies framework.[41]

Population

Adults (aged ≥ 18 years) living in Ghana will be eligible for inclusion. Studies solely focused on children/adolescents will be excluded. Studies focusing on both adults and adolescents will be excluded if they do not present data separately for adults and adolescents or data cannot be obtained via correspondence with study authors. Studies conducted with specific sub-

For peer review only - http://bmjopen.bmj.com/site/about/guidelines.xhtml

populations including individuals with known psychiatric conditions, prisoners, individuals accused of witchcraft, and women with fertility or gynaecological disorders, will be excluded due to their unique circumstances, which predispose them to a higher risk or potentially elevate the likelihood of experiencing mental health problems compared to the general population.

Condition

Conditions eligible for inclusion include anxiety and / or depression assessed using a structured diagnostic clinical interview in accordance with the International Classification of Diseases and Related Health Problems (ICD-10 or ICD-11), or the Diagnostic and Statistical Manual of Mental disorders (DSM), third, fourth or fifth edition (DSM-III, DSM-IV or DSM-V) such as the Structured Clinical Interview for (SCID), the Mini-International Neuropsychiatric Interview (MINI), or the Composite International Diagnostic Interview (CIDI). Studies using a self-report, clinician or proxy administered screening tool for anxiety (e.g., Beck Anxiety Inventory), depression (e.g., Beck Depression Inventory), or psychological distress (e.g., Kessler Psychological Distress Scale) will also be eligible for inclusion. Studies reporting point (current), period (timepoint), or lifetime estimates of the prevalence of anxiety, depression and psychological distress will be included.[42] To ensure the reliability and quality of assessment methods, only studies using instruments validated for use in adult populations will be included. Validity will be assessed based on evidence provided in the validation paper(s) of the measurement instrument and/or evidence of psychometric properties such as construct validity, content validity, criterion validity, and reliability measures including internal consistency, test-retest reliability, and inter-rater reliability. Studies using instruments validated for use in adult samples in Ghana will also be eligible for inclusion. Studies solely focused on other mental disorders (e.g., psychotic disorders, bipolar affective disorders), substance use and neurological disorders (e.g., alcohol dependence, multiple sclerosis) will be excluded. Studies will be excluded if the prevalence of anxiety, depression and psychological distress cannot be calculated, e.g., when reported solely as mean score or due to insufficient data.

Context

This review will include studies conducted in Ghana with adults sampled from the community or clinical settings (e.g., primary healthcare facilities, hospitals). Studies conducted in regions that encompass Ghana (e.g., West Africa and Sub-Saharan Africa) will be considered eligible if data on participants living in Ghana can be extracted from the publication or obtained via correspondence with study authors. Studies conducted immediately after conflict (i.e., less than

For peer review only - http://bmjopen.bmj.com/site/about/guidelines.xhtml

four months after the official end date of the conflict),[43] humanitarian crises, or natural disaster will be excluded given we seek to understand the general prevalence of anxiety and depression.[44]

Types of studies

Primary quantitative studies, with observational study designs including longitudinal cohort studies (baseline data only), case-control, and cross-sectional studies reporting the prevalence of anxiety, depression and/or psychological distress among adults in Ghana will be included. Mixed method studies will be eligible for inclusion only if data from the quantitative component can be clearly extracted. In the case of studies conducted on the same cohort of individuals at the same or different points in time; or where samples overlap, only the study with the largest sample and findings related to the aims of this review will be included to ensure duplicate data is not included. Studies such as case reports, commentaries, conference proceedings, editorials, letters, opinion papers, qualitative studies, reviews, theses/dissertations will be excluded.

Information sources

Searches will be conducted in accordance with PRISMA 2020 guidelines.[45] Electronic database searches will be carried out in African Index Medicus (AIM), African Journals Online (AJOL), Cumulative Index to Nursing and Allied Health Literature (CINAHL), Excerpta Medica Database (Embase), Ghana Medical Journal (GMJ), Health Sciences Investigation (HIS), MEDLINE (PubMed), PsycINFO and SCOPUS. Electronic databases will be searched from inception up to September 25, 2023, and updated study searches will be conducted within 3 months to submitting the results manuscript. Reference lists of included studies will be manually checked and forward citation checks of included studies will be performed. Reference lists of relevant systematic reviews conducted in sub-Saharan Africa, including Ghana will also be manually checked. Grey literature will be searched in Agency for Healthcare Research and Quality (AHRQ), Google Scholar, Health Systems Trust, Open Grey (http://www. opengrey.eu/) and the World Health Organization (WHO). Researchers and non-governmental organizations working in the area of adult mental health in Ghana will be contacted to identify unpublished or ongoing studies.

For peer review only - http://bmjopen.bmj.com/site/about/guidelines.xhtml

Search strategy

The search strategy has been developed in collaboration with Mattias Axén, a librarian at Uppsala University Library and was reviewed by Alkistis Skalkidou and Lene Lindberg, following the PRESS Peer Review guidelines[46] (online supplementary appendix 2). The search was constructed using terms related to (1) mental disorders and (2) Ghana (see online supplementary appendix 3). Electronic databases will be searched using Medical Subject Headings (MeSH) when possible and free text words in title and abstract word searches. No date restriction will be imposed and only studies published in English and Ghanaian languages (e.g., Fante, Ga, Twi) will be considered for inclusion.

Study selection

Studies retrieved from searches will be uploaded into Endnote 20 with duplicates identified and removed. Two reviewers will independently screen titles and abstracts in Rayyan,[47] followed by full paper checks of potentially eligible studies. Studies not meeting the eligibility criteria will be excluded. Overall reasons for exclusion will be documented and reported using the PRISMA flow chart and detailed reasons for exclusion, in accordance with the eligibility criteria, will be presented in a table. If study data/information required to determine eligibility is missing, authors will be contacted via email for additional information. Any disagreement between reviewers will be resolved by discussion and/ or involvement of a third reviewer.

Data extraction

Data from included studies (see online supplementary appendix 4), will be extracted by one reviewer independently into a standardized Microsoft Excel data extraction form, and crosschecked by a second reviewer for accuracy. The following data will be extracted:

1. Study identification features: study ID/record number, study title and aim, first author name, year of publication, and publication type or data source (e.g., journal, or report).
2. Study characteristics: characteristics of study population (age, gender, socioeconomic status, chronic physical condition reported), sample size, sampling methods (e.g., convenience sampling, random sampling), study setting including geographical region(s) in Ghana where study was conducted, location (rural, urban, mixed), and

For peer review only - http://bmjopen.bmj.com/site/about/guidelines.xhtml

recruitment setting (community or clinical settings), study design (e.g., case-control, cross-sectional, longitudinal cohort studies), time period of data collection, type of mental health condition (anxiety, depression and psychological distress), structured diagnostic clinical interview (yes/no), name of diagnostic clinical interview, screening tool (yes/no), type of screening tool (self-report, clinician or proxy administered), name of screening tool and cutoff scores, evidence of validity of the measurement instrument, as well as evidence of ethical approval.

3. Results summary: binary prevalence data of anxiety, depression and psychological distress (i.e. number of cases/ the total sample size, n/N) and percentage with 95% confidence intervals, prevalence type (current, period or life time) and summary of social determinants classified into demographic, economic, environmental, neighbourhood, social and cultural domains.

Quality assessment

The methodological quality of all included studies will be independently assessed by two reviewers using the standardized JBI Critical Appraisal Tool for Prevalence Studies.[48] The JBI Critical Appraisal Tool for Prevalence Studies comprises nine items including: appropriateness of sampling frame and sampling technique, adequacy of sample size, coverage of identified sample, description of study setting and subjects, validity of condition identification methods, standard and reliable measurement of condition, statistical analysis, and adequacy of response rate to assess the methodological quality and/or risk of bias of sampling, analysis and measurements in primary studies. The total score ranges from 1 to 9 for individual studies, [49] with the total number of "yes" scores for individual studies averaged to appraise studies as low ($\leq$ 3 score), moderate (4-6 points) or high quality ($\geq$ 7 points).[50] Any disagreement between reviewers will be resolved by discussion and / or involvement of a third reviewer.

Data analysis and synthesis

Quantitative data synthesis

If data allows, a meta-analysis with prevalence data from eligible studies will be conducted using Comprehensive Meta-Analysis software.[51] Data on the proportion of adults with

For peer review only - http://bmjopen.bmj.com/site/about/guidelines.xhtml

anxiety, depression and psychological distress and their respective sample size will be extracted separately from individual studies to generate pooled estimates with exact binomial test and associated 95% confidence intervals. Data will be transformed to their logits before meta-analysis to stabilize variances.[52] Due to expected heterogeneity in individual studies, the random-effect model will be used to generate pooled prevalence estimates for anxiety, depression, and psychological distress respectively.[51] Heterogeneity across studies will be estimated using Cochran's Q statistic. The I^2 statistics will be used to measure the proportion of total variability due to between-study heterogeneity. The prediction interval (T^2) will be used as an estimate of between-study variance in true effects observed in eligible studies. Sensitivity analyses will be performed to explore the impact of individual studies on the overall prevalence estimate of anxiety and depression. This will be conducted for example, by removing studies conducted during COVID or studies of lower quality individually from the overall analysis to ascertain if their removal causes any substantial change to overall prevalence estimates. Egger's regression statistic and funnel plots will be used to assess the presence of any publication bias. In the case of significant publication bias,[53] the trim & fill method will be used to identify and correct the asymmetry of the funnel plot to yield a corrected pooled prevalence.

Subgroup analysis

If data allows, we will explore sources of heterogeneity via subgroup analysis of the moderating effects of the following factors on prevalence:

- Chronic physical conditions (e.g., long-term life-threatening conditions such as cancer, diabetes, emphysema, hypertension, HIV/AIDS, ischemic heart disease, and stroke, and chronic conditions such as arthritis, asthma, back problems of any kind, chronic bronchitis, gall bladder diseases, joint pain, osteoporosis and stomach ulcers).[54]
- Method of mental health assessment (e.g., structured clinical interview or screening tool).
- Sample size (<100 vs. ≥100).
- Study design (e.g., case-control study, cross-sectional studies, longitudinal cohort study).
- Study quality (low, medium or high-quality studies).
- Prevalence type (point, period, lifetime).

For peer review only - http://bmjopen.bmj.com/site/about/guidelines.xhtml

- Time period of data collection (e.g., <2013 vs. ≥2013 or <2020 vs. ≥2020).

Narrative synthesis

Social determinants will be narratively synthesized, with specific distal and/or proximal factors grouped under domains of the social determinants of mental disorders and Sustainable Development Goals (SDGs) framework,[22] outlined below:

1. Demographics: proximal factors (e.g., age, ethnicity, gender) and distal factors (e.g., community diversity, population density).

2. Economic: proximal factors (e.g., assets, debt, unemployment) and distal factors (e.g., economic inequality, recessions).

3. Neighbourhood: proximal factors (e.g., safety and security, housing structure, overcrowding) and distal factors (e.g., neighbourhood deprivation, built environment).

4. Environmental events: proximal factors (e.g., trauma) and distal factors (climate change, forced migration, war or conflict).

5. Social and cultural: proximal factors (e.g., individual social capital, social participation, education) and distal factors (e.g., social stability, community social capital).

Patient and public involvement

Public contributors were not involved in the development of this protocol and will not be involved in the conduct of the review due to time and resource limitations. We will seek to involve public contributors in co-writing plain language summaries to be provided to non-governmental organizations working in the area of adult mental health in Ghana.

DISCUSSION

To the best of our knowledge, there is currently no comprehensive review examining the prevalence and social determinants of anxiety and depression among adults in Ghana. This review will extend existing epidemiology literature on mental health[36, 55, 56] by providing a comprehensive summary of prevalence estimates of anxiety and depression among adults in Ghana, as well as social determinants potentially influencing their occurrence. The review has

For peer review only - http://bmjopen.bmj.com/site/about/guidelines.xhtml

a number of strengths including a peer-reviewed, comprehensive search strategy ensuring the inclusion of a wide range of relevant studies, and reducing the risk of selection bias. By considering comorbidities, the review recognizes that mental health difficulties often co-occur with physical conditions, contributing to a better understanding of the overall health and mental well-being of adults in Ghana. Comorbidity between anxiety and depression is frequent and if data permits, the prevalence of comorbid anxiety and depression will be documented. The review protocol also adheres to quality standards informed by the PRISMA-P checklist,[39] with screening, selection, and quality appraisal assessed by two independent reviewers.

While there are several strengths, there are also limitations. Qualitative studies will be excluded, limiting an in-depth exploration of the perspectives of adults in Ghana on social determinants associated with their mental health, which may have provided important information to inform mental health intervention development. High levels of clinical and methodological heterogeneity across studies due to factors such as differences in populations, condition measurements and study design may limit the possibility of conducting a meta-analysis. This situation may warrant the need for narrative synthesis.

Despite these limitations, the significance of this review is highlighted by the widespread global attention on mental health, with initiatives such as the WHO Comprehensive Mental Health Action Plan 2013-2030 and the United Nation's Sustainable Developmental Goals (SDGs) highlighting the importance of mental healthcare equity, prevention, treatment, and promotion.[57] Whilst Ghana is working towards improving mental health via its revised 12-year Mental Health Policy launched in 2021,[35] a comprehensive review examining the prevalence of anxiety and depression among adults in Ghana can facilitate the planning and allocation of resources for mental healthcare.[35] Addressing the social determinants is also crucial for achieving broader development targets, such as SDG 1 for no poverty, SDG 5 for gender equality, and SDG 4 for quality education, among others.[22] Findings may be used to inform future research, mental health-care planning, and the development of culturally responsive interventions aimed at improving the mental well-being and overall health of adults in Ghana.

For peer review only - http://bmjopen.bmj.com/site/about/guidelines.xhtml

ETHICS AND DISSEMINATION

Findings will be disseminated through scientific publication in a peer-reviewed journal and as presentations at conferences. The authors will use scientific presentations at institutional events, regional and international conferences and social media to promote the review result. Plain language summaries will be provided to non-governmental organizations working in the area of adult mental health in Ghana.

Acknowledgments Alkistis Skalkidou (AS) from the Department of Women's and Children's Health at Uppsala University and Lene Lindberg (LL) from the Department of Public Health Sciences at Karolinska Institute for providing peer review of the search strategy. Mattias Axén (MA), a Librarian at Uppsala University Library for assisting with the development of the electronic search strategy. Febrina Maharani (FM), master student in Global Health at Uppsala University for assisting with study selection process.

Contributors: VA, MD and JW conceptualized the study. VA drafted the proposal. VA, CC and JW designed the study. All authors (VA, MD, EO, SA, CC and JW) assisted with manuscript writing and critical revision of the study design and manuscript. All authors read and approved the final manuscript. JW is the guarantor of the review.

Funding This work was supported by U-CARE, which is a strategic research environment funded by the Swedish Research Council (dnr 2009–1093).

Disclaimer This funding source had no role in the design of this study and will not have any role during its execution, analyses, interpretation or the data or decision to submit results.

Competing interests None declared.

Patient consent for publication Not required.

Ethics approval This study protocol is for a systematic review, meta- analysis and meta synthesis using secondary data. No raw individual level data is included within this review, with only group level data extracted and analysed, as such no formal ethical approval is required.

Provenance and peer review Not commissioned; externally peer reviewed.

Data availability statement Not applicable.

For peer review only

References

1. GBD 2019 Mental Disorders Collaborators. Global, regional, and national burden of 12 mental disorders in 204 countries and territories, 1990-2019: a systematic analysis for the Global Burden of Disease Study 2019. Lancet Psychiatry. 2022;9(2):137–50.

2. Hyman S, Parikh R, Collins PY, Patel V. Adult Mental Disorders. In: Mental, Neurological, and Substance Use Disorders: Disease Control Priorities, Third Edition (Volume 4) [Internet]. The International Bank for Reconstruction and Development / The World Bank; 2016 [cited 2023 Apr 28]. Available from: https://www.ncbi.nlm.nih.gov/books/NBK361952/

3. Ormel J, VonKorff M. Reducing common mental disorder prevalence in populations. JAMA Psychiatry. 2021;78(4):359–60.

4. Daré LO, Bruand PE, Gérard D, Marin B, Lameyre V, Boumédiène F, et al. Co-morbidities of mental disorders and chronic physical diseases in developing and emerging countries: a meta-analysis. BMC Public Health. 2019;19(1):304.

5. Scott KM, Lim C, Al-Hamzawi A, Alonso J, Bruffaerts R, Caldas-de-Almeida JM, et al. Association of mental disorders with subsequent chronic physical conditions: World Mental Health Surveys from 17 countries. JAMA Psychiatry. 2016;73(2):150–8.

6. World Health Organisation. Comprehensive Mental Health Action Plan 2013-2030 [Internet]. 2021 [cited 2022 Dec 28]. Available from: https://www.who.int/publications-detail-redirect/9789240031029

7. World Health Organization. Comprehensive Mental Health Action Plan 2013-2030 [Internet]. Geneva: World Health Organization; 2021 [cited 2023 May 29]. Available from: https://www.who.int/publications-detail-redirect/9789240031029

8. World Health Organization and Calouste Gulbenkian Foundation. Social determinants of mental health [Internet]. World Health Organization, Geneva; 2014 [cited 2023 Apr 28]. Available from: https://www.who.int/publications-detail-redirect/9789241506809

9. World Health Organization. Depression and Other Common Mental Disorders: Global Health Estimates [Internet]. Geneva; 2017 [cited 2023 Mar 15]. Available from: https://www.who.int/publications-detail-redirect/depression-global-health-estimates

10. Dua T, Barbui C, Clark N, Fleischmann A, Poznyak V, Ommeren M van, et al. Evidence-based guidelines for mental, neurological, and substance use disorders in low- and middle-income countries: summary of WHO recommendations. PLOS Medicine. 2011;8(11): e1001122.

11. Lund C, Alem A, Schneider M, Hanlon C, Ahrens J, Bandawe C, et al. Generating evidence to narrow the treatment gap for mental disorders in sub-Saharan Africa: rationale, overview and methods of AFFIRM. Epidemiol Psychiatr Sci. 2015;24(3):233–40.

12. Sankoh O, Sevalie S, Weston M. Mental health in Africa. Lancet Glob Health. 2018;6(9): e954–5.

For peer review only - http://bmjopen.bmj.com/site/about/guidelines.xhtml

13. Greene MC, Yangchen T, Lehner T, Sullivan PF, Pato CN, McIntosh A, et al. The epidemiology of psychiatric disorders in Africa: a scoping review. Lancet Psychiatry. 2021;8(8):717–31.

14. Charlson FJ, Diminic S, Lund C, Degenhardt L, Whiteford HA. Mental and substance use disorders in Sub-Saharan Africa: predictions of epidemiological changes and mental health workforce requirements for the next 40 years. PLOS ONE. 2014;9(10): e110208.

15. Monteiro N. Addressing mental illness in Africa: global health challenges and local opportunities. Community Psychol Glob Perspect. 2015; 1:78–95.

16. Atilola O. Cross-cultural child and adolescent psychiatry research in developing countries. Glob Ment Health. 2015;2: e5.

17. Patel V, Saxena S, Lund C, Thornicroft G, Baingana F, Bolton P, et al. The Lancet Commission on global mental health and sustainable development. Lancet. 2018;392(10157):1553–98.

18. Compton MT, Shim RS. The social determinants of mental health. FOC. 2015 Oct;13(4):419–25.

19. Gottschalk MG, Domschke K. Novel developments in genetic and epigenetic mechanisms of anxiety. Curr Opin Psychiatry. 2016;29(1):32–8.

20. Remes O, Mendes JF, Templeton P. Biological, psychological, and social determinants of depression: a review of recent literature. Brain Sci. 2021;11(12):1633.

21. Alegría M, NeMoyer A, Falgàs Bagué I, Wang Y, Alvarez K. Social determinants of mental health: where we are and where we need to go. Curr Psychiatry Rep. 2018;20(11):95.

22. Lund C, Brooke-Sumner C, Baingana F, Baron EC, Breuer E, Chandra P, et al. Social determinants of mental disorders and the Sustainable Development Goals: a systematic review of reviews. Lancet Psychiatry. 2018;5(4):357–69.

23. Moitra M, Owens S, Hailemariam M, Wilson KS, Mensa-Kwao A, Gonese G, et al. Global mental health: where we are and where we are going. Curr Psychiatry Rep. 2023;25(7):301–11.

24. Deglon M, Dalvie MA, Abrams A. The impact of extreme weather events on mental health in Africa: a scoping review of the evidence. Sci Total Environ. 2023; 881:163420.

25. Hawkins C, Bwanika JM, Ibanda M. Socio-economic factors associated with mental health disorders in Fort Portal, western Uganda. S Afr J Psychiatr. 2020; 26:1391.

26. Musisi S, Kinyanda E. Long-Term Impact of War, Civil War, and Persecution in Civilian Populations—Conflict and Post-Traumatic Stress in African Communities. Frontiers in Psychiatry [Internet]. 2020 [cited 2023 Oct 23];11. Available from: https://www.frontiersin.org/articles/10.3389/fpsyt.2020.00020

27. Robertson LJ. The impact of urbanization on mental health service provision: a Brazil, Russia, India, China, South Africa and Africa focus. Curr Opin Psychiatry. 2019;32(3):224.

For peer review only - http://bmjopen.bmj.com/site/about/guidelines.xhtml

28. World Health Organization. World mental health report: Transforming mental health for all [Internet]. Geneva; 2022 [cited 2023 May 8]. Available from: https://www.who.int/publications-detail-redirect/9789240049338

29. Furber G, Leach M, Guy S, Segal L. Developing a broad categorisation scheme to describe risk factors for mental illness, for use in prevention policy and planning. Aust N Z J Psychiatry. 2017;51(3):230–40.

30. Yu S. Uncovering the hidden impacts of inequality on mental health: a global study. Transl Psychiatry. 2018;8(1):98.

31. Adhvaryu A, Fenske J, Nyshadham A. Early life circumstance and adult mental health. J Polit Econ. 2019;127(4):1516–49.

32. Oppong S, Kretchy IA, Imbeah EP, Afrane BA. Managing mental illness in Ghana: the state of commonly prescribed psychotropic medicines. Int J Ment Health Syst. 2016;10(1):28.

33. Eaton J, Ohene S. Providing Sustainable Mental Health Care in Ghana: A Demonstration Project. In: Providing Sustainable Mental and Neurological Health Care in Ghana and Kenya: Workshop Summary [Internet]. National Academies Press (US); 2016 [cited 2023 Jun 9]. Available from: https://www.ncbi.nlm.nih.gov/books/NBK350318/

34. de-Graft Aikins A, Koram K. Health and Healthcare in Ghana, 1957-2017. In: The Economy of Ghana: Sixty Years after Independence. Oxford University Press; 2017.

35. Government of Ghana. Mental Health Policy 2019 -2030: Ensuring A Mentally Healthy Population [Internet]. 2018 [cited 2023 May 8]. Available from: https://extranet.who.int/mindbank/item/7134

36. Amu H, Osei E, Kofie P, Owusu R, Bosoka SA, Konlan KD, et al. Prevalence and predictors of depression, anxiety, and stress among adults in Ghana: a community-based cross-sectional study. PLoS One. 2021;16(10): e0258105.

37. Atuoye KN, Luginaah I. Food as a social determinant of mental health among household heads in the Upper West Region of Ghana. Soc Sci Med. 2017; 180:170–80.

38. Greif MJ, Nii-Amoo Dodoo F. How community physical, structural, and social stressors relate to mental health in the urban slums of Accra, Ghana. Health Place. 2015; 33:57–66.

39. Moher D, Shamseer L, Clarke M, Ghersi D, Liberati A, Petticrew M, et al. Preferred reporting items for systematic review and meta-analysis protocols (PRISMA-P) 2015 statement. Syst Rev. 2015;4(1):1.

40. Munn Z, Moola S, Lisy K, Riitano D, Tufanaru C. Methodological guidance for systematic reviews of observational epidemiological studies reporting prevalence and cumulative incidence data. JBI Evid Implement. 2015;13(3):147.

41. Moola S, Munn Z, Sears K, Sfetcu R, Currie M, Lisy K, et al. Conducting systematic reviews of association (etiology): the Joanna Briggs Institute's approach. JBI Evid Implement. 2015;13(3):163.

For peer review only

For peer review only - http://bmjopen.bmj.com/site/about/guidelines.xhtml

42. Steel Z, Marnane C, Iranpour C, Chey T, Jackson JW, Patel V, et al. The global prevalence of common mental disorders: a systematic review and meta-analysis 1980–2013. Int J Epidemiol. 2014;43(2):476–93.

43. Lim ICZY, Tam WWS, Chudzicka-Czupała A, McIntyre RS, Teopiz KM, Ho RC, et al. Prevalence of depression, anxiety and post-traumatic stress in war- and conflict-afflicted areas: a meta-analysis. Front Psychiatry. 2022; 13:978703.

44. Edwards N, Walker S, Paddick SM, Prina AM, Chinnasamy M, Reddy N, et al. Prevalence of depression and anxiety in older people in low- and middle- income countries in Africa, Asia and South America: a systematic review and meta-analysis. J Affect Disord. 2023; 325:656–74.

45. Page MJ, McKenzie JE, Bossuyt PM, Boutron I, Hoffmann TC, Mulrow CD, et al. The PRISMA 2020 statement: an updated guideline for reporting systematic reviews. BMJ. 2021;372: n71.

46. McGowan J, Sampson M, Salzwedel DM, Cogo E, Foerster V, Lefebvre C. PRESS peer review of electronic search strategies: 2015 guideline statement. J Clin Epidemiol. 2016; 75:40–6.

47. Ouzzani M, Hammady H, Fedorowicz Z, Elmagarmid A. Rayyan—a web and mobile app for systematic reviews. Systematic Reviews. 2016;5(1):210.

48. Munn Z, Moola S, Riitano D, Lisy K. The development of a critical appraisal tool for use in systematic reviews addressing questions of prevalence. Int J Health Policy Manag. 2014;3(3):123–8.

49. Opio JN, Munn Z, Aromataris E. Prevalence of mental disorders in Uganda: a systematic review and meta-analysis. Psychiatr Q. 2022;93(1):199–226.

50. Aarah-Bapuah M, Sarpomaa Oppong S, Ohenewaa Yawson A, Dzansi G, Adjorlolo S. Covid-19 and mental health of children and adolescents: a systematic review. Cogent Psychology. 2022;9(1):2111849.

51. Borenstein M. Comprehensive Meta-Analysis Software. In 2022. p. 535–48.

52. Doi SAR, Williams GM. Methods of Clinical Epidemiology. Springer Science & Business Media; 2013. 289 p.

53. Sutton AJ, Song F, Gilbody SM, Abrams KR. Modelling publication bias in meta-analysis: a review. Stat Methods Med Res. 2000;9(5):421–45.

54. Bhattacharya R, Shen C, Sambamoorthi U. Excess risk of chronic physical conditions associated with depression and anxiety. BMC Psychiatry. 2014;14(1):10.

55. Adu MK, Wallace LJ, Lartey KF, Arthur J, Oteng KF, Dwomoh S, et al. Prevalence and correlates of likely major depressive disorder among the adult population in Ghana during the COVID-19 Pandemic. Int J Environ Res Public Health. 2021;18(13):7106.

56. Ae-Ngibise KA, Sakyi L, Adwan-Kamara L, Lund C, Weobong B. Prevalence of probable mental, neurological and substance use conditions and case detection at primary healthcare

For peer review only

For peer review only - http://bmjopen.bmj.com/site/about/guidelines.xhtml

facilities across three districts in Ghana: findings from a cross-sectional health facility survey. BMC Psychiatry. 2023;23(1):280.

57. Campion J, Javed A, Lund C, Sartorius N, Saxena S, Marmot M, et al. Public mental health: required actions to address implementation failure in the context of COVID-19. Lancet Psychiatry. 2022;9(2):169–82.

For peer review only

For peer review only - http://bmjopen.bmj.com/site/about/guidelines.xhtml

Prevalence and social determinants of anxiety and depression among adults in Ghana: a systematic review and meta-analysis protocol

PRISMA-P (Preferred Reporting Items for Systematic review and Meta-Analysis Protocols) 2015 checklist: recommended items to address in a systematic review protocol*

Section and topic	Item No	Checklist item	Location in text (page number)
**ADMINISTRATIVE INFORMATION**			
Title:			
Identification	1a	Identify the report as a protocol of a systematic review	1
Update	1b	If the protocol is for an update of a previous systematic review, identify as such	N/A
Registration	2	If registered, provide the name of the registry (such as PROSPERO) and registration number	2 & 6
Authors:			
Contact	3a	Provide name, institutional affiliation, e-mail address of all protocol authors; provide physical mailing address of corresponding author	1
Contributions	3b	Describe contributions of protocol authors and identify the guarantor of the review	14
Amendments	4	If the protocol represents an amendment of a previously completed or published protocol, identify as such and list changes; otherwise, state plan for documenting important protocol amendments	6
Support:			
Sources	5a	Indicate sources of financial or other support for the review	14
Sponsor	5b	Provide name for the review funder and/or sponsor	14
Role of sponsor or funder	5c	Describe roles of funder(s), sponsor(s), and/or institution(s), if any, in developing the protocol	14
**INTRODUCTION**			
Rationale	6	Describe the rationale for the review in the context of what is already known	5 & 6 &12 &13
Objectives	7	Provide an explicit statement of the question(s) the review will address with reference to participants, interventions, comparators, and outcomes (PICO)	6
**METHODS**			
Eligibility criteria	8	Specify the study characteristics (such as PICO, study design, setting, time frame) and report characteristics (such as years considered, language, publication status) to be used as criteria for eligibility for the review	6 & 7 & 8

Information sources	9	Describe all intended information sources (such as electronic databases, contact with study authors, trial registers or other grey literature sources) with planned dates of coverage	8
Search strategy	10	Present draft of search strategy to be used for at least one electronic database, including planned limits, such that it could be repeated	9 & supplementary appendix 3
Study records:			
Data management	11a	Describe the mechanism(s) that will be used to manage records and data throughout the review	9
Selection process	11b	State the process that will be used for selecting studies (such as two independent reviewers) through each phase of the review (that is, screening, eligibility and inclusion in meta-analysis)	9
Data collection process	11c	Describe planned method of extracting data from reports (such as piloting forms, done independently, in duplicate), any processes for obtaining and confirming data from investigators	9 & 10
Data items	12	List and define all variables for which data will be sought (such as PICO items, funding sources), any pre-planned data assumptions and simplifications	9 &10
Outcomes and prioritization	13	List and define all outcomes for which data will be sought, including prioritization of main and additional outcomes, with rationale	9 & 10
Risk of bias in individual studies	14	Describe anticipated methods for assessing risk of bias of individual studies, including whether this will be done at the outcome or study level, or both; state how this information will be used in data synthesis	10 & 11
Data synthesis	15a	Describe criteria under which study data will be quantitatively synthesised	11&12
	15b	If data are appropriate for quantitative synthesis, describe planned summary measures, methods of handling data and methods of combining data from studies, including any planned exploration of consistency (such as I^2, Kendall's τ)	11
	15c	Describe any proposed additional analyses (such as sensitivity or subgroup analyses, meta-regression)	11
	15d	If quantitative synthesis is not appropriate, describe the type of summary planned	12 &13
Meta-bias(es)	16	Specify any planned assessment of meta-bias(es) (such as publication bias across studies, selective reporting within studies)	11
Confidence in cumulative evidence	17	Describe how the strength of the body of evidence will be assessed (such as GRADE)	N/A

N/A not applicable

*** It is strongly recommended that this checklist be read in conjunction with the PRISMA-P Explanation and Elaboration (cite when available) for important clarification on the items. Amendments to a review protocol should be tracked and dated. The copyright for PRISMA-P (including checklist) is held by the PRISMA-P Group and is distributed under a Creative Commons Attribution Licence 4.0.**

From: Shamseer L, Moher D, Clarke M, Ghersi D, Liberati A, Petticrew M, Shekelle P, Stewart L, PRISMA-P Group. Preferred reporting items for systematic review and meta-analysis protocols (PRISMA-P) 2015: elaboration and explanation. BMJ. 2015 Jan 2;349(jan02 1):pg7647.

Supplementary Appendix 2

PRESS Guideline — Search Submission & Peer Review Assessment

SEARCH SUBMISSION: THIS SECTION TO BE FILLED IN BY THE SEARCHER

Searcher: Victoria Awortwe	Email: victoria.awortwe@uu.se
Date submitted: 31/8/2023	Date requested by:11/9/2023

Systematic Review Title:

Prevalence and social determinants of anxiety and depression among adults in Ghana: a systematic review and meta-analysis protocol

This search strategy is …

X	My PRIMARY (core) database strategy — First time submitting a strategy for search question and database
	My PRIMARY (core) strategy — Follow-up review NOT the first time submitting a strategy for search question and database. If this is a response to peer review, itemize the changes made to the review suggestions
	SECONDARY search strategy— First time submitting a strategy for search question and database
	SECONDARY search strategy — NOT the first time submitting a strategy for search question and database. If this is a response to peer review, itemize the changes made to the review suggestions

Database

(i.e., MEDLINE, CINAHL…): *[mandatory]*

MEDLINE

Interface

(i.e., Ovid, EBSCO…): *[mandatory]*

PubMed

Research Question

(Describe the purpose of the search) *[mandatory]*

For peer review only - http://bmjopen.bmj.com/site/about/guidelines.xhtml

This review aims (1) examine the prevalence of anxiety, depression, and psychological distress among adults in Ghana and (2) explore the social determinants potentially associated with anxiety, depression and psychological distress.

CoCoPop Format

(Outline the CoCoPop for your question — i.e., Condition, Context, Population, and Study Design — as applicable)

**Population**	Adults (individuals aged ≥ 18 years) living in Ghana
**Condition**	Anxiety (i.e. anxiety disorders, anxiety symptoms), depression (i.e. depression, depressive disorders), and psychological distress.
**Context**	Ghana, with adults sampled from the community or clinical settings (e.g., primary health care facilities, hospitals).
**Study design**	Observational study designs

Inclusion Criteria

(List criteria such as age groups, study designs, etc., to be included) *[optional]*

Population: Adults (aged ≥ 18 years) living in Ghana will be eligible for inclusion.

Condition: Anxiety and/or depression assessed using structured diagnostic clinical interview in accordance with the International Classification of Diseases and Related Health Problems (ICD-10 or ICD-11), or the Diagnostic and Statistical Manual of Mental disorders (DSM) third, fourth and fifth edition (DSM-III, DSM-IV or DSM-V); OR clinician or proxy administered screening tool for anxiety, depression and psychological distress. For the meta-analyses, studies reporting on the prevalence of anxiety, depression and psychological distress among adults in Ghana, measured using validated instruments will be included.

Context: Studies from Ghana, with adults sampled from the community or clinical settings (e.g., primary health care facilities, hospitals). Studies conducted in regions that encompass Ghana (e.g., West Africa and Sub-Saharan Africa) will be considered eligible if data on participants living in Ghana is available. No date restriction will be imposed and only studies published in English and Ghanaian languages (e.g., Fante, Ga, Twi) will be considered for inclusion.

Study design: Primary quantitative studies, with observational study designs including longitudinal cohort studies, case-control, and cross-sectional studies. Mixed method studies will be eligible for inclusion only if data from the quantitative component can be clearly extracted.

For peer review only

For peer review only - http://bmjopen.bmj.com/site/about/guidelines.xhtml

Exclusion Criteria

(List criteria such as study designs, date limits, etc., to be excluded) *[optional]*

Population: Studies solely focused on children/adolescents will be excluded. Studies focusing on both adults and adolescents will be excluded if they do not present data separately for adults and adolescents or data cannot be obtained via correspondence with study authors. Studies conducted with specific sub-populations including individuals with known psychiatric conditions, prisoners, individuals accused of witchcraft, and women with fertility or gynaecological disorders, will be excluded due to their unique circumstances, which predispose them to a higher risk or potentially elevate the likelihood of experiencing mental health problems compared to the general population.

Condition: Studies solely focused on other mental disorders (e.g., psychotic disorders, bipolar affective disorders), substance use and neurological disorders (e.g., alcohol dependence, multiple sclerosis) will be excluded. Studies will be excluded if the prevalence of anxiety, depression and psychological distress cannot be calculated, e.g., when reported solely as mean score or due to insufficient data.

Context: Studies that were conducted immediately after conflict, humanitarian crises, or natural disaster will be excluded.

Study design: Studies such as case reports, commentaries, conference proceedings, editorials, letters, opinion papers, qualitative studies, reviews, theses/dissertations; and studies published in languages other than English and Ghanaian will be excluded.

Was a search filter applied?

Yes ☐ No ■

If YES, which one(s) (e.g., Cochrane RCT filter, PubMed Clinical Queries filter)? Provide the source if this is a published filter. *[mandatory if YES to previous question — textbox]*

Other notes or comments you feel would be useful for the peer reviewer? ***[optional]***

We did not include search terms regarding (1) prevalence, as that is not the only area of interest; and (2) adults, as the term can be referred to in a variety of ways. Furthermore, we searched Ghana in All fields to accommodate studies conducted in regions that encompass Ghana (e.g., West Africa and Sub-Saharan Africa) providing data on participants living in Ghana.

Please copy and paste your search strategy here, exactly as run, including the number of hits per line. ***[mandatory]***

For peer review only - http://bmjopen.bmj.com/site/about/guidelines.xhtml

For peer review only

Database: PubMed. Final number of results from the full search 1297	
**1: Mental disorders**	**2: Ghana**
**Title & Abstract**<br>Mental health 228145<br>Mental disorders 51217<br>Mental disorder 11824<br>Mental illness 37257<br>Mental illnesses 6045<br>Anxiety 265049<br>Anxious 20603<br>Depress* 569151<br>Dysthymia 2320<br>Melancholy 426<br>Mood 92155<br>Affective disorder 7725<br>Affective symptoms 2417<br>Negative affect 13573<br>distress 154761<br>Emotional stress 5232<br><br>**MeSH terms**<br>Mental health 61933<br>Mental disorders 1436875<br>Anxiety 112111<br>Anxiety disorders 90630<br>Depression 257186<br>Depressive disorder 122190<br>Mood disorders 170032<br>Psychological Distress 6832	**All fields**<br>Ghana 24845<br><br>**MeSH term**<br>Ghana 11236
**Total for concept 1:**	**Total for concept 2:**
**2183845**	**25013**

Total for complete search: 1297

For peer review only

Raw PubMed Search

("mental health"[Title/Abstract] OR "mental disorders"[Title/Abstract] OR "mental disorder"[Title/Abstract] OR "mental illness"[Title/Abstract] OR "mental illnesses"[Title/Abstract] OR "anxiety"[Title/Abstract] OR "anxious"[Title/Abstract] OR "depress*"[Title/Abstract] OR "dysthymia"[Title/Abstract] OR "melancholy"[Title/Abstract] OR "mood"[Title/Abstract] OR "affective disorder"[Title/Abstract] OR "affective symptom"[Title/Abstract] OR "negative affect"[Title/Abstract] OR "distress"[Title/Abstract] OR "emotional stress"[Title/Abstract] OR ("mental health"[MeSH Terms] OR "mental disorders"[MeSH Terms] OR "anxiety"[MeSH Terms] OR "anxiety disorders"[MeSH Terms] OR ("depressive disorder"[MeSH Terms] OR "depression"[MeSH Terms]) OR "depressive disorder"[MeSH Terms] OR "mood disorders"[MeSH Terms] OR "psychological distress"[MeSH Terms])) AND ("Ghana"[MeSH Terms] OR "Ghana"[All Fields] OR "Ghana s"[All Fields] OR "Ghana"[MeSH Terms]).

For peer review only - http://bmjopen.bmj.com/site/about/guidelines.xhtml

PEER REVIEW ASSESSMENT: THIS SECTION TO BE FILLED IN BY THE REVIEWER

Reviewer: Professor Alkistis Skalkidou	Email: Alkistis.skalkidou@kbh.uu.se	Date completed: 14/9/2023

1. TRANSLATION

A ---No revisions	■
B --- Revision(s) suggested	☐
C --- Revision(s) required	☐

If "B" or "C," please provide an explanation or example:

Does the search strategy match the research question/PICO?
Are the search concepts clear?
Are there too many or too few PICO elements included?
Are the search concepts too narrow or too broad?
Does the search retrieve too many or too few records? (Please show number of hits per line.)
Are unconventional or complex strategies explained

For peer review only

2. BOOLEAN AND PROXIMITY OPERATORS

A ---No revisions	■
B --- Revision(s) suggested	☐
C --- Revision(s) required	☐

If "B" or "C," please provide an explanation or example:

Are Boolean or proximity operators used correctly?
Is the use of nesting with brackets appropriate and effective for the search?
If NOT is used, is this likely to result in any unintended exclusions?
Could precision be improved by using proximity operators (eg, adjacent, near, within) or phrase searching instead of AND?
Is the width of proximity operators suitable (eg, might adj5 pick up more variants than adj2)?
Not used

3. SUBJECT HEADINGS

A ---No revisions	☐
B --- Revision(s) suggested	■
C --- Revision(s) required	☐

If "B" or "C," please provide an explanation or example:

Are the subject headings relevant?<br>Are any subject headings too broad or too narrow?<br>Are subject headings exploded where necessary and vice versa?<br>Are major headings ("starring" or restrict to focus) used? If so, is there adequate justification?<br>Are subheadings missing?<br>Are subheadings attached to subject headings? (Floating subheadings may be preferred.)<br>Are floating subheadings relevant and used appropriately?<br>Are both subject headings and terms in free text (see the following) used for each concept?

4. TEXT WORD SEARCHING

A ---No revisions	■
B --- Revision(s)suggested	☐
C --- Revision(s) required	☐

If "B" or "C," please provide an explanation or example:

Does the search include all spelling variants in free text (eg, UK vs. US spelling)?<br>Does the search include all synonyms or antonyms (eg, opposites)?<br>Does the search capture relevant truncation (i.e., is truncation at the correct place)?<br>Is the truncation too broad or too narrow?<br>Are acronyms or abbreviations used appropriately? Do they capture irrelevant material? Are the full terms also included?<br>Have the appropriate fields been searched; for example, is the choice of the text word fields (.tw.) or all fields (.af.) appropriate? Are there any other fields to be included or excluded (database specific)?<br>Should any long strings be broken into several shorter search statements?

5. SPELLING, SYNTAX, AND LINE NUMBERS

A ---No revisions	■
B --- Revision(s)suggested	☐
C --- Revision(s) required	☐

If "B" or "C," please provide an explanation or example:

Are there any spelling errors?<br>Are there errors in system syntax (e.g. the use of a truncation symbol from a different search interface)?<br>Are there incorrect line combinations or orphan lines (e.g. lines that are not referred to in the final summation that?<br>could indicate an error in an AND or OR statement)?

6. LIMITS AND FILTERS

A ---No revisions	☐

For peer review only

B --- Revision(s) suggested	■
C --- Revision(s) required	□

If “B” or “C,” please provide an explanation or example:

Are all limits and filters used appropriately and are they relevant given the research question?

Are all limits and filters used appropriately and are they relevant for the database?

Are any potentially helpful limits or filters missing? Are the limits or filters too broad or too narrow? Can any limits

or filters be added or removed? Language? Time period? Only humans?

Are sources cited for the filters used?

Author response:

Thanks for the suggestion. Databases will be searched from inception, and the time period for data collection (e.g., <2020 vs. ≥2020) will be investigated as a methodological moderator of prevalence utilising subgroup analyses and meta-regression. Only human studies and language search filters were not used. However, non-human studies and studies published in languages other than English and Ghanaian would be excluded during the screening phase.

OVERALL EVALUATION (Note: If one or more “revision required” is noted above, the response below must be “revisions required”.)

A ---No revisions	□
B --- Revision(s) suggested	■
C --- Revision(s) required	□

Additional comments:

I am not familiar with Medline, but many within psychology also search the PSYCHINFO database

Author response:

Thank you for your feedback. MEDLINE (PubMed) is one of the electronic databases in which the searches will be conducted. However, electronic database searches will be also carried out in African Index Medicus (AIM), African Journals Online (AJOL), Cumulative Index to Nursing and Allied Health Literature (CINAHL), Excerpta Medica Database (Embase), Ghana Medical Journal (GMJ), Health Sciences Investigation (HIS), **PsycINFO** and SCOPUS.

For peer review only

PEER REVIEW ASSESSMENT #2: THIS SECTION TO BE FILLED IN BY THE REVIEWER

Reviewer: Associate Professor Lene Lindberg	Email: lene.lindberg@ki.se	Date completed:11/9/2023

1. TRANSLATION

A ---No revisions	☐
B --- Revision(s) suggested	■
C --- Revision(s) required	☐

If "B" or "C," please provide an explanation or example: See my questions above

Does the search strategy match the research question/PICO?
Are the search concepts clear?
Are there too many or too few PICO elements included?
Are the search concepts too narrow or too broad? Mental disorders is too broad?
Does the search retrieve too many or too few records? (Please show number of hits per line.)
Are unconventional or complex strategies explained

For peer review only

2. BOOLEAN AND PROXIMITY OPERATORS

A ---No revisions	■
B --- Revision(s) suggested	☐
C --- Revision(s) required	☐

If "B" or "C," please provide an explanation or example:

Are Boolean or proximity operators used correctly?
Is the use of nesting with brackets appropriate and effective for the search?
If NOT is used, is this likely to result in any unintended exclusions?
Could precision be improved by using proximity operators (eg, adjacent, near, within) or phrase searching instead of AND?
Is the width of proximity operators suitable (eg, might adj5 pick up more variants than adj2)?

3. SUBJECT HEADINGS

A ---No revisions	■
B --- Revision(s) suggested	☐
C --- Revision(s) required	☐

If "B" or "C," please provide an explanation or example: See my comment above

Are the subject headings relevant?
Are any subject headings too broad or too narrow?

Are subject headings exploded where necessary and vice versa?
Are major headings ("starring" or restrict to focus) used? If so, is there adequate justification?
Are subheadings missing?
Are subheadings attached to subject headings? (Floating subheadings may be preferred.)
Are floating subheadings relevant and used appropriately?
Are both subject headings and terms in free text (see the following) used for each concept?

4. TEXT WORD SEARCHING

A ---No revisions	■
B --- Revision(s)suggested	☐
C --- Revision(s) required	☐

If "B" or "C," please provide an explanation or example:

Does the search include all spelling variants in free text (eg, UK vs. US spelling)?
Does the search include all synonyms or antonyms (eg, opposites)?
Does the search capture relevant truncation (i.e., is truncation at the correct place)?
Is the truncation too broad or too narrow?
Are acronyms or abbreviations used appropriately? Do they capture irrelevant material? Are the full terms also included?
Have the appropriate fields been searched; for example, is the choice of the text word fields (.tw.) or all fields (.af.) appropriate? Are there any other fields to be included or excluded (database specific)?
Should any long strings be broken into several shorter search statements?

5. SPELLING, SYNTAX, AND LINE NUMBERS

A ---No revisions	■
B --- Revision(s)suggested	☐
C --- Revision(s) required	☐

If "B" or "C," please provide an explanation or example:

Are there any spelling errors?
Are there errors in system syntax (e.g. the use of a truncation symbol from a different search interface)?
Are there incorrect line combinations or orphan lines (e.g. lines that are not referred to in the final summation that?
could indicate an error in an AND or OR statement)?

6. LIMITS AND FILTERS

A ---No revisions	■
B --- Revision(s) suggested	☐
C --- Revision(s) required	☐

For peer review only

If "B" or "C," please provide an explanation or example:

Are all limits and filters used appropriately and are they relevant given the research question?<br>Are all limits and filters used appropriately and are they relevant for the database?<br>Are any potentially helpful limits or filters missing? Are the limits or filters too broad or too narrow? Can any limits<br>or filters be added or removed?<br>Are sources cited for the filters used?

OVERALL EVALUATION (Note: If one or more "revision required" is noted above, the response below must be "revisions required".)

A ---No revisions	■
B --- Revision(s) suggested	☐
C --- Revision(s) required	☐

Additional comments:

Any specific time period for the included studies?

Co-morbidity between depression and anxiety common, how will you treat data on that?

Author response:

Thanks for your feedback. Databases will be searched from inception. If data allows, we will explore sources of heterogeneity via subgroup analysis of the moderating effect of time period of data collection on prevalence (e.g., <2020 vs. ≥ 2020).

We will analyze prevalence estimates of anxiety, depression or a combined estimate of their symptom (i.e. psychological distress) separately. We could consider data on comorbidity between anxiety and depression and this would involve presenting the percentage of adults who experience both conditions simultaneously. Any protocol amendments will be recorded in PROSPERO.

For peer review only

Prevalence and social determinants of anxiety and depression among adults in Ghana: a systematic review and meta-analysis protocol

Supplementary Appendix 3: Search strategies-electronic databases

PubMed

#	Searches	Comment
1	Mental health [Title/Abstract] OR<br>Mental disorders [Title/Abstract] OR<br>Mental disorder [Title/Abstract] OR<br>Mental illness [Title/Abstract] OR<br>Mental illnesses [Title/Abstract] OR<br>Anxiety [Title/Abstract] OR<br>Anxious [Title/Abstract] OR<br>Depress*[Title/Abstract] OR<br>Dysthymia [Title/Abstract] OR<br>Melancholy [Title/Abstract] OR<br>Mood [Title/Abstract] OR<br>Affective disorder [Title/Abstract] OR<br>Affective symptoms [Title/Abstract] OR<br>Negative affect [Title/Abstract] OR<br>distress [Title/Abstract] OR<br>Emotional stress [Title/Abstract] OR	
2	Mental health [MeSH] OR<br>Mental disorders [MeSH] OR<br>Anxiety [MeSH] OR<br>Anxiety disorders [MeSH] OR<br>Depression [MeSH] OR<br>Mood disorders [MeSH] OR<br>Psychological Distress [MeSH] OR	
3	1 OR 2	
Ghana		
4	Ghana [All fields]	
5	Ghana [MeSH]	
6	4 OR 5	
Final results	3 AND 6	
syntax	(((((((((((((((Mental health[Title/Abstract]) OR (Mental disorders[Title/Abstract])) OR (mental disorder[Title/Abstract])) OR (mental illness[Title/Abstract])) OR (mental illnesses[Title/Abstract])) OR (anxiety[Title/Abstract])) OR (anxious[Title/Abstract])) OR (depress*[Title/Abstract])) OR (dysthymia[Title/Abstract])) OR	

	(melancholy[Title/Abstract])) OR (mood[Title/Abstract])) OR (affective disorder[Title/Abstract])) OR (affective symptoms[Title/Abstract])) OR (negative affect[Title/Abstract])) OR (distress[Title/Abstract])) OR (emotional stress[Title/Abstract])) OR ((((((((mental health[MeSH Terms]) OR (mental disorders[MeSH Terms])) OR (anxiety[MeSH Terms])) OR (anxiety disorders[MeSH Terms])) OR (depression[MeSH Terms])) OR (depressive disorder[MeSH Terms])) OR (mood disorders[MeSH Terms])) OR (psychological distress[MeSH Terms]))) AND ((Ghana) OR (Ghana[MeSH Terms]))

CINAHL

#	Searches	Comment
1	MW= (mental health OR mental disorders OR anxiety OR anxiety disorders OR depression OR mood disorders OR psychological Distress)	
2	TI= (mental health OR mental disorder*OR mental disorder OR mental illness OR mental illnesses OR anxiety OR anxious OR depress* OR dysthymia OR melancholy OR mood OR affective disorder OR affective symptoms OR negative affect OR distress OR emotional stress).	
3	AB= (mental health OR mental disorder*OR mental disorder OR mental illness OR mental illnesses OR anxiety OR anxious OR depress* OR dysthymia OR melancholy OR mood OR affective disorder OR affective symptoms OR negative affect OR distress OR emotional stress)	In Cinahl Title and Abstract are divided. Search 3 is for abstract OR between terms.
4	1 OR 2 OR 3	
5	MW=(Ghana)	Same as concept 1
6	TI=(Ghana)	
7	AB= (Ghana)	
8	5 OR 6 OR 7	
9 Final results	4 AND 8	
syntax	((MW (Mental health OR mental disorders OR anxiety OR anxiety disorders OR depression OR mood disorders OR psychological Distress) OR TI (Mental health OR mental disorder*OR mental disorder OR mental illness OR mental illnesses OR anxiety OR anxious OR depress* OR dysthymia OR melancholy OR mood OR affective disorder OR affective symptoms OR negative affect OR distress OR emotional stress) OR AB(Mental health OR mental disorder*OR mental disorder OR mental illness OR mental illnesses OR anxiety OR anxious OR depress* OR dysthymia OR melancholy	

For peer review only

	OR mood OR affective disorder OR affective symptoms OR negative affect OR distress OR emotional stress)) AND (MW(Ghana) OR TI(Ghana) OR AB(Ghana))

Embase

#	Searches	Comment
1	TI, AB= (mental health OR mental disorders OR mental disorder OR mental illness OR mental illnesses OR anxiety OR anxious OR depress* OR dysthymia OR melancholy OR mood OR affective disorder OR affective symptoms OR negative affect OR distress OR emotional stress).	
2	/ DE= (mental health OR mental disorders OR anxiety OR anxiety disorders OR depression OR mood disorders OR psychological distress)	Unexploded includes only a single term in hierarchy. [Emtree unexploded] =/de
3	1 OR 2	
4	“Ghana”/exp	
5	“Ghana”. ti, ab.	
6	4 OR 5	
9 Final results	3 AND 6	

PsycINFO

#	Searches	Comment
1	MA= (mental health OR mental disorders OR anxiety OR anxiety disorders OR depression OR mood disorders OR psychological distress)	
2	TI= (Mental health OR mental disorder OR mental disorder OR mental illness OR mental illnesses OR anxiety OR anxious	

	OR depress* OR dysthymia OR melancholy<br>OR mood OR affective disorder OR affective symptoms OR negative affect OR distress OR emotional stress)	
3	AB= (Mental health OR mental disorder OR mental disorder OR mental illness OR mental illnesses OR anxiety OR anxious OR depress* OR dysthymia OR melancholy<br>OR mood OR affective disorder OR affective symptoms OR negative affect OR distress OR emotional stress)	OR between terms.
4	1 OR 2 OR 3	
5	MA= (Ghana)	Same as concept 1
6	TI= (Ghana)	
7	AB= (Ghana)	
8	5 OR 6 OR 7	
9 Final results	4 AND 8	

Scopus

#	**Searches**
1	TITLE-ABS-KEY (mental health OR mental disorder*OR mental illness*OR anxiety disorders OR anxiety OR anxious OR dysthymia<br>OR melancholy OR mood OR affective disorder OR affective symptoms OR negative affect OR distress OR emotional stress OR depress*OR mood disorders OR psychological Distress)
2	TITLE-ABS-KEY(Ghana)
3 Final results	1 AND 2

For peer review only

African Index Medicus (AIM)

#	Searches	Comment
1	TITLE-ABS-SUB (mental health OR mental disorders OR mental disorder OR mental illness OR mental illnesses OR anxiety OR anxious OR depress* OR dysthymia OR melancholy OR mood OR affective disorder OR affective symptoms OR negative affect OR distress OR emotional stress)	Due to no Search history a simplified strategy was employed: In one go, using title, abstract, subject field.
2	TITLE-ABS-SUB (Ghana)	
3 Final results	1 AND 2	

African journals online (AJOL)

#	Searches	Comment
1	Ghana AND ("mental disorders" OR "mental health" OR "anxiety" OR "anxiety disorders" OR "depression" OR "depressive symptoms" OR "psychological distress" OR “mental distress”) site:ajol.info	AJOL has a google search engine. We used Google scholar interface, incognito mode to minimize effect of Google algorithm. The search yields the same results without incognito mode. Cut-off at 5(50 studies), due to fluctuating result from page 6 onwards.

Health Science Investigation (HIS)

#	Searches	Comment
1	(mental disorders OR mental health OR anxiety OR anxiety disorders OR depression OR depressive symptoms OR psychological distress OR mental distress) AND Ghana	HIS has two advanced filters: Published After (2020,2021,2022 or 2023) and Published before (2020, 2021,2022 or 2023). The search yielded no result when no filter was applied. However,

For peer review only

		when, for instance, 2020 was selected for published after and 2023 for published before, the search yielded results. Note: The search did not yield any results when the inverted commas were added to the searches.

Ghana Medical Journal (GMJ)

#	Searches	Comment
1	(mental disorders OR mental health OR anxiety OR anxiety disorders OR depression OR depressive symptoms OR psychological distress OR mental distress) AND Ghana site:ghanamedj.org	GMJ is indexed in AJOL and since both sites use google interface, we searched in Google scholar interface, incognito mode to minimize effect of Google algorithm. The search yields the same results without incognito mode.

For peer review only

Prevalence and social determinants of anxiety and depression among adults in Ghana: a systematic review and meta-analysis protocol

Supplementary Appendix 4: Data extraction form

Study Identification Features	
Study ID/ record number	
Title and aim	
First author last name	
Year of publication	
Publication type or Source of data (e.g., journal or report)	
**Study Characteristics**	
Age (M, SD) and range	
Gender	
Socioeconomic status	
Chronic physical condition reported (e.g., cancer, diabetes, HIV/AIDs)	
Sample size	
Sampling methods (e.g., convenience sampling, random sampling)	
Geographic region	
Geographical location (rural, urban mixed)	
Recruitment setting (Community or clinical settings	
Study design (e.g., cohort study, cross-sectional study)	
Time period of data collection	
Type of mental health condition(s) assessed (i.e. anxiety, depression, and psychological distress)	
Structured diagnostic clinical interview	Yes/No
Name of diagnostic clinical interview	
Screening tool	Yes/No
Type of screening tool (self- report, clinician or proxy administered),	
Name of screening tool and cutoff scores	
Evidence of validity of the measurement instrument	
Evidence of ethical approval	
**Result Summary**	
**Results Summary Prevalence[a]**	
Binary prevalence data (n/N) and percentage with 95% confidence intervals	
Prevalence type (current, period or period or life time)	

For peer review only

Social determinants summary				
Domains of the Social determinants	Proximal factors identified		Distal factors identified	
	Risk factors	Protective factors	Risk factors	Protective factors
Demographic domain				
Economic domain				
Environmental domain				
Neighbourhood domain				
Social and cultural domain				
Reviewer comments, if any?				

[a]Prevalence data will be extracted for all conditions, namely, depression, anxiety and psychological distress

BMJ Open

Prevalence and social determinants of anxiety and depression among adults in Ghana: a systematic review and meta-analysis protocol

Journal:	*BMJ Open*
Manuscript ID	bmjopen-2023-081927.R2
Article Type:	Protocol
Date Submitted by the Author:	09-Apr-2024
Complete List of Authors:	Awortwe, Victoria; Uppsala University, Healthcare Sciences and e-Health, Department of Women's and Children's Health<br>Daivadanam, Meena ; Uppsala University, Global Health and Migration Unit, Department of Women's and Children's Health<br>Adjorlolo, Samuel ; University of Ghana, Department of Mental Health, School of Nursing & Midwivery, College of Health Sciences<br>Olsson, Erik; Uppsala University, Healthcare Sciences and e-Health, Department of Women's and Children's Health<br>Coumoundouros, Chelsea; Uppsala University, Healthcare Sciences and e-Health, Department of Women's and Children's Health<br>Woodford, Joanne; Uppsala University, Healthcare Sciences and e-Health, Department of Women's and Children's Health
**Primary Subject Heading**:	Mental health
Secondary Subject Heading:	Epidemiology, Public health, Global health
Keywords:	Adult psychiatry < PSYCHIATRY, EPIDEMIOLOGY, MENTAL HEALTH, PUBLIC HEALTH, Systematic Review

SCHOLARONE™
Manuscripts

Prevalence and social determinants of anxiety and depression among adults in Ghana: a systematic review and meta-analysis protocol

Victoria AWORTWE[1] (victoria.awortwe@uu.se), Meena DAIVADANAM[2] (meena.daivadanam@kbh.uu.se), Samuel ADJORLOLO[3] (sadjorlolo@ug.edu.gh), Erik OLSSON[1] (erik.olsson@kbh.uu.se), Chelsea COUMOUNDOUROS[1]* (chelsea.coumoundouros@kbh.uu.se), Joanne WOODFORD[1]* (joanne.woodford@kbh.uu.se)

*Joint last authorship

[1]Healthcare Sciences and e-Health, Department of Women's and Children's Health, Uppsala University, Uppsala, Sweden.

[2]Global Health and Migration Unit, Department of Women's and Children's Health, Uppsala University, Uppsala, Sweden.

[3]Department of Mental Health, School of Nursing and Midwifery, College of Health Sciences, University of Ghana, Accra, Ghana.

Corresponding author

Victoria Awortwe

For peer review only

ABSTRACT

Introduction: Anxiety and depression pose a significant global health challenge, especially affecting adults in low-and middle-income countries. In many low and middle-income countries, including those in sub-Saharan Africa, social determinants such as access to affordable health services, conflict, food insecurity, and poverty may be associated with the prevalence of anxiety and depression, further contributing to health disparities. To mitigate the burden of anxiety and depression in sub-Saharan Africa, it is essential to develop country-level tailored mental health policies and strategies. For example, Ghana is working towards improving mental health via its 12-year Mental Health policy launched in 2021. However, the prevalence of anxiety and depression among adults in Ghana, along with associated social determinants remains largely unknown, posing challenges for mental health planning, resource allocation, and developing targeted interventions. This review aims to examine the prevalence of anxiety and depression among adults in Ghana, and explore social determinants potentially associated with anxiety and depression.

Methods and analysis: Electronic databases (e.g., African Index Medicus, CINAHL, EMBASE, MEDLINE, PsycINFO) will be searched with all screening steps conducted by two independent reviewers. Secondary search strategies, including grey literature searches, will be used. Studies reporting on the prevalence of anxiety, depression, and/or a combined symptom measure (i.e. psychological distress) among adults in Ghana, using validated instruments will be included. If data allows, random-effects-meta-analyses will be performed to estimate pooled prevalence rates of anxiety and depression. Potential clinical and methodological moderators will be examined using subgroup analyses and meta-regression. A narrative synthesis will explore social determinants potentially associated with anxiety and depression among adults in Ghana.

Ethics and dissemination: Ethical approval is not required as no primary data will be collected. Results will be disseminated via a peer-reviewed publication and presentations at academic conferences. Plain language summaries will be provided to relevant non-governmental organizations working in Ghana.

PROSPERO registration number: CRD42023463078

For peer review only - http://bmjopen.bmj.com/site/about/guidelines.xhtml

Strengths and limitations of this study

- This review, to our knowledge, is the first of its kind, aiming to examine the prevalence of anxiety and depression among adults in Ghana and explore the social determinants potentially associated with anxiety and depression.
- The review protocol adheres to quality standards informed by the Preferred Reporting Items for Systematic Reviews and Meta-Analysis Protocols (PRISMA-P) checklist, with all screening steps and quality appraisal conducted by two independent reviewers.
- The peer-reviewed, comprehensive search strategy will ensure the inclusion of a wide range of relevant studies, reducing the risk of selection bias.
- High levels of heterogeneity across studies due to differences in populations, condition measurements and study designs may limit the possibility of conducting a meta-analysis.
- Qualitative studies will be excluded therefore limiting an in-depth exploration of the perspectives of adults in Ghana on social determinants associated with their mental health, which may have provided important information to inform future intervention development.

For peer review only - http://bmjopen.bmj.com/site/about/guidelines.xhtml

INTRODUCTON

Common mental disorders, such as anxiety and depression are among the most prevalent and rising health problems affecting adults worldwide.[1, 2] Anxiety and depression are leading causes of health-related burden globally, with depression being the second and anxiety being the eighth leading cause of years lived with disability (YLDs) out of 369 diseases and injuries in 204 countries and territories.[1] The consequences of anxiety and depression can be severe; resulting in impaired social functioning, increased mortality rates, low productivity, and reduced quality of life.[3] Research suggests anxiety and depression are associated with an increased risk of developing complications or worsening a range of chronic physical conditions including cancer, diabetes, heart disease, and human immunodeficiency virus (HIV).[4–6] The economic burden of anxiety and depression is also substantial with costs stemming from healthcare service use, lost productivity, and impact on families, caregivers, and wider society.[7, 8] The burden and impact of anxiety and depression on individuals and societies underscores the critical need to prioritize adult mental health and well-being globally.

The majority of the global burden of mental disorders is located in low-and middle-income countries (LMICs),[9] where 75% of individuals experiencing common mental disorders do not receive appropriate mental healthcare.[10] This treatment gap is particularly pronounced in Africa owing to the limited availability of mental health resources, coupled with healthcare systems that are inadequately equipped to meet the needs of individuals seeking care.[11, 12] A previous scoping review conducted in 12 African countries found a lifetime prevalence rate of anxiety ranging from 5.7% to 15.8% and depression ranging from 3.3% to 9.8%.[13] Within Africa, the prevalence of mental disorders including anxiety and depression is expected to increase by 130% between 2010 and 2050, potentially leading to 45 million YLDs in sub-Saharan Africa (SSA),[14] with major depression predicted to be the largest contributor to disease burden in SSA.[14] Therefore, researchers, global health entities, and advocacy groups are urging actions to address the growing mental health crisis in SSA,[15] advocating for integrating mental health into primary care, establishing national policies, training paraprofessionals, expanding community healthcare, and collaborating with traditional healers.[14, 16] To further inform these actions,[15] there is a need to better understand the determinants of mental health that may contribute to the development and exacerbation of mental disorders in SSA, particularly in resource-constrained countries in the region.

For peer review only - http://bmjopen.bmj.com/site/about/guidelines.xhtml

Determinants of mental health among adults are multifaceted, and can be broadly categorized into biological (e.g., genetics, brain chemistry, and hormonal imbalances), psychological (e.g., cognitive and interpersonal factors), and social (e.g., ethnicity, food security, neighborhood deprivation).[17] Social determinants can also exacerbate biological and psychological vulnerabilities via mechanisms such as gene-environment interactions, epigenetic modifications, and maladaptive coping.[18–20] Globally, social determinants are recognized for their role in either providing protection or increasing the risk of adult mental disorders such as anxiety and depression, while also contributing to health disparities.[21–23] Within LMICs, including those in Africa, social determinants including diminished social capital, environmental events, food insecurity, forced migration, income inequality, violence, low education, poor housing, poverty, and unemployment,[24–27] may increase vulnerability to experiencing mental health difficulties.[15] Ethnicity as a social determinant also requires additional consideration given experiences of discrimination and exclusion, as a result of ethnicity, impact mental health.[22] Furthermore, while ethnicity has been established as a social determinant of mental health, the interactions of mechanisms such as norms, differences in cultural interpretations of symptoms and practices can also impact mental health.[21, 22] Recognizing the over-arching importance of social determinants, the World Health Organization (WHO) has urged focus on reshaping economic, physical, and social factors to improve mental health and reduce inequalities by accelerating the implementation of the Comprehensive Mental Health Action Plan 2013-2030.[28] Importantly, the majority of social determinants such as poor living arrangements and low or worsening socioeconomic status are modifiable,[29] and may be improved by implementing appropriate governmental policies and adopting a multi-sectoral approach to provide comprehensive mental health and social care services.[7, 30] In SSA, there is a growing consensus that mental health policies and services should be tailored to the contextual realities of each country in the region, with evidence emphasizing the importance of documenting the social determinants specific to each SSA country where mental disorders are experienced.[15, 31]

The focus of the present review is Ghana, a LMIC in SSA with a population of approximately 31 million, with an estimated 13% of adults experiencing mental disorders including anxiety and depression.[32] Despite this, only 2% receive treatment.[33] Chronic physical conditions like cancer, diabetes, and HIV are commonly linked to mental disorders among adults in Ghana[34, 35] and the burden of anxiety and depression in Ghana is further worsened by inadequate mental health resources and healthcare disparities.[35] Social determinants such as

For peer review only - http://bmjopen.bmj.com/site/about/guidelines.xhtml

crime, food insecurity, poor sanitation, poverty and unemployment may also contribute to elevated rates of mental disorders in adults in Ghana.[35–38] To our knowledge, there has been no systematic review undertaken to consolidate existing research, examine the prevalence rates of anxiety and depression among adults; and explore key social determinants specific to the Ghanaian context. By examining the prevalence of anxiety and depression among adults in Ghana and exploring social determinants potentially associated with anxiety and depression, this review will provide insights into the magnitude of the problem in Ghana, and social determinants influencing their occurrence to inform evidence-based policies and interventions aimed at enhancing mental well-being and overall health of adults in Ghana. The review's findings will also be valuable for countries experiencing similar social determinants as the Ghanaian population.

Research Objectives

This systematic review seeks to: (1) examine the prevalence of anxiety, depression, and psychological distress among adults in Ghana and (2) explore the social determinants potentially associated with anxiety, depression and psychological distress.

METHODS

This review protocol adheres to the Preferred Reporting Items for Systematic Reviews and Meta-Analysis Protocols (PRISMA-P) checklist[39] (online supplementary appendix 1). The Joanna Briggs Institute (JBI) methodology for systematic reviews on prevalence was used to guide protocol development.[40] The protocol has been registered in the International Prospective Register of Systematic Reviews (PROSPERO, registration number CRD42023463078). The study is scheduled to commence on September 25, 2023, with an anticipated completion date of September 1, 2024. Any protocol amendments will be recorded in PROSPERO.

Eligibility criteria

Eligibility criteria was developed and defined in accordance with the CoCoPop (Condition, Context, and Population) and type of studies framework.[41]

For peer review only - http://bmjopen.bmj.com/site/about/guidelines.xhtml

Population

Adults (aged ≥ 18 years) living in Ghana will be eligible for inclusion. Studies solely focused on children/adolescents will be excluded. Studies focusing on both adults and adolescents will be excluded if they do not present data separately for adults and adolescents or data cannot be obtained via correspondence with study authors. Studies conducted with specific sub-populations including individuals with known psychiatric conditions, prisoners, individuals accused of witchcraft, and women with fertility or gynaecological disorders, will be excluded due to their unique circumstances, which predispose them to a higher risk or potentially elevate the likelihood of experiencing mental health problems compared to the general population.

Condition

Conditions eligible for inclusion include anxiety and / or depression assessed using a structured diagnostic clinical interview in accordance with the International Classification of Diseases and Related Health Problems (ICD-10 or ICD-11), or the Diagnostic and Statistical Manual of Mental disorders (DSM), third, fourth or fifth edition (DSM-III, DSM-IV or DSM-V) such as the Structured Clinical Interview for (SCID), the Mini-International Neuropsychiatric Interview (MINI), or the Composite International Diagnostic Interview (CIDI). Studies using a self-report, clinician or proxy administered screening tool for anxiety (e.g., Beck Anxiety Inventory), depression (e.g., Beck Depression Inventory), or psychological distress (e.g., Kessler Psychological Distress Scale) will also be eligible for inclusion. Studies reporting point (current), period (timepoint), or lifetime estimates of the prevalence of anxiety, depression and psychological distress will be included.[42] To ensure the reliability and quality of assessment methods, only studies using instruments validated for use in adult populations will be included. Validity will be assessed based on evidence provided in the validation paper(s) of the measurement instrument and/or evidence of psychometric properties such as construct validity, content validity, criterion validity, and reliability measures including internal consistency, test-retest reliability, and inter-rater reliability. Studies using instruments validated for use in adult samples in Ghana will also be eligible for inclusion. Studies solely focused on other mental disorders (e.g., psychotic disorders, bipolar affective disorders), substance use and neurological disorders (e.g., alcohol dependence, multiple sclerosis) will be excluded. Studies will be excluded if the prevalence of anxiety, depression and psychological distress cannot be calculated, e.g., when reported solely as mean score or due to insufficient data.

For peer review only - http://bmjopen.bmj.com/site/about/guidelines.xhtml

For peer review only

Context

This review will include studies conducted in Ghana with adults sampled from the community or clinical settings (e.g., primary healthcare facilities, hospitals). Studies conducted in regions that encompass Ghana (e.g., West Africa and Sub-Saharan Africa) will be considered eligible if data on participants living in Ghana can be extracted from the publication or obtained via correspondence with study authors. Studies conducted immediately after conflict (i.e., less than four months after the official end date of the conflict),[43] humanitarian crises, or natural disaster will be excluded given we seek to understand the general prevalence of anxiety and depression.[44]

Types of studies

Primary quantitative studies, with observational study designs including longitudinal cohort studies (baseline data only), case-control, and cross-sectional studies reporting the prevalence of anxiety, depression and/or psychological distress among adults in Ghana will be included. Mixed method studies will be eligible for inclusion only if data from the quantitative component can be clearly extracted. In the case of studies conducted on the same cohort of individuals at the same or different points in time; or where samples overlap, only the study with the largest sample and findings related to the aims of this review will be included to ensure duplicate data is not included. Studies such as case reports, commentaries, conference proceedings, editorials, letters, opinion papers, qualitative studies, reviews, theses/dissertations will be excluded.

Information sources

Searches will be conducted in accordance with PRISMA 2020 guidelines.[45] Electronic database searches will be carried out in African Index Medicus (AIM), African Journals Online (AJOL), Cumulative Index to Nursing and Allied Health Literature (CINAHL), Excerpta Medica Database (Embase), Ghana Medical Journal (GMJ), Health Sciences Investigation (HIS), MEDLINE (PubMed), PsycINFO and SCOPUS. Electronic databases will be searched from inception up to September 25, 2023, and updated study searches will be conducted within 3 months to submitting the results manuscript. Reference lists of included studies will be manually checked and forward citation checks of included studies will be performed. Reference lists of relevant systematic reviews conducted in sub-Saharan Africa, including Ghana will also

For peer review only - http://bmjopen.bmj.com/site/about/guidelines.xhtml

For peer review only

be manually checked. Grey literature will be searched in Agency for Healthcare Research and Quality (AHRQ), Google Scholar, Health Systems Trust, Open Grey (http://www. opengrey.eu/) and the World Health Organization (WHO). Researchers and non-governmental organizations working in the area of adult mental health in Ghana will be contacted to identify unpublished or ongoing studies.

Search strategy

The search strategy has been developed in collaboration with Mattias Axén, a librarian at Uppsala University Library and was reviewed by Alkistis Skalkidou and Lene Lindberg, following the PRESS Peer Review guidelines[46] (online supplementary appendix 2). The search was constructed using terms related to (1) mental disorders and (2) Ghana (see online supplementary appendix 3). Electronic databases will be searched using Medical Subject Headings (MeSH) when possible and free text words in title and abstract word searches. No date restriction will be imposed and only studies published in English and Ghanaian languages (e.g., Fante, Ga, Twi) will be considered for inclusion.

Study selection

Studies retrieved from searches will be uploaded into Endnote 20 with duplicates identified and removed. Two reviewers will independently screen titles and abstracts in Rayyan,[47] followed by full paper checks of potentially eligible studies. Studies not meeting the eligibility criteria will be excluded. Overall reasons for exclusion will be documented and reported using the PRISMA flow chart and detailed reasons for exclusion, in accordance with the eligibility criteria, will be presented in a table. If study data/information required to determine eligibility is missing, authors will be contacted via email for additional information. Any disagreement between reviewers will be resolved by discussion and/ or involvement of a third reviewer.

Data extraction

Data from included studies (see online supplementary appendix 4), will be extracted by one reviewer independently into a standardized Microsoft Excel data extraction form, and crosschecked by a second reviewer for accuracy. The following data will be extracted:

For peer review only - http://bmjopen.bmj.com/site/about/guidelines.xhtml

1. Study identification features: study ID/record number, study title and aim, first author name, year of publication, and publication type or data source (e.g., journal, or report).
2. Study characteristics: characteristics of study population (age, gender, socioeconomic status, chronic physical condition reported), sample size, sampling methods (e.g., convenience sampling, random sampling), study setting including geographical region(s) in Ghana where study was conducted, location (rural, urban, mixed), and recruitment setting (community or clinical settings), study design (e.g., case-control, cross-sectional, longitudinal cohort studies), time period of data collection, type of mental health condition (anxiety, depression and psychological distress), structured diagnostic clinical interview (yes/no), name of diagnostic clinical interview, screening tool (yes/no), name of screening tool and cutoff scores, evidence of validity of the measurement instrument, as well as evidence of ethical approval.
3. Results summary: binary prevalence data of anxiety, depression and psychological distress (i.e. number of cases/ the total sample size, n/N) and percentage with 95% confidence intervals, prevalence type (current, period or life time) and summary of social determinants classified into demographic, economic, environmental, neighbourhood, social and cultural domains.

Quality assessment

The methodological quality of all included studies will be independently assessed by two reviewers using the standardized JBI Critical Appraisal Tool for Prevalence Studies.[48] The JBI Critical Appraisal Tool for Prevalence Studies comprises nine items including: appropriateness of sampling frame and sampling technique, adequacy of sample size, coverage of identified sample, description of study setting and subjects, validity of condition identification methods, standard and reliable measurement of condition, statistical analysis, and adequacy of response rate to assess the methodological quality and/or risk of bias of sampling, analysis and measurements in primary studies. The total score ranges from 1 to 9 for individual studies, [49] with the total number of "yes" scores for individual studies averaged to appraise studies as low ($\leq$ 3 score), moderate (4-6 points) or high quality ($\geq$ 7 points).[50] Any disagreement between reviewers will be resolved by discussion and / or involvement of a third reviewer.

For peer review only - http://bmjopen.bmj.com/site/about/guidelines.xhtml

Data analysis and synthesis

Quantitative data synthesis

If data allows, a meta-analysis with prevalence data from eligible studies will be conducted using Comprehensive Meta-Analysis software.[51] Data on the proportion of adults with anxiety, depression and psychological distress and their respective sample size will be extracted separately from individual studies to generate pooled estimates with exact binomial test and associated 95% confidence intervals. Data will be transformed to their logits before meta-analysis to stabilize variances.[52] Due to expected heterogeneity in individual studies, the random-effect model will be used to generate pooled prevalence estimates for anxiety, depression, and psychological distress respectively.[51] Heterogeneity across studies will be estimated using Cochran's Q statistic. The I^2 statistics will be used to measure the proportion of total variability due to between-study heterogeneity. The prediction interval (T^2) will be used as an estimate of between-study variance in true effects observed in eligible studies. Sensitivity analyses will be performed to explore the impact of individual studies on the overall prevalence estimate of anxiety and depression. This will be conducted for example, by removing studies conducted during COVID or studies of lower quality individually from the overall analysis to ascertain if their removal causes any substantial change to overall prevalence estimates. Egger's regression statistic and funnel plots will be used to assess the presence of any publication bias. In the case of significant publication bias,[53] the trim & fill method will be used to identify and correct the asymmetry of the funnel plot to yield a corrected pooled prevalence.

Subgroup analysis

If data allows, we will explore sources of heterogeneity via subgroup analysis of the moderating effects of the following factors on prevalence:

- Chronic physical conditions (e.g., long-term life-threatening conditions such as cancer, diabetes, emphysema, hypertension, HIV/AIDS, ischemic heart disease, and stroke, and chronic conditions such as arthritis, asthma, back problems of any kind, chronic bronchitis, gall bladder diseases, joint pain, osteoporosis and stomach ulcers).[54]
- Method of mental health assessment (e.g., structured clinical interview or screening tool).
- Sample size (e.g., <100 vs. ≥100).

For peer review only - http://bmjopen.bmj.com/site/about/guidelines.xhtml

- Study design (e.g., case-control study, cross-sectional studies, longitudinal cohort study).
- Study quality (low, medium or high-quality studies).
- Prevalence type (point, period, lifetime).
- Time period of data collection (e.g., <2013 vs. ≥2013 or <2020 vs. ≥2020).

Narrative synthesis

Social determinants will be narratively synthesized, with specific distal and/or proximal factors grouped under domains of the social determinants of mental disorders and Sustainable Development Goals (SDGs) framework,[22] outlined below:

1. Demographics: proximal factors (e.g., age, ethnicity, gender) and distal factors (e.g., community diversity, population density).

2. Economic: proximal factors (e.g., assets, debt, unemployment) and distal factors (e.g., economic inequality, recessions).

3. Neighbourhood: proximal factors (e.g., safety and security, housing structure, overcrowding) and distal factors (e.g., neighbourhood deprivation, built environment).

4. Environmental events: proximal factors (e.g., trauma) and distal factors (climate change, forced migration, war or conflict).

5. Social and cultural: proximal factors (e.g., individual social capital, social participation, education) and distal factors (e.g., social stability, community social capital).

Patient and public involvement

Public contributors were not involved in the development of this protocol and will not be involved in the conduct of the review due to time and resource limitations. We will seek to involve public contributors in co-writing plain language summaries to be provided to non-governmental organizations working in the area of adult mental health in Ghana**.**

For peer review only - http://bmjopen.bmj.com/site/about/guidelines.xhtml

DISCUSSION

To the best of our knowledge, there is currently no comprehensive review examining the prevalence and social determinants of anxiety and depression among adults in Ghana. This review will extend existing epidemiology literature on mental health[36, 55, 56] by providing a comprehensive summary of prevalence estimates of anxiety, depression and psychological distress among adults in Ghana, as well as social determinants potentially influencing their occurrence. The review has a number of strengths including a peer-reviewed, comprehensive search strategy ensuring the inclusion of a wide range of relevant studies, and reducing the risk of selection bias. By considering comorbidities, the review recognizes that mental health difficulties often co-occur with physical conditions, contributing to a better understanding of the overall health and mental well-being of adults in Ghana. Comorbidity between anxiety and depression is frequent and if data permits, the prevalence of comorbid anxiety and depression will be documented. The review protocol also adheres to quality standards informed by the PRISMA-P checklist,[39] with screening, selection, and quality appraisal assessed by two independent reviewers.

While there are several strengths, there are also limitations. Qualitative studies will be excluded, limiting an in-depth exploration of the perspectives of adults in Ghana on social determinants associated with their mental health, which may have provided important information to inform mental health intervention development. High levels of clinical and methodological heterogeneity across studies due to factors such as differences in populations, condition measurements and study design may limit the possibility of conducting a meta-analysis. This situation may warrant the need for narrative synthesis.

Despite these limitations, the significance of this review is highlighted by the widespread global attention on mental health, with initiatives such as the WHO Comprehensive Mental Health Action Plan 2013-2030 and the United Nation's Sustainable Developmental Goals (SDGs) highlighting the importance of mental healthcare equity, prevention, treatment, and promotion.[57] Whilst Ghana is working towards improving mental health via its revised 12-year Mental Health Policy launched in 2021,[35] a comprehensive review examining the prevalence of anxiety and depression among adults in Ghana can facilitate the planning and allocation of resources for mental healthcare.[35] Addressing the social determinants is also crucial for achieving broader development targets, such as SDG 1 for no poverty, SDG 5 for gender equality, and SDG 4 for quality education, among others.[22] Findings may be used to

For peer review only - http://bmjopen.bmj.com/site/about/guidelines.xhtml

inform future research, mental health-care planning, and the development of culturally responsive interventions aimed at improving the mental well-being and overall health of adults in Ghana.

ETHICS AND DISSEMINATION

Findings will be disseminated through scientific publication in a peer-reviewed journal and as presentations at conferences. The authors will use scientific presentations at institutional events, regional and international conferences and social media to promote the review result. Plain language summaries will be provided to non-governmental organizations working in the area of adult mental health in Ghana.

Acknowledgments Alkistis Skalkidou (AS) from the Department of Women's and Children's Health at Uppsala University and Lene Lindberg (LL) from the Department of Public Health Sciences at Karolinska Institute for providing peer review of the search strategy. Mattias Axén (MA), a Librarian at Uppsala University Library for assisting with the development of the electronic search strategy. Febrina Maharani (FM), master student in Global Health at Uppsala University for assisting with study selection process.

Contributors: VA, MD and JW conceptualized the study. VA drafted the proposal. VA, CC and JW designed the study. All authors (VA, MD, EO, SA, CC and JW) assisted with manuscript writing and critical revision of the study design and manuscript. All authors read and approved the final manuscript. JW is the guarantor of the review.

Funding This work was supported by U-CARE, which is a strategic research environment funded by the Swedish Research Council (dnr 2009–1093).

Disclaimer This funding source had no role in the design of this study and will not have any role during its execution, analyses, interpretation or the data or decision to submit results.

Competing interests None declared.

Patient consent for publication Not required.

Ethics approval This study protocol is for a systematic review, meta- analysis and meta synthesis using secondary data. No raw individual level data is included within this review, with only group level data extracted and analysed, as such no formal ethical approval is required.

Provenance and peer review Not commissioned; externally peer reviewed.

For peer review only - http://bmjopen.bmj.com/site/about/guidelines.xhtml

Data availability statement Not applicable.

References

1. GBD 2019 Mental Disorders Collaborators. Global, regional, and national burden of 12 mental disorders in 204 countries and territories, 1990-2019: a systematic analysis for the Global Burden of Disease Study 2019. Lancet Psychiatry. 2022;9(2):137–50.

2. Hyman S, Parikh R, Collins PY, Patel V. Adult Mental Disorders. In: Mental, Neurological, and Substance Use Disorders: Disease Control Priorities, Third Edition (Volume 4) [Internet]. The International Bank for Reconstruction and Development / The World Bank; 2016 [cited 2023 Apr 28]. Available from: https://www.ncbi.nlm.nih.gov/books/NBK361952/

3. Ormel J, VonKorff M. Reducing common mental disorder prevalence in populations. JAMA Psychiatry. 2021;78(4):359–60.

4. Daré LO, Bruand PE, Gérard D, Marin B, Lameyre V, Boumédiène F, et al. Co-morbidities of mental disorders and chronic physical diseases in developing and emerging countries: a meta-analysis. BMC Public Health. 2019;19(1):304.

5. Scott KM, Lim C, Al-Hamzawi A, Alonso J, Bruffaerts R, Caldas-de-Almeida JM, et al. Association of mental disorders with subsequent chronic physical conditions: World Mental Health Surveys from 17 countries. JAMA Psychiatry. 2016;73(2):150–8.

6. World Health Organisation. Comprehensive Mental Health Action Plan 2013-2030 [Internet]. 2021 [cited 2022 Dec 28]. Available from: https://www.who.int/publications-detail-redirect/9789240031029

7. World Health Organization. Comprehensive Mental Health Action Plan 2013-2030 [Internet]. Geneva: World Health Organization; 2021 [cited 2023 May 29]. Available from: https://www.who.int/publications-detail-redirect/9789240031029

8. World Health Organization and Calouste Gulbenkian Foundation. Social determinants of mental health [Internet]. World Health Organization, Geneva; 2014 [cited 2023 Apr 28]. Available from: https://www.who.int/publications-detail-redirect/9789241506809

9. World Health Organization. Depression and Other Common Mental Disorders: Global Health Estimates [Internet]. Geneva; 2017 [cited 2023 Mar 15]. Available from: https://www.who.int/publications-detail-redirect/depression-global-health-estimates

10. Dua T, Barbui C, Clark N, Fleischmann A, Poznyak V, Ommeren M van, et al. Evidence-based guidelines for mental, neurological, and substance use disorders in low- and middle-income countries: summary of WHO recommendations. PLOS Medicine. 2011;8(11): e1001122.

11. Lund C, Alem A, Schneider M, Hanlon C, Ahrens J, Bandawe C, et al. Generating evidence to narrow the treatment gap for mental disorders in sub-Saharan Africa: rationale, overview and methods of AFFIRM. Epidemiol Psychiatr Sci. 2015;24(3):233–40.

For peer review only - http://bmjopen.bmj.com/site/about/guidelines.xhtml

12. Sankoh O, Sevalie S, Weston M. Mental health in Africa. Lancet Glob Health. 2018;6(9): e954–5.

13. Greene MC, Yangchen T, Lehner T, Sullivan PF, Pato CN, McIntosh A, et al. The epidemiology of psychiatric disorders in Africa: a scoping review. Lancet Psychiatry. 2021;8(8):717–31.

14. Charlson FJ, Diminic S, Lund C, Degenhardt L, Whiteford HA. Mental and substance use disorders in Sub-Saharan Africa: predictions of epidemiological changes and mental health workforce requirements for the next 40 years. PLOS ONE. 2014;9(10): e110208.

15. Monteiro N. Addressing mental illness in Africa: global health challenges and local opportunities. Community Psychol Glob Perspect. 2015; 1:78–95.

16. Atilola O. Cross-cultural child and adolescent psychiatry research in developing countries. Glob Ment Health. 2015;2: e5.

17. Patel V, Saxena S, Lund C, Thornicroft G, Baingana F, Bolton P, et al. The Lancet Commission on global mental health and sustainable development. Lancet. 2018;392(10157):1553–98.

18. Compton MT, Shim RS. The social determinants of mental health. FOC. 2015 Oct;13(4):419–25.

19. Gottschalk MG, Domschke K. Novel developments in genetic and epigenetic mechanisms of anxiety. Curr Opin Psychiatry. 2016;29(1):32–8.

20. Remes O, Mendes JF, Templeton P. Biological, psychological, and social determinants of depression: a review of recent literature. Brain Sci. 2021;11(12):1633.

21. Alegría M, NeMoyer A, Falgàs Bagué I, Wang Y, Alvarez K. Social determinants of mental health: where we are and where we need to go. Curr Psychiatry Rep. 2018;20(11):95.

22. Lund C, Brooke-Sumner C, Baingana F, Baron EC, Breuer E, Chandra P, et al. Social determinants of mental disorders and the Sustainable Development Goals: a systematic review of reviews. Lancet Psychiatry. 2018;5(4):357–69.

23. Moitra M, Owens S, Hailemariam M, Wilson KS, Mensa-Kwao A, Gonese G, et al. Global mental health: where we are and where we are going. Curr Psychiatry Rep. 2023;25(7):301–11.

24. Deglon M, Dalvie MA, Abrams A. The impact of extreme weather events on mental health in Africa: a scoping review of the evidence. Sci Total Environ. 2023; 881:163420.

25. Hawkins C, Bwanika JM, Ibanda M. Socio-economic factors associated with mental health disorders in Fort Portal, western Uganda. S Afr J Psychiatr. 2020; 26:1391.

26. Musisi S, Kinyanda E. Long-Term Impact of War, Civil War, and Persecution in Civilian Populations—Conflict and Post-Traumatic Stress in African Communities. Frontiers in Psychiatry [Internet]. 2020 [cited 2023 Oct 23];11. Available from: https://www.frontiersin.org/articles/10.3389/fpsyt.2020.00020

For peer review only - http://bmjopen.bmj.com/site/about/guidelines.xhtml

27. Robertson LJ. The impact of urbanization on mental health service provision: a Brazil, Russia, India, China, South Africa and Africa focus. Curr Opin Psychiatry. 2019;32(3):224.

28. World Health Organization. World mental health report: Transforming mental health for all [Internet]. Geneva; 2022 [cited 2023 May 8]. Available from: https://www.who.int/publications-detail-redirect/9789240049338

29. Furber G, Leach M, Guy S, Segal L. Developing a broad categorisation scheme to describe risk factors for mental illness, for use in prevention policy and planning. Aust N Z J Psychiatry. 2017;51(3):230–40.

30. Yu S. Uncovering the hidden impacts of inequality on mental health: a global study. Transl Psychiatry. 2018;8(1):98.

31. Adhvaryu A, Fenske J, Nyshadham A. Early life circumstance and adult mental health. J Polit Econ. 2019;127(4):1516–49.

32. Oppong S, Kretchy IA, Imbeah EP, Afrane BA. Managing mental illness in Ghana: the state of commonly prescribed psychotropic medicines. Int J Ment Health Syst. 2016;10(1):28.

33. Eaton J, Ohene S. Providing Sustainable Mental Health Care in Ghana: A Demonstration Project. In: Providing Sustainable Mental and Neurological Health Care in Ghana and Kenya: Workshop Summary [Internet]. National Academies Press (US); 2016 [cited 2023 Jun 9]. Available from: https://www.ncbi.nlm.nih.gov/books/NBK350318/

34. de-Graft Aikins A, Koram K. Health and Healthcare in Ghana, 1957-2017. In: The Economy of Ghana: Sixty Years after Independence. Oxford University Press; 2017.

35. Government of Ghana. Mental Health Policy 2019 -2030: Ensuring A Mentally Healthy Population [Internet]. 2018 [cited 2023 May 8]. Available from: https://extranet.who.int/mindbank/item/7134

36. Amu H, Osei E, Kofie P, Owusu R, Bosoka SA, Konlan KD, et al. Prevalence and predictors of depression, anxiety, and stress among adults in Ghana: a community-based cross-sectional study. PLoS One. 2021;16(10): e0258105.

37. Atuoye KN, Luginaah I. Food as a social determinant of mental health among household heads in the Upper West Region of Ghana. Soc Sci Med. 2017; 180:170–80.

38. Greif MJ, Nii-Amoo Dodoo F. How community physical, structural, and social stressors relate to mental health in the urban slums of Accra, Ghana. Health Place. 2015; 33:57–66.

39. Moher D, Shamseer L, Clarke M, Ghersi D, Liberati A, Petticrew M, et al. Preferred reporting items for systematic review and meta-analysis protocols (PRISMA-P) 2015 statement. Syst Rev. 2015;4(1):1.

40. Munn Z, Moola S, Lisy K, Riitano D, Tufanaru C. Methodological guidance for systematic reviews of observational epidemiological studies reporting prevalence and cumulative incidence data. JBI Evid Implement. 2015;13(3):147.

For peer review only - http://bmjopen.bmj.com/site/about/guidelines.xhtml

41. Moola S, Munn Z, Sears K, Sfetcu R, Currie M, Lisy K, et al. Conducting systematic reviews of association (etiology): the Joanna Briggs Institute's approach. JBI Evid Implement. 2015;13(3):163.

42. Steel Z, Marnane C, Iranpour C, Chey T, Jackson JW, Patel V, et al. The global prevalence of common mental disorders: a systematic review and meta-analysis 1980–2013. Int J Epidemiol. 2014;43(2):476–93.

43. Lim ICZY, Tam WWS, Chudzicka-Czupała A, McIntyre RS, Teopiz KM, Ho RC, et al. Prevalence of depression, anxiety and post-traumatic stress in war- and conflict-afflicted areas: a meta-analysis. Front Psychiatry. 2022; 13:978703.

44. Edwards N, Walker S, Paddick SM, Prina AM, Chinnasamy M, Reddy N, et al. Prevalence of depression and anxiety in older people in low- and middle- income countries in Africa, Asia and South America: a systematic review and meta-analysis. J Affect Disord. 2023; 325:656–74.

45. Page MJ, McKenzie JE, Bossuyt PM, Boutron I, Hoffmann TC, Mulrow CD, et al. The PRISMA 2020 statement: an updated guideline for reporting systematic reviews. BMJ. 2021;372: n71.

46. McGowan J, Sampson M, Salzwedel DM, Cogo E, Foerster V, Lefebvre C. PRESS peer review of electronic search strategies: 2015 guideline statement. J Clin Epidemiol. 2016; 75:40–6.

47. Ouzzani M, Hammady H, Fedorowicz Z, Elmagarmid A. Rayyan—a web and mobile app for systematic reviews. Systematic Reviews. 2016;5(1):210.

48. Munn Z, Moola S, Riitano D, Lisy K. The development of a critical appraisal tool for use in systematic reviews addressing questions of prevalence. Int J Health Policy Manag. 2014;3(3):123–8.

49. Opio JN, Munn Z, Aromataris E. Prevalence of mental disorders in Uganda: a systematic review and meta-analysis. Psychiatr Q. 2022;93(1):199–226.

50. Aarah-Bapuah M, Sarpomaa Oppong S, Ohenewaa Yawson A, Dzansi G, Adjorlolo S. Covid-19 and mental health of children and adolescents: a systematic review. Cogent Psychology. 2022;9(1):2111849.

51. Borenstein M. Comprehensive Meta-Analysis Software. In 2022. p. 535–48.

52. Doi SAR, Williams GM. Methods of Clinical Epidemiology. Springer Science & Business Media; 2013. 289 p.

53. Sutton AJ, Song F, Gilbody SM, Abrams KR. Modelling publication bias in meta-analysis: a review. Stat Methods Med Res. 2000;9(5):421–45.

54. Bhattacharya R, Shen C, Sambamoorthi U. Excess risk of chronic physical conditions associated with depression and anxiety. BMC Psychiatry. 2014;14(1):10.

For peer review only - http://bmjopen.bmj.com/site/about/guidelines.xhtml

55. Adu MK, Wallace LJ, Lartey KF, Arthur J, Oteng KF, Dwomoh S, et al. Prevalence and correlates of likely major depressive disorder among the adult population in Ghana during the COVID-19 Pandemic. Int J Environ Res Public Health. 2021;18(13):7106.

56. Ae-Ngibise KA, Sakyi L, Adwan-Kamara L, Lund C, Weobong B. Prevalence of probable mental, neurological and substance use conditions and case detection at primary healthcare facilities across three districts in Ghana: findings from a cross-sectional health facility survey. BMC Psychiatry. 2023;23(1):280.

57. Campion J, Javed A, Lund C, Sartorius N, Saxena S, Marmot M, et al. Public mental health: required actions to address implementation failure in the context of COVID-19. Lancet Psychiatry. 2022;9(2):169–82.

For peer review only

For peer review only - http://bmjopen.bmj.com/site/about/guidelines.xhtml

Prevalence and social determinants of anxiety and depression among adults in Ghana: a systematic review and meta-analysis protocol

PRISMA-P (Preferred Reporting Items for Systematic review and Meta-Analysis Protocols) 2015 checklist: recommended items to address in a systematic review protocol*

Section and topic	Item No	Checklist item	Location in text (page number)
**ADMINISTRATIVE INFORMATION**			
Title:			
Identification	1a	Identify the report as a protocol of a systematic review	1
Update	1b	If the protocol is for an update of a previous systematic review, identify as such	N/A
Registration	2	If registered, provide the name of the registry (such as PROSPERO) and registration number	2 & 6
Authors:			
Contact	3a	Provide name, institutional affiliation, e-mail address of all protocol authors; provide physical mailing address of corresponding author	1
Contributions	3b	Describe contributions of protocol authors and identify the guarantor of the review	14
Amendments	4	If the protocol represents an amendment of a previously completed or published protocol, identify as such and list changes; otherwise, state plan for documenting important protocol amendments	6
Support:			
Sources	5a	Indicate sources of financial or other support for the review	14
Sponsor	5b	Provide name for the review funder and/or sponsor	14
Role of sponsor or funder	5c	Describe roles of funder(s), sponsor(s), and/or institution(s), if any, in developing the protocol	14
**INTRODUCTION**			
Rationale	6	Describe the rationale for the review in the context of what is already known	5 & 6 &13
Objectives	7	Provide an explicit statement of the question(s) the review will address with reference to participants, interventions, comparators, and outcomes (PICO)	6
**METHODS**			
Eligibility criteria	8	Specify the study characteristics (such as PICO, study design, setting, time frame) and report characteristics (such as years considered, language, publication status) to be used as criteria for eligibility for the review	6 & 7 & 8

For peer review only

Information sources	9	Describe all intended information sources (such as electronic databases, contact with study authors, trial registers or other grey literature sources) with planned dates of coverage	8 & 9
Search strategy	10	Present draft of search strategy to be used for at least one electronic database, including planned limits, such that it could be repeated	9 & supplementary appendix 3
Study records:			
Data management	11a	Describe the mechanism(s) that will be used to manage records and data throughout the review	9
Selection process	11b	State the process that will be used for selecting studies (such as two independent reviewers) through each phase of the review (that is, screening, eligibility and inclusion in meta-analysis)	9
Data collection process	11c	Describe planned method of extracting data from reports (such as piloting forms, done independently, in duplicate), any processes for obtaining and confirming data from investigators	9 & 10
Data items	12	List and define all variables for which data will be sought (such as PICO items, funding sources), any pre-planned data assumptions and simplifications	9 &10
Outcomes and prioritization	13	List and define all outcomes for which data will be sought, including prioritization of main and additional outcomes, with rationale	9 & 10
Risk of bias in individual studies	14	Describe anticipated methods for assessing risk of bias of individual studies, including whether this will be done at the outcome or study level, or both; state how this information will be used in data synthesis	10 & 11 & 12
Data synthesis	15a	Describe criteria under which study data will be quantitatively synthesised	11
	15b	If data are appropriate for quantitative synthesis, describe planned summary measures, methods of handling data and methods of combining data from studies, including any planned exploration of consistency (such as I^2, Kendall's τ)	11
	15c	Describe any proposed additional analyses (such as sensitivity or subgroup analyses, meta-regression)	11&12
	15d	If quantitative synthesis is not appropriate, describe the type of summary planned	12 &13
Meta-bias(es)	16	Specify any planned assessment of meta-bias(es) (such as publication bias across studies, selective reporting within studies)	11
Confidence in cumulative evidence	17	Describe how the strength of the body of evidence will be assessed (such as GRADE)	N/A

N/A not applicable

*** It is strongly recommended that this checklist be read in conjunction with the PRISMA-P Explanation and Elaboration (cite when available) for important clarification on the items. Amendments to a review protocol should be tracked and dated. The copyright for PRISMA-P (including checklist) is held by the PRISMA-P Group and is distributed under a Creative Commons Attribution Licence 4.0.**

From: Shamseer L, Moher D, Clarke M, Ghersi D, Liberati A, Petticrew M, Shekelle P, Stewart L, PRISMA-P Group. Preferred reporting items for systematic review and meta-analysis protocols (PRISMA-P) 2015: elaboration and explanation. BMJ. 2015 Jan 2;349(jan02 1):pg7647.

For peer review only

Supplementary Appendix 2

PRESS Guideline — Search Submission & Peer Review Assessment

SEARCH SUBMISSION: THIS SECTION TO BE FILLED IN BY THE SEARCHER

Searcher: Victoria Awortwe	Email: victoria.awortwe@uu.se
Date submitted: 31/8/2023	Date requested by:11/9/2023

Systematic Review Title:

Prevalence and social determinants of anxiety and depression among adults in Ghana: a systematic review and meta-analysis protocol

This search strategy is …

X	My PRIMARY (core) database strategy — First time submitting a strategy for search question and database
	My PRIMARY (core) strategy — Follow-up review NOT the first time submitting a strategy for search question and database. If this is a response to peer review, itemize the changes made to the review suggestions
	SECONDARY search strategy— First time submitting a strategy for search question and database
	SECONDARY search strategy — NOT the first time submitting a strategy for search question and database. If this is a response to peer review, itemize the changes made to the review suggestions

Database

(i.e., MEDLINE, CINAHL…): *[mandatory]*

MEDLINE

Interface

(i.e., Ovid, EBSCO…): *[mandatory]*

PubMed

Research Question

(Describe the purpose of the search) *[mandatory]*

For peer review only - http://bmjopen.bmj.com/site/about/guidelines.xhtml

This review aims (1) examine the prevalence of anxiety, depression, and psychological distress among adults in Ghana and (2) explore the social determinants potentially associated with anxiety, depression and psychological distress.

CoCoPop Format

(Outline the CoCoPop for your question — i.e., Condition, Context, Population, and Study Design — as applicable)

**Population**	Adults (individuals aged ≥ 18 years) living in Ghana
**Condition**	Anxiety (i.e. anxiety disorders, anxiety symptoms), depression (i.e. depression, depressive disorders), and psychological distress.
**Context**	Ghana, with adults sampled from the community or clinical settings (e.g., primary health care facilities, hospitals).
**Study design**	Observational study designs

Inclusion Criteria

(List criteria such as age groups, study designs, etc., to be included) *[optional]*

Population: Adults (aged ≥ 18 years) living in Ghana will be eligible for inclusion.

Condition: Anxiety and/or depression assessed using structured diagnostic clinical interview in accordance with the International Classification of Diseases and Related Health Problems (ICD-10 or ICD-11), or the Diagnostic and Statistical Manual of Mental disorders (DSM) third, fourth and fifth edition (DSM-III, DSM-IV or DSM-V); OR clinician or proxy administered screening tool for anxiety, depression and psychological distress. For the meta-analyses, studies reporting on the prevalence of anxiety, depression and psychological distress among adults in Ghana, measured using validated instruments will be included.

Context: Studies from Ghana, with adults sampled from the community or clinical settings (e.g., primary health care facilities, hospitals). Studies conducted in regions that encompass Ghana (e.g., West Africa and Sub-Saharan Africa) will be considered eligible if data on participants living in Ghana is available. No date restriction will be imposed and only studies published in English and Ghanaian languages (e.g., Fante, Ga, Twi) will be considered for inclusion.

Study design: Primary quantitative studies, with observational study designs including longitudinal cohort studies, case-control, and cross-sectional studies. Mixed method studies will be eligible for inclusion only if data from the quantitative component can be clearly extracted.

For peer review only

Exclusion Criteria

(List criteria such as study designs, date limits, etc., to be excluded) *[optional]*

Population: Studies solely focused on children/adolescents will be excluded. Studies focusing on both adults and adolescents will be excluded if they do not present data separately for adults and adolescents or data cannot be obtained via correspondence with study authors. Studies conducted with specific sub-populations including individuals with known psychiatric conditions, prisoners, individuals accused of witchcraft, and women with fertility or gynaecological disorders, will be excluded due to their unique circumstances, which predispose them to a higher risk or potentially elevate the likelihood of experiencing mental health problems compared to the general population.

Condition: Studies solely focused on other mental disorders (e.g., psychotic disorders, bipolar affective disorders), substance use and neurological disorders (e.g., alcohol dependence, multiple sclerosis) will be excluded. Studies will be excluded if the prevalence of anxiety, depression and psychological distress cannot be calculated, e.g., when reported solely as mean score or due to insufficient data.

Context: Studies that were conducted immediately after conflict, humanitarian crises, or natural disaster will be excluded.

Study design: Studies such as case reports, commentaries, conference proceedings, editorials, letters, opinion papers, qualitative studies, reviews, theses/dissertations; and studies published in languages other than English and Ghanaian will be excluded.

Was a search filter applied?

Yes ☐ No ■

If YES, which one(s) (e.g., Cochrane RCT filter, PubMed Clinical Queries filter)? Provide the source if this is a published filter. *[mandatory if YES to previous question — textbox]*

Other notes or comments you feel would be useful for the peer reviewer? ***[optional]***

We did not include search terms regarding (1) prevalence, as that is not the only area of interest; and (2) adults, as the term can be referred to in a variety of ways. Furthermore, we searched Ghana in All fields to accommodate studies conducted in regions that encompass Ghana (e.g., West Africa and Sub-Saharan Africa) providing data on participants living in Ghana.

Please copy and paste your search strategy here, exactly as run, including the number of hits per line. ***[mandatory]***

For peer review only

Database: PubMed. Final number of results from the full search 1297	
**1: Mental disorders**	**2: Ghana**
**Title & Abstract**<br>Mental health 228145<br>Mental disorders 51217<br>Mental disorder 11824<br>Mental illness 37257<br>Mental illnesses 6045<br>Anxiety 265049<br>Anxious 20603<br>Depress* 569151<br>Dysthymia 2320<br>Melancholy 426<br>Mood 92155<br>Affective disorder 7725<br>Affective symptoms 2417<br>Negative affect 13573<br>distress 154761<br>Emotional stress 5232<br><br>**MeSH terms**<br>Mental health 61933<br>Mental disorders 1436875<br>Anxiety 112111<br>Anxiety disorders 90630<br>Depression 257186<br>Depressive disorder 122190<br>Mood disorders 170032<br>Psychological Distress 6832	**All fields**<br>Ghana 24845<br><br>**MeSH term**<br>Ghana 11236
**Total for concept 1:**	**Total for concept 2:**
**2183845**	**25013**

Total for complete search: 1297

For peer review only

Raw PubMed Search

("mental health"[Title/Abstract] OR "mental disorders"[Title/Abstract] OR "mental disorder"[Title/Abstract] OR "mental illness"[Title/Abstract] OR "mental illnesses"[Title/Abstract] OR "anxiety"[Title/Abstract] OR "anxious"[Title/Abstract] OR "depress*"[Title/Abstract] OR "dysthymia"[Title/Abstract] OR "melancholy"[Title/Abstract] OR "mood"[Title/Abstract] OR "affective disorder"[Title/Abstract] OR "affective symptom"[Title/Abstract] OR "negative affect"[Title/Abstract] OR "distress"[Title/Abstract] OR "emotional stress"[Title/Abstract] OR ("mental health"[MeSH Terms] OR "mental disorders"[MeSH Terms] OR "anxiety"[MeSH Terms] OR "anxiety disorders"[MeSH Terms] OR ("depressive disorder"[MeSH Terms] OR "depression"[MeSH Terms]) OR "depressive disorder"[MeSH Terms] OR "mood disorders"[MeSH Terms] OR "psychological distress"[MeSH Terms])) AND ("Ghana"[MeSH Terms] OR "Ghana"[All Fields] OR "Ghana s"[All Fields] OR "Ghana"[MeSH Terms]).

For peer review only

PEER REVIEW ASSESSMENT: THIS SECTION TO BE FILLED IN BY THE REVIEWER

Reviewer: Professor Alkistis Skalkidou	Email: Alkistis.skalkidou@kbh.uu.se	Date completed: 14/9/2023

1. TRANSLATION

A ---No revisions	■
B --- Revision(s) suggested	☐
C --- Revision(s) required	☐

If "B" or "C," please provide an explanation or example:

For peer review only

Does the search strategy match the research question/PICO?
Are the search concepts clear?
Are there too many or too few PICO elements included?
Are the search concepts too narrow or too broad?
Does the search retrieve too many or too few records? (Please show number of hits per line.)
Are unconventional or complex strategies explained

2. BOOLEAN AND PROXIMITY OPERATORS

A ---No revisions	■
B --- Revision(s) suggested	☐
C --- Revision(s) required	☐

If "B" or "C," please provide an explanation or example:

Are Boolean or proximity operators used correctly?
Is the use of nesting with brackets appropriate and effective for the search?
If NOT is used, is this likely to result in any unintended exclusions?
Could precision be improved by using proximity operators (eg, adjacent, near, within) or phrase searching instead of AND?
Is the width of proximity operators suitable (eg, might adj5 pick up more variants than adj2)?
Not used

3. SUBJECT HEADINGS

A ---No revisions	☐
B --- Revision(s) suggested	■
C --- Revision(s) required	☐

If "B" or "C," please provide an explanation or example:

Are the subject headings relevant?
Are any subject headings too broad or too narrow?
Are subject headings exploded where necessary and vice versa?
Are major headings ("starring" or restrict to focus) used? If so, is there adequate justification?
Are subheadings missing?
Are subheadings attached to subject headings? (Floating subheadings may be preferred.)
Are floating subheadings relevant and used appropriately?
Are both subject headings and terms in free text (see the following) used for each concept?

For peer review only

4. TEXT WORD SEARCHING

A ---No revisions	■
B --- Revision(s)suggested	☐
C --- Revision(s) required	☐

If "B" or "C," please provide an explanation or example:

Does the search include all spelling variants in free text (eg, UK vs. US spelling)?
Does the search include all synonyms or antonyms (eg, opposites)?
Does the search capture relevant truncation (i.e., is truncation at the correct place)?
Is the truncation too broad or too narrow?
Are acronyms or abbreviations used appropriately? Do they capture irrelevant material? Are the full terms also included?
Have the appropriate fields been searched; for example, is the choice of the text word fields (.tw.) or all fields (.af.) appropriate? Are there any other fields to be included or excluded (database specific)?
Should any long strings be broken into several shorter search statements?

5. SPELLING, SYNTAX, AND LINE NUMBERS

A ---No revisions	■
B --- Revision(s)suggested	☐
C --- Revision(s) required	☐

If "B" or "C," please provide an explanation or example:

Are there any spelling errors?
Are there errors in system syntax (e.g. the use of a truncation symbol from a different search interface)?
Are there incorrect line combinations or orphan lines (e.g. lines that are not referred to in the final summation that?
could indicate an error in an AND or OR statement)?

6. LIMITS AND FILTERS

A ---No revisions	☐

B --- Revision(s) suggested	■
C --- Revision(s) required	☐

If "B" or "C," please provide an explanation or example:

Are all limits and filters used appropriately and are they relevant given the research question?

Are all limits and filters used appropriately and are they relevant for the database?

Are any potentially helpful limits or filters missing? Are the limits or filters too broad or too narrow? Can any limits

or filters be added or removed? Language? Time period? Only humans?

Are sources cited for the filters used?

Author response:

Thanks for the suggestion. Databases will be searched from inception, and the time period for data collection (e.g., <2020 vs. ≥2020) will be investigated as a methodological moderator of prevalence utilising subgroup analyses and meta-regression. Only human studies and language search filters were not used. However, non-human studies and studies published in languages other than English and Ghanaian would be excluded during the screening phase.

OVERALL EVALUATION (Note: If one or more "revision required" is noted above, the response below must be "revisions required".)

A ---No revisions	☐
B --- Revision(s) suggested	■
C --- Revision(s) required	☐

Additional comments:

I am not familiar with Medline, but many within psychology also search the PSYCHINFO database

Author response:

Thank you for your feedback. MEDLINE (PubMed) is one of the electronic databases in which the searches will be conducted. However, electronic database searches will be also carried out in African Index Medicus (AIM), African Journals Online (AJOL), Cumulative Index to Nursing and Allied Health Literature (CINAHL), Excerpta Medica Database (Embase), Ghana Medical Journal (GMJ), Health Sciences Investigation (HIS), **PsycINFO** and SCOPUS.

For peer review only - http://bmjopen.bmj.com/site/about/guidelines.xhtml

PEER REVIEW ASSESSMENT #2: THIS SECTION TO BE FILLED IN BY THE REVIEWER

Reviewer: Associate Professor Lene Lindberg	Email: lene.lindberg@ki.se	Date completed:11/9/2023

1. TRANSLATION

A ---No revisions	☐
B --- Revision(s) suggested	■
C --- Revision(s) required	☐

If "B" or "C," please provide an explanation or example: See my questions above

Does the search strategy match the research question/PICO?
Are the search concepts clear?
Are there too many or too few PICO elements included?
Are the search concepts too narrow or too broad? Mental disorders is too broad?
Does the search retrieve too many or too few records? (Please show number of hits per line.)
Are unconventional or complex strategies explained

For peer review only

2. BOOLEAN AND PROXIMITY OPERATORS

A ---No revisions	■
B --- Revision(s) suggested	☐
C --- Revision(s) required	☐

If "B" or "C," please provide an explanation or example:

Are Boolean or proximity operators used correctly?
Is the use of nesting with brackets appropriate and effective for the search?
If NOT is used, is this likely to result in any unintended exclusions?
Could precision be improved by using proximity operators (eg, adjacent, near, within) or phrase searching instead of AND?
Is the width of proximity operators suitable (eg, might adj5 pick up more variants than adj2)?

3. SUBJECT HEADINGS

A ---No revisions	■
B --- Revision(s) suggested	☐
C --- Revision(s) required	☐

If "B" or "C," please provide an explanation or example: See my comment above

Are the subject headings relevant?
Are any subject headings too broad or too narrow?

Are subject headings exploded where necessary and vice versa?
Are major headings ("starring" or restrict to focus) used? If so, is there adequate justification?
Are subheadings missing?
Are subheadings attached to subject headings? (Floating subheadings may be preferred.)
Are floating subheadings relevant and used appropriately?
Are both subject headings and terms in free text (see the following) used for each concept?

4. TEXT WORD SEARCHING

A ---No revisions	■
B --- Revision(s)suggested	☐
C --- Revision(s) required	☐

If "B" or "C," please provide an explanation or example:

Does the search include all spelling variants in free text (eg, UK vs. US spelling)?
Does the search include all synonyms or antonyms (eg, opposites)?
Does the search capture relevant truncation (i.e., is truncation at the correct place)?
Is the truncation too broad or too narrow?
Are acronyms or abbreviations used appropriately? Do they capture irrelevant material? Are the full terms also included?
Have the appropriate fields been searched; for example, is the choice of the text word fields (.tw.) or all fields (.af.) appropriate? Are there any other fields to be included or excluded (database specific)?
Should any long strings be broken into several shorter search statements?

5. SPELLING, SYNTAX, AND LINE NUMBERS

A ---No revisions	■
B --- Revision(s)suggested	☐
C --- Revision(s) required	☐

If "B" or "C," please provide an explanation or example:

Are there any spelling errors?
Are there errors in system syntax (e.g. the use of a truncation symbol from a different search interface)?
Are there incorrect line combinations or orphan lines (e.g. lines that are not referred to in the final summation that?
could indicate an error in an AND or OR statement)?

6. LIMITS AND FILTERS

A ---No revisions	■
B --- Revision(s) suggested	☐
C --- Revision(s) required	☐

For peer review only

If "B" or "C," please provide an explanation or example:

Are all limits and filters used appropriately and are they relevant given the research question?<br>Are all limits and filters used appropriately and are they relevant for the database?<br>Are any potentially helpful limits or filters missing? Are the limits or filters too broad or too narrow? Can any limits<br>or filters be added or removed?<br>Are sources cited for the filters used?

OVERALL EVALUATION (Note: If one or more "revision required" is noted above, the response below must be "revisions required".)

A ---No revisions	■
B --- Revision(s) suggested	☐
C --- Revision(s) required	☐

Additional comments:

Any specific time period for the included studies?

Co-morbidity between depression and anxiety common, how will you treat data on that?

Author response:

Thanks for your feedback. Databases will be searched from inception. If data allows, we will explore sources of heterogeneity via subgroup analysis of the moderating effect of time period of data collection on prevalence (e.g., <2020 vs. ≥ 2020).

We will analyze prevalence estimates of anxiety, depression or a combined estimate of their symptom (i.e. psychological distress) separately. We could consider data on comorbidity between anxiety and depression and this would involve presenting the percentage of adults who experience both conditions simultaneously. Any protocol amendments will be recorded in PROSPERO.

For peer review only

For peer review only - http://bmjopen.bmj.com/site/about/guidelines.xhtml

Prevalence and social determinants of anxiety and depression among adults in Ghana: a systematic review and meta-analysis protocol

Supplementary Appendix 3: Search strategies-electronic databases

PubMed

#	Searches	Comment
1	Mental health [Title/Abstract] OR<br>Mental disorders [Title/Abstract] OR<br>Mental disorder [Title/Abstract] OR<br>Mental illness [Title/Abstract] OR<br>Mental illnesses [Title/Abstract] OR<br>Anxiety [Title/Abstract] OR<br>Anxious [Title/Abstract] OR<br>Depress*[Title/Abstract] OR<br>Dysthymia [Title/Abstract] OR<br>Melancholy [Title/Abstract] OR<br>Mood [Title/Abstract] OR<br>Affective disorder [Title/Abstract] OR<br>Affective symptoms [Title/Abstract] OR<br>Negative affect [Title/Abstract] OR<br>distress [Title/Abstract] OR<br>Emotional stress [Title/Abstract] OR	
2	Mental health [MeSH] OR<br>Mental disorders [MeSH] OR<br>Anxiety [MeSH] OR<br>Anxiety disorders [MeSH] OR<br>Depression [MeSH] OR<br>Mood disorders [MeSH] OR<br>Psychological Distress [MeSH] OR	
3	1 OR 2	
Ghana		
4	Ghana [All fields]	
5	Ghana [MeSH]	
6	4 OR 5	
Final results	3 AND 6	
syntax	((((((((((((((((Mental health[Title/Abstract]) OR (Mental disorders[Title/Abstract])) OR (mental disorder[Title/Abstract])) OR (mental illness[Title/Abstract])) OR (mental illnesses[Title/Abstract])) OR (anxiety[Title/Abstract])) OR (anxious[Title/Abstract])) OR (depress*[Title/Abstract])) OR (dysthymia[Title/Abstract])) OR	

For peer review only

	(melancholy[Title/Abstract])) OR (mood[Title/Abstract])) OR (affective disorder[Title/Abstract])) OR (affective symptoms[Title/Abstract])) OR (negative affect[Title/Abstract])) OR (distress[Title/Abstract])) OR (emotional stress[Title/Abstract])) OR ((((((((mental health[MeSH Terms]) OR (mental disorders[MeSH Terms])) OR (anxiety[MeSH Terms])) OR (anxiety disorders[MeSH Terms])) OR (depression[MeSH Terms])) OR (depressive disorder[MeSH Terms])) OR (mood disorders[MeSH Terms])) OR (psychological distress[MeSH Terms]))) AND ((Ghana) OR (Ghana[MeSH Terms]))

CINAHL

#	Searches	Comment
1	MW= (mental health OR mental disorders OR anxiety OR anxiety disorders OR depression OR mood disorders OR psychological Distress)	
2	TI= (mental health OR mental disorder*OR mental disorder OR mental illness OR mental illnesses OR anxiety OR anxious OR depress* OR dysthymia OR melancholy OR mood OR affective disorder OR affective symptoms OR negative affect OR distress OR emotional stress).	
3	AB= (mental health OR mental disorder*OR mental disorder OR mental illness OR mental illnesses OR anxiety OR anxious OR depress* OR dysthymia OR melancholy OR mood OR affective disorder OR affective symptoms OR negative affect OR distress OR emotional stress)	In Cinahl Title and Abstract are divided. Search 3 is for abstract OR between terms.
4	1 OR 2 OR 3	
5	MW=(Ghana)	Same as concept 1
6	TI=(Ghana)	
7	AB= (Ghana)	
8	5 OR 6 OR 7	
9 Final results	4 AND 8	
syntax	((MW (Mental health OR mental disorders OR anxiety OR anxiety disorders OR depression OR mood disorders OR psychological Distress) OR TI (Mental health OR mental disorder*OR mental disorder OR mental illness OR mental illnesses OR anxiety OR anxious OR depress* OR dysthymia OR melancholy OR mood OR affective disorder OR affective symptoms OR negative affect OR distress OR emotional stress) OR AB(Mental health OR mental disorder*OR mental disorder OR mental illness OR mental illnesses OR anxiety OR anxious OR depress* OR dysthymia OR melancholy	

For peer review only

	OR mood OR affective disorder OR affective symptoms OR negative affect OR distress OR emotional stress)) AND (MW(Ghana) OR TI(Ghana) OR AB(Ghana))

Embase

#	Searches	Comment
1	TI, AB= (mental health OR mental disorders OR mental disorder OR mental illness OR mental illnesses OR anxiety OR anxious OR depress* OR dysthymia OR melancholy OR mood OR affective disorder OR affective symptoms OR negative affect OR distress OR emotional stress).	
2	/ DE= (mental health OR mental disorders OR anxiety OR anxiety disorders OR depression OR mood disorders OR psychological distress)	Unexploded includes only a single term in hierarchy. [Emtree unexploded] =/de
3	1 OR 2	
4	“Ghana”/exp	
5	“Ghana”. ti, ab.	
6	4 OR 5	
9 Final results	3 AND 6	

PsycINFO

#	Searches	Comment
1	MA= (mental health OR mental disorders OR anxiety OR anxiety disorders OR depression OR mood disorders OR psychological distress)	
2	TI= (Mental health OR mental disorder OR mental disorder OR mental illness OR mental illnesses OR anxiety OR anxious	

	OR depress* OR dysthymia OR melancholy OR mood OR affective disorder OR affective symptoms OR negative affect OR distress OR emotional stress)	
3	AB= (Mental health OR mental disorder OR mental disorder OR mental illness OR mental illnesses OR anxiety OR anxious OR depress* OR dysthymia OR melancholy OR mood OR affective disorder OR affective symptoms OR negative affect OR distress OR emotional stress)	OR between terms.
4	1 OR 2 OR 3	
5	MA= (Ghana)	Same as concept 1
6	TI= (Ghana)	
7	AB= (Ghana)	
8	5 OR 6 OR 7	
9 Final results	4 AND 8	

Scopus

#	**Searches**
1	TITLE-ABS-KEY (mental health OR mental disorder*OR mental illness*OR anxiety disorders OR anxiety OR anxious OR dysthymia OR melancholy OR mood OR affective disorder OR affective symptoms OR negative affect OR distress OR emotional stress OR depress*OR mood disorders OR psychological Distress)
2	TITLE-ABS-KEY(Ghana)
3 Final results	1 AND 2

African Index Medicus (AIM)

#	Searches	Comment
1	TITLE-ABS-SUB (mental health OR mental disorders OR mental disorder OR mental illness OR mental illnesses OR anxiety OR anxious OR depress* OR dysthymia OR melancholy OR mood OR affective disorder OR affective symptoms OR negative affect OR distress OR emotional stress)	Due to no Search history a simplified strategy was employed: In one go, using title, abstract, subject field.
2	TITLE-ABS-SUB (Ghana)	
3 Final results	1 AND 2	

African journals online (AJOL)

#	Searches	Comment
1	Ghana AND ("mental disorders" OR "mental health" OR "anxiety" OR "anxiety disorders" OR "depression" OR "depressive symptoms" OR "psychological distress" OR “mental distress”) site:ajol.info	AJOL has a google search engine. We used Google scholar interface, incognito mode to minimize effect of Google algorithm. The search yields the same results without incognito mode. Cut-off at 5(50 studies), due to fluctuating result from page 6 onwards.

Health Science Investigation (HIS)

#	Searches	Comment
1	(mental disorders OR mental health OR anxiety OR anxiety disorders OR depression OR depressive symptoms OR psychological distress OR mental distress) AND Ghana	HIS has two advanced filters: Published After (2020,2021,2022 or 2023) and Published before (2020, 2021,2022 or 2023). The search yielded no result when no filter was applied. However,

For peer review only

		when, for instance, 2020 was selected for published after and 2023 for published before, the search yielded results. Note: The search did not yield any results when the inverted commas were added to the searches.

Ghana Medical Journal (GMJ)

#	Searches	Comment
1	(mental disorders OR mental health OR anxiety OR anxiety disorders OR depression OR depressive symptoms OR psychological distress OR mental distress) AND Ghana site:ghanamedj.org	GMJ is indexed in AJOL and since both sites use google interface, we searched in Google scholar interface, incognito mode to minimize effect of Google algorithm. The search yields the same results without incognito mode.

For peer review only

Prevalence and social determinants of anxiety and depression among adults in Ghana: a systematic review and meta-analysis protocol

Supplementary Appendix 4: Data extraction form

Study Identification Features	
Study ID/ record number	
Title and aim	
First author last name	
Year of publication	
Publication type or Source of data (e.g., journal or report)	
Study Characteristics	
Age (M, SD) and range	
Gender	
Socioeconomic status	
Chronic physical condition reported (e.g., cancer, diabetes, HIV/AIDs)	
Sample size	
Sampling methods (e.g., convenience sampling, random sampling)	
Geographic region	
Geographical location (rural, urban mixed)	
Recruitment setting (Community or clinical settings	
Study design (e.g., cohort study, cross-sectional study)	
Time period of data collection	
Type of mental health condition(s) assessed (i.e. anxiety, depression, and psychological distress)	
Structured diagnostic clinical interview	Yes/No
Name of diagnostic clinical interview	
Screening tool	Yes/No
Name of screening tool and cutoff scores	
Evidence of validity of the measurement instrument	
Evidence of ethical approval	
Result Summary	
Results Summary Prevalence[a]	
Binary prevalence data (n/N) and percentage with 95% confidence intervals	
Prevalence type (current, period or period or life time)	
Social determinants summary	

For peer review only

Domains of the Social determinants	Proximal factors identified		Distal factors identified	
	Risk factors	Protective factors	Risk factors	Protective factors
Demographic domain				
Economic domain				
Environmental domain				
Neighbourhood domain				
Social and cultural domain				

Reviewer comments, if any?

[a]Prevalence data will be extracted for all conditions, namely, depression, anxiety and psychological distress

For peer review only
